# Double conjugation strategy to incorporate lipid adjuvants into multiantigenic vaccines†
†Electronic supplementary information (ESI) available: Structures, spectroscopic data with ^1^H and C^13^ NMR spectra, HPLC profiles and MS spectra. See DOI: 10.1039/c5sc03859f


**DOI:** 10.1039/c5sc03859f

**Published:** 2016-01-04

**Authors:** Waleed M. Hussein, Tzu-Yu Liu, Pirashanthini Maruthayanar, Saori Mukaida, Peter M. Moyle, James W. Wells, Istvan Toth, Mariusz Skwarczynski

**Affiliations:** a School of Chemistry and Molecular Biosciences , The University of Queensland , Brisbane , QLD 4072 , Australia . Email: m.skwarczynski@uq.edu.au ; Email: i.toth@uq.edu.au; b The University of Queensland Diamantina Institute , The University of Queensland , Translational Research Institute , Brisbane , Australia; c School of Pharmacy , The University of Queensland , Brisbane , QLD 4102 , Australia; d Institute for Molecular Bioscience , The University of Queensland , Brisbane , QLD 4072 , Australia

## Abstract

Conjugation of multiple peptides by their N-termini is a promising technique to produce branched multiantigenic vaccines. We established a double conjugation strategy that combines a mercapto-acryloyl Michael addition and a copper-catalysed alkyne-azide 1,3-dipolar cycloaddition (CuAAC) reaction to synthesise self-adjuvanting branched multiantigenic vaccine candidates. These vaccine candidates aim to treat cervical cancer and include two HPV-16 derived epitopes and a novel self-adjuvanting moiety. This is the first report of mercapto-acryloyl conjugation applied to the hetero conjugation of two unprotected peptides by their N-termini followed by a CuAAC reaction to conjugate a novel synthetic lipoalkyne self-adjuvanting moiety. *In vivo* experiments showed that the most promising vaccine candidate completely eradicated tumours in 46% of the mice (6 out of 13 mice).

## Abstract 2

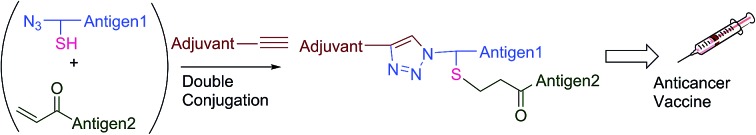
Conjugation of multiple peptides by their N-termini is a promising technique to produce branched multiantigenic vaccines.

## Introduction

The ability to develop safe vaccines using minimal microbial components has triggered rapid growth in research into peptide-based vaccines.^1^ However, the inability of peptides in isolation to stimulate the immune system is one of the key challenges in the development of peptide-based vaccines. Therefore, an adjuvant (immunostimulant) is necessary to stimulate a potent immune response against peptide epitopes.^2^ However, the use of adjuvants is usually associated with side effects and substantial toxicity that has limited the number of adjuvants approved for human use.^3^ Only alum has been approved as a general human adjuvant, while just a few others were approved for particular vaccine formulation *e.g.* MF59, ASO3 and ASO4.^4^ Unfortunately for anticancer vaccines, adjuvants that stimulate safe and effective cytotoxic T lymphocyte (CTL) responses are scarce.^5^ To overcome this problem, peptide vaccine research has turned its focus to the development of self-adjuvanting delivery systems. These vaccines combine peptide epitopes and immunostimulatory moieties (for example lipidic or polymeric entities) in a single covalently-linked conjugate, thereby ensuring co-delivery of the antigen to antigen presenting cells (APCs) activated by immunostimulatory moieties. This combined presentation helps to enhance vaccine potency and to avoid undesirable side effects that result from using classical adjuvants.^6^

Every year, approximately 500 000 women are newly diagnosed with cervical cancer around the world, making it the second most common cancer among women. According to experimental and epidemiological studies, human papilloma virus (HPV) is the main cause of cervical cancer.^7^ Two high-risk genotypes, HPV types 16 (HPV-16) and 18 (HPV-18) are responsible for 70% of all cervical cancers.^8^

Prophylactic vaccines against HPV infection help to reduce the incidence of cervical cancers through the generation of neutralizing antibodies and are only effective if administered before infection with HPV.^9^ Hence, there is a strong demand for the development of effective therapeutic vaccines that are able to treat HPV-related cancers.^10^ The HPV genome encodes two types of proteins: early proteins (E1, E2, E4, E5, E6 and E7) and late proteins (L1 and L2). Expression of the E6 and E7 oncoproteins results in deregulation of the cell cycle, inactivating tumour suppressor gene products p53 and retinoblastoma protein (pRb) and leading to cancer.^10^

Peptide-based strategies to develop therapeutic vaccines against HPV-associated cancers have shown promising outcomes in several early stage clinical trials.^10^ The choice of an appropriate peptide antigen is a crucial issue in the design of synthetic peptide vaccines. Therapeutic vaccines to treat cancer must elicit cellular immunity, thus must include CTL (CD8^+^) epitopes.1*a* A CTL epitope was identified in the HPV-16 E6 protein sequence (QLLRREVYDFAFRDL; E6_43–57_)^10^,^11^ and was previously shown to induce CTLs *in vivo*.^11^,^12^ Recently, our group showed that the 8Q_min_ peptide, a small fragment of HPV-16 E7 protein (QAEPDRAHYNIVTF; E7_44–57_),^9^ that encodes CTL and T-helper cell epitopes, could reduce tumour growth and eradicate E7-expressing TC-1 tumour cells in mice through activation of CTLs^13^ when administered with a self-adjuvanting delivery system. Therefore, these two CTL epitopes, E6_43–57_ and 8Q_min_, were chosen as promising antigens for peptide vaccine development. We also recently demonstrated that anti-8Q_min_ antibodies were not produced by mice vaccinated with the 8Q_min_ epitope conjugated to the poly *tert*-butyl acrylate delivery system.^14^

It was reported that the orientation of antigens in a vaccine conjugate was very important for stimulating an immune response.^15^ Conjugation of different peptides *via* the C-terminus is valuable for the development of multiantigenic branched vaccines. Branched antigens tend to have increased stability to proteolysis,^16^ and therefore a longer circulation time in the host, providing more opportunities to be taken up by APCs. As a result, these peptides can elicit stronger *in vivo* immune responses than linear peptides.^17^ We recently reported that modification of the 8Q_min_ epitope from the E7 protein by replacing the C-terminal CCKCD sequence with SSKSD or SKKKK substantially diminished its immunogenicity. In contrast, deletion of the CCKCD sequence did not have any negative influence on the epitope potency.^9^ These results suggest that the CTL epitope is only effective if conjugated to the vaccine delivery system *via* its N-terminus.

The attachment of a lipidic moiety to the N-terminus of an antigenic peptide to obtain amphiphilic vaccine molecules was previously reported.6c,6d,^18^ However, the N-terminal conjugation of two or more different unprotected epitopes to a vaccine delivery system have not yet been described (to the best of our knowledge). Thus we established a double conjugation synthetic technique to allow the conjugation of different unprotected peptides, E6_43–57_ and 8Q_min_, *via* their N-termini in order to produce novel branched multiantigenic immunotherapeutics.

## Results and discussion

**Scheme 1:**
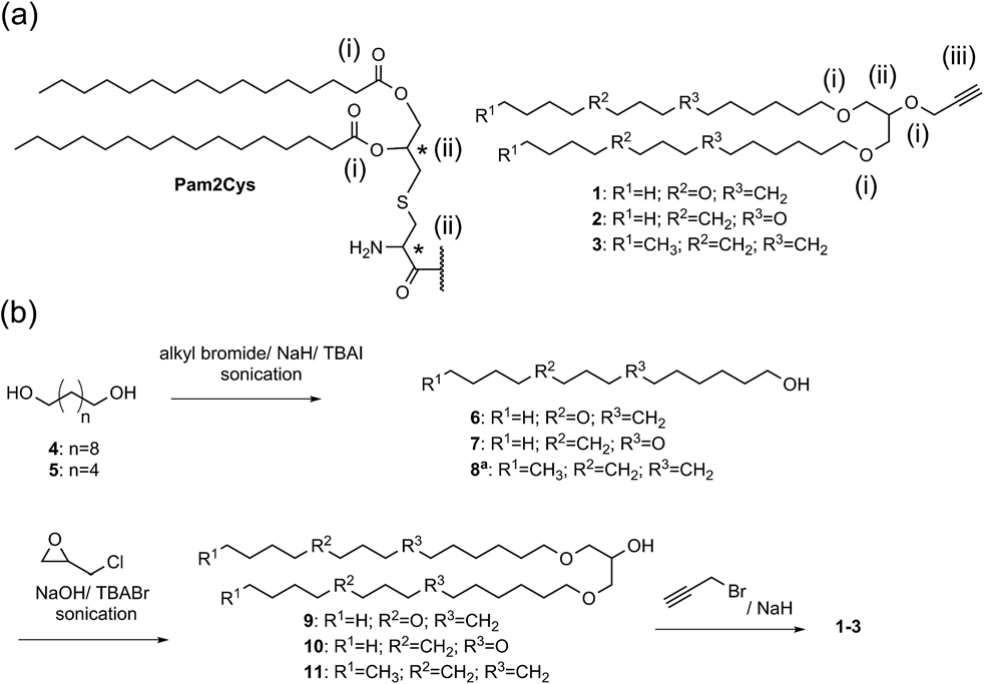
(a) Comparison between the structure of Pam2Cys and the lipoalkynes **1–3**: (i) hydrolyzable ester linkages in Pam2Cys *versus* stable ether bonds in lipoalkynes **1–3**, (ii) two chiral centers in Pam2Cys *versus* achiral molecule in lipoalkynes **1–3**, and (iii) presence of alkyne moiety in lipoalkynes **1–3**. (b) Synthesis of the lipoalkyne vaccine adjuvants **1–3**. ^a^ Compound **8** is a commercially available compound.

We designed and synthesised immunostimulatory lipoalkynes **1–3** (Scheme 1a). These lipoalkynes were based on the structure of Pam2Cys (di-palmitoyl-*S*-glycerol cysteine), a well-characterised self-adjuvanting moiety that is widely used in experimental vaccine design.^19^ As Pam2Cys is a thio-1,2-diglyceride ester of palmitic acid, the new 1,3-diglyceride lipoalkynes **1–3** were designed by replacing the two ester linkages in Pam2Cys with two ether bonds to increase the stability of the compounds against esterases. The two long hydrocarbon chains in Pam2Cys were modified by substituting two methylene groups with oxygen atoms in two different positions as in lipid **1** and **2**, to investigate the effect of increasing the polarity (and subsequently the aqueous solubility) on the adjuvanting effect of the resulting molecules.^20^ For control purposes, lipid **3** contained the same hydrocarbon chain as Pam2Cys was synthesised. In contrast to Pam2Cys, lipids **1–3** have no chiral center and therefore exist as single isomers. They carry an alkyne moiety, thereby allowing easy conjugation of an antigen through a copper-catalysed alkyne-azide 1,3-dipolar cycloaddition (CuAAC) reaction.

Lipoalkynes **1–2** were synthesised using three straightforward steps (Scheme 1b), while lipoalkyne **3** required only two steps to be produced. Alcohols **6–7** were prepared from diols **4–5** in 42 and 44% yields, respectively, using alkyl bromide and a phase-transfer catalyst tetrabutylammonium iodide (TBAI) in presence of DMF as a solvent under sonication conditions. The sonication of a mixture of alcohols **6–8**, powdered sodium hydroxide, epichlorohydrin, and tetrabutylammonium bromide (TBABr) provided the branched alcohols **9–11**. Alcohols **9–11** were treated with propargyl bromide and sodium hydride to afford the lipoalkynes **1–3** in good yields.

**Scheme 2:**
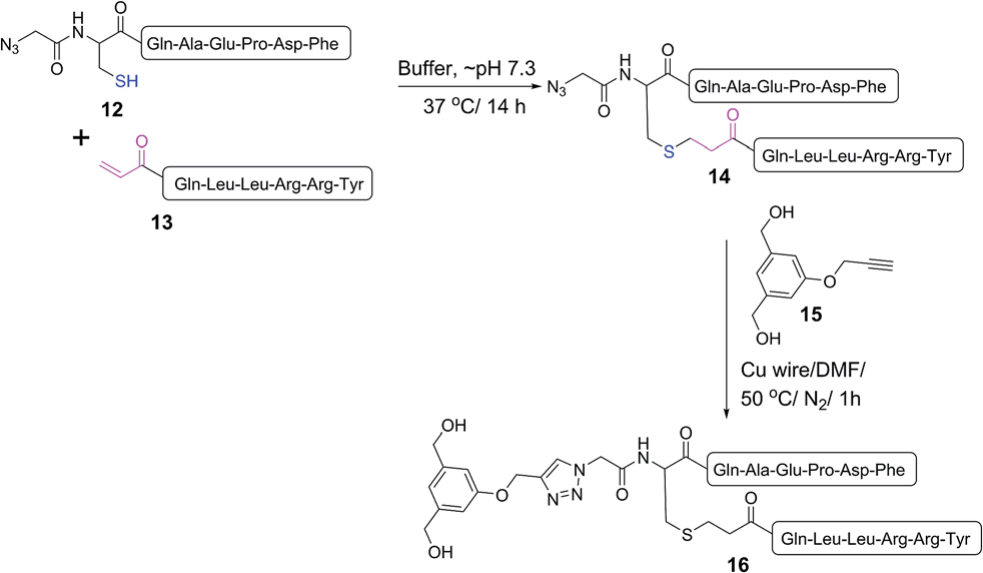
Model double conjugation through a Michael addition, between **12** and **13**, followed by a CuAAC reaction, between **14** and **15**.

A double conjugation strategy was developed to produce anticancer vaccine candidates. Two peptide epitopes were combined into a multiantigenic construct *via* thioether conjugation using an acryloyl peptide. The reaction was examined on two model short peptides (**12** and **13**), where one peptide carried both mercapto and azide groups at its N-terminus (**12**) and the other peptide had an acryloyl moiety attached to its N-terminus (**13**) (Scheme 2).

**Fig. 1:**
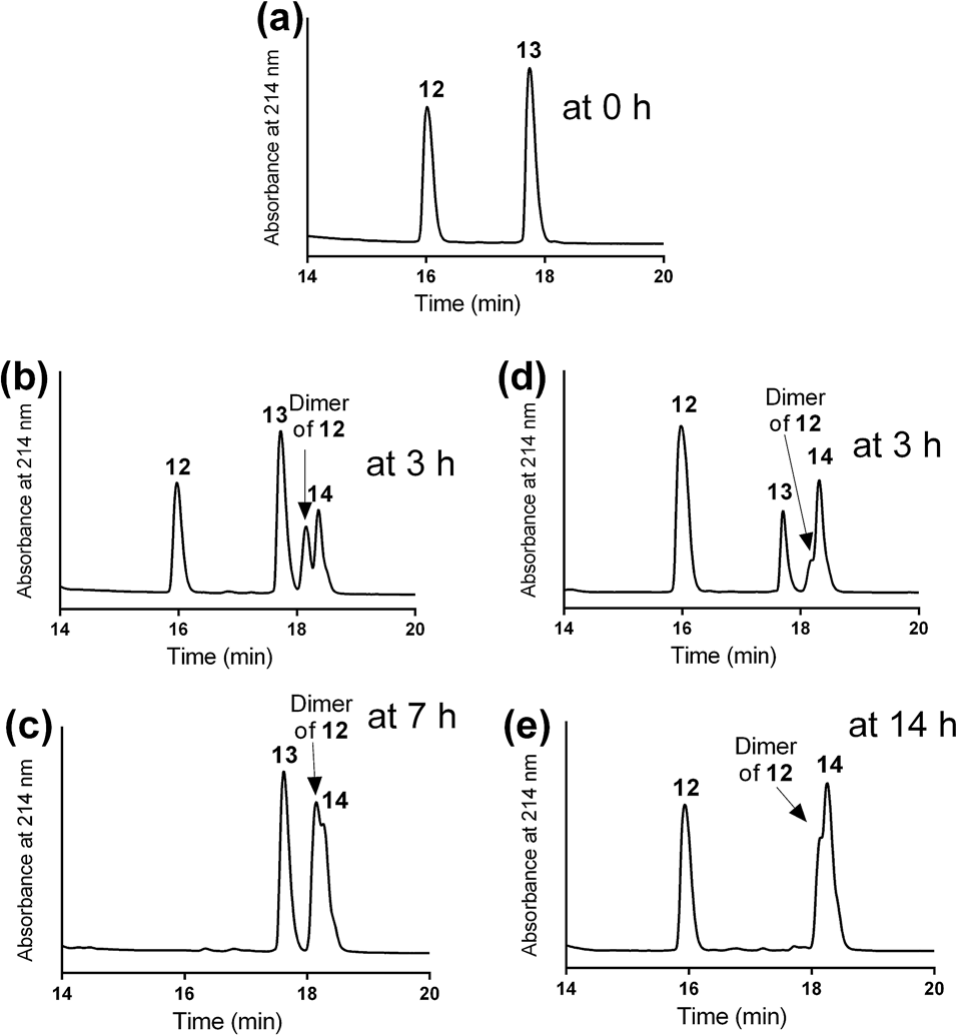
Optimising the conditions for mercapto-acryloyl conjugation between model mercapto-azide (**12**) that used in excess and acryloyl derivative (**13**) peptides (a) at 0 time; (b) at 3 h, using DMF as a solvent and two drops of DIPEA. New products started to form including the mercapto-acryloyl conjugation product (**14**) and the dimer of **12**; (c) at 7 h in DMF/DIPEA the reaction was completed with the formation of peptide **14** and the dimer of **12** as major products with the remaining of the majority of the acryloyl peptide **13**; (d) at 3 h, using guanidine buffer as a solvent (∼pH 7.3) at 37 °C, new products formed, the mercapto-acryloyl conjugation product (**14**) together with the dimer of **12**; (e) at 14 h in a guanidine buffer, the reaction was completed by formation of peptide **14** as a major product together with the dimer of **12** and complete consumption of the acryloyl peptide **13**. The reaction progress was monitored by HPLC and the products were detected by mass spectrometry.

**Fig. 2:**
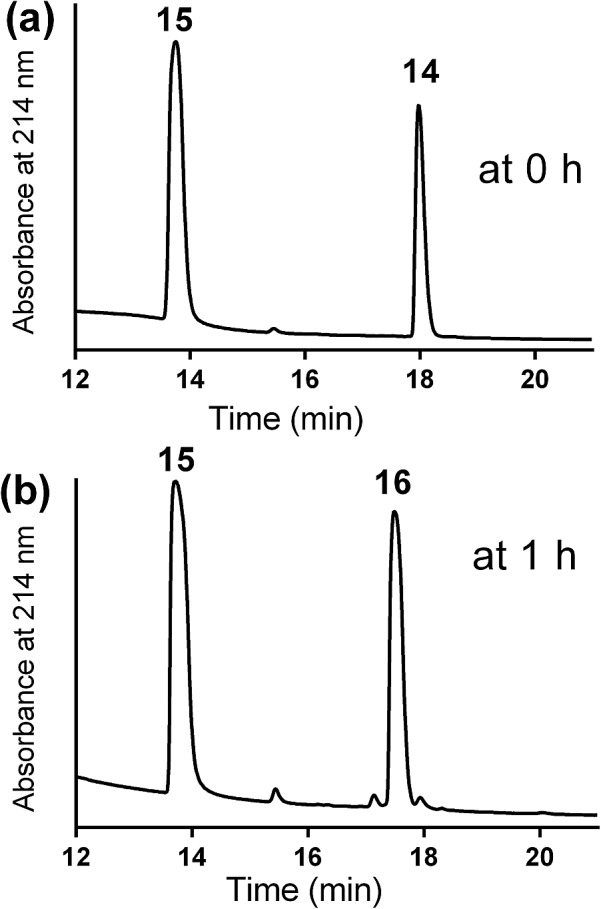
A copper-catalysed alkyne-azide 1,3-dipolar cycloaddition (CuAAC) model reaction between model conjugation product (**14**) and model alkyne (**15**) in DMF in presence of Cu wire at 50 °C under a nitrogen atmosphere (a) at 0 time; (b) at 1 h the reaction was completed by the complete consumption of **14** and formation of the model CuAAC product **16**.

**Scheme 3:**
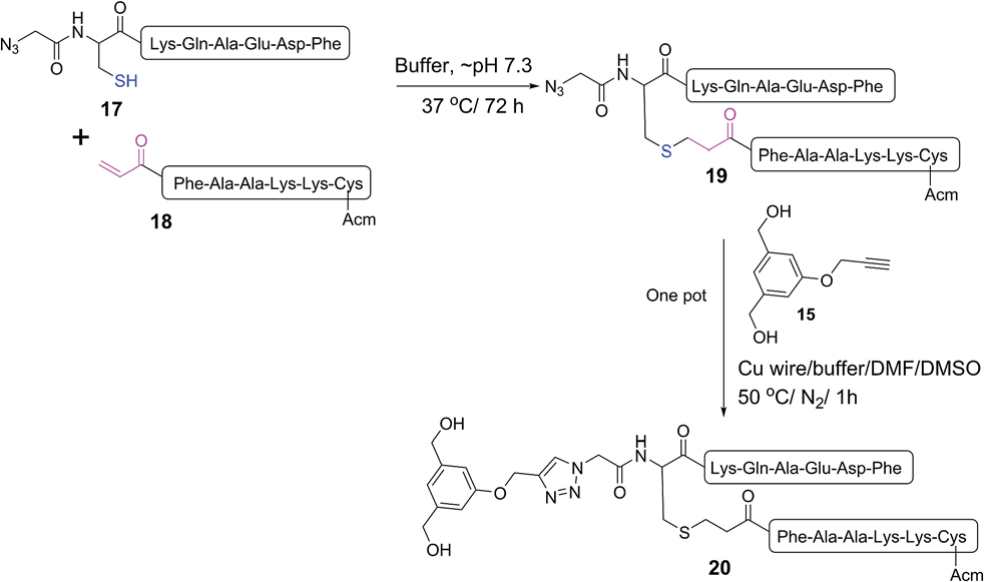
One pot reaction of model double conjugation through mercapto-acryloyl reaction, between **17** and **18** model peptides, followed by azide-alkyne reaction, between compounds **19** and **15**.

**Fig. 3:**
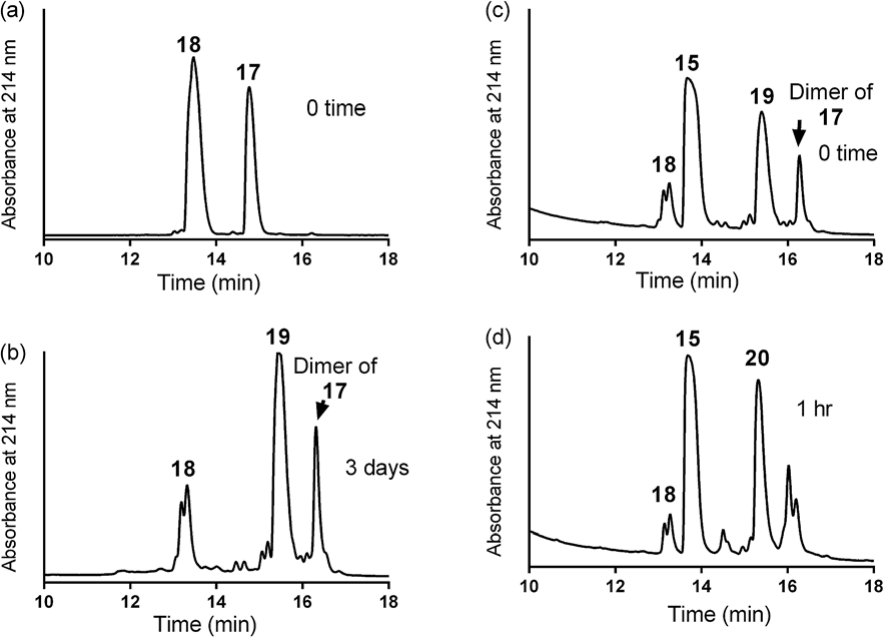
One pot double conjugation model reaction. Mercapto-acryloyl conjugation between mercapto-azide (**17**) and acryloyl derivative (**18**) at ∼pH 7.3 (guanidine buffer), 37 °C (a) at 0 time; (b) at 72 h the reaction was completed by the complete consumption of **17** and formation of the conjugation product (**19**) together with the disulfide dimer of **17**. In a one pot reaction, a CuAAC model reaction between model conjugation product (**19**) and model alkyne (**15**) in guanidine buffer in presence of Cu wire at 50 °C under a nitrogen atmosphere (c) at 0 time; (d) at 1 h the reaction was completed by the complete consumption of **19** and formation of the model CuAAC product **20**. The reaction progress was monitored by HPLC and the products were detected by Mass spec.

The mercapto-acryloyl conjugation conditions were optimised; ∼pH 7.3, 37 °C, 14 h in the presence of denaturants (6 M guanidine) was found to be optimal. The product **14** of this conjugation was reacted with a model alkyne **15** producing the desired conjugate **16** (Scheme 2, Fig. 1 and 2). The ability of double conjugation strategy to be performed in a one pot reaction was also demonstrated (Scheme 3 and Fig. 3).

The new vaccine candidates, lipopeptides **24–26**, were synthesised using the developed conjugation method. First, the N-terminal amine moieties of 8Q_min_ and E6_43–57_ were modified using stepwise SPPS. Fmoc-cysteine and azidoacetic acid were coupled to 8Q_min_ to produce mercapto-azide derivative **21**. The second peptide (E6_43–57_) was modified with acrylic acid to afford acryloyl derivative **22**. The two modified unprotected peptides (**21** and **22**) were then conjugated *via* a Michael addition mercapto-acrylate reaction to produce azide derivative **23**.

**Scheme 4:**
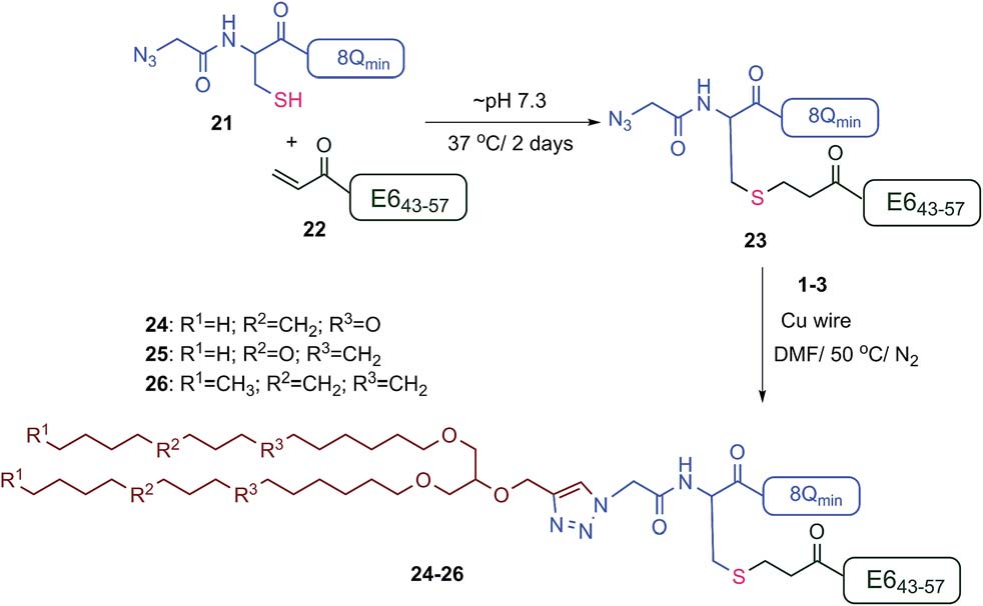
Synthesis of lipopeptides **24–26** using the double conjugation strategy.

**Fig. 4:**
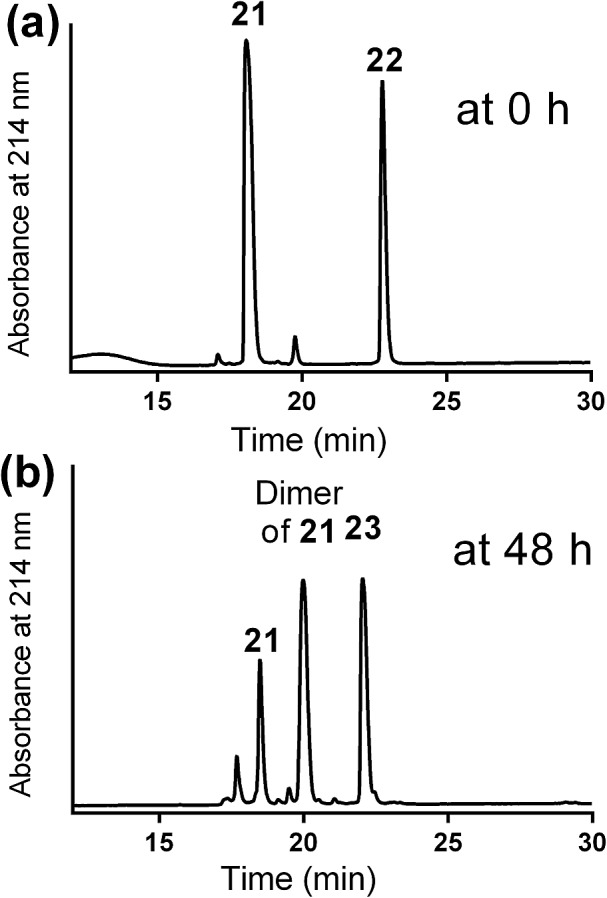
Mercapto-acryloyl conjugation between mercapto-azide (**21**) and acryloyl derivative (**22**) at pH 7.5, 37 °C (a) at 0 time; (b) at 48 h the reaction was completed by the complete consumption of **22** and formation of the multiantigenic conjugation product (**23**) together with the dimer of **21**. The reaction progress was monitored by HPLC and the products were detected by MS.

A solution of mercapto-azide **21** (2 equiv.) and the acryloyl derivative **22** (1 equiv.) were gently shaken in denaturing buffer comprised of 6 M guanidine, 50 mM sodium phosphate, 20% acetonitrile, 5 mM EDTA, at ∼pH 7.3 to afford the azide derivative **23** in an excellent isolated yield of 90% (Scheme 4). The reaction was monitored by analytical HPLC and mass spectroscopy (Fig. 4). The second conjugation between the azide derivative **23** (1 equiv.) and the lipoalkynes **1–3** (1.5 equiv.) was achieved in degassed DMF under a nitrogen atmosphere using the CuAAC reaction in the presence of copper wire6*b* for 4 hours at 50 °C to produce the final lipopeptides **24–26** in 49–87% isolated yields (Scheme 4). The final products **24–26** were structurally well-defined, with only one stereoisomer present, and the synthesis was simple and reproducible.

**Scheme 5:**
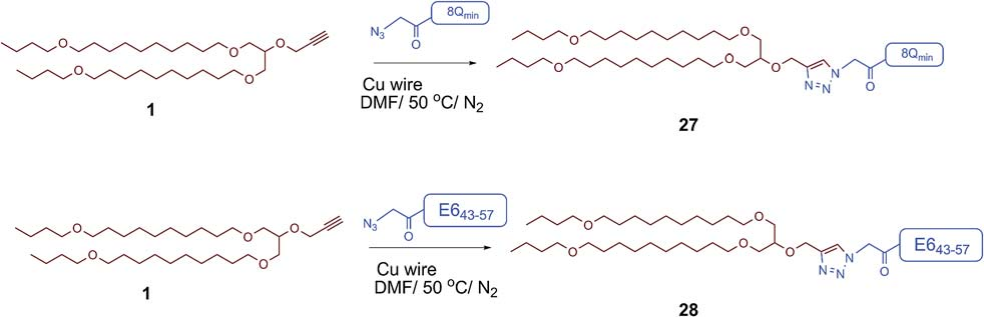
Synthesis of **27** (lipid **1** conjugated with 8Q_min_) and **28** (lipid **1** conjugated with E6_43–57_).

**Scheme 6:**
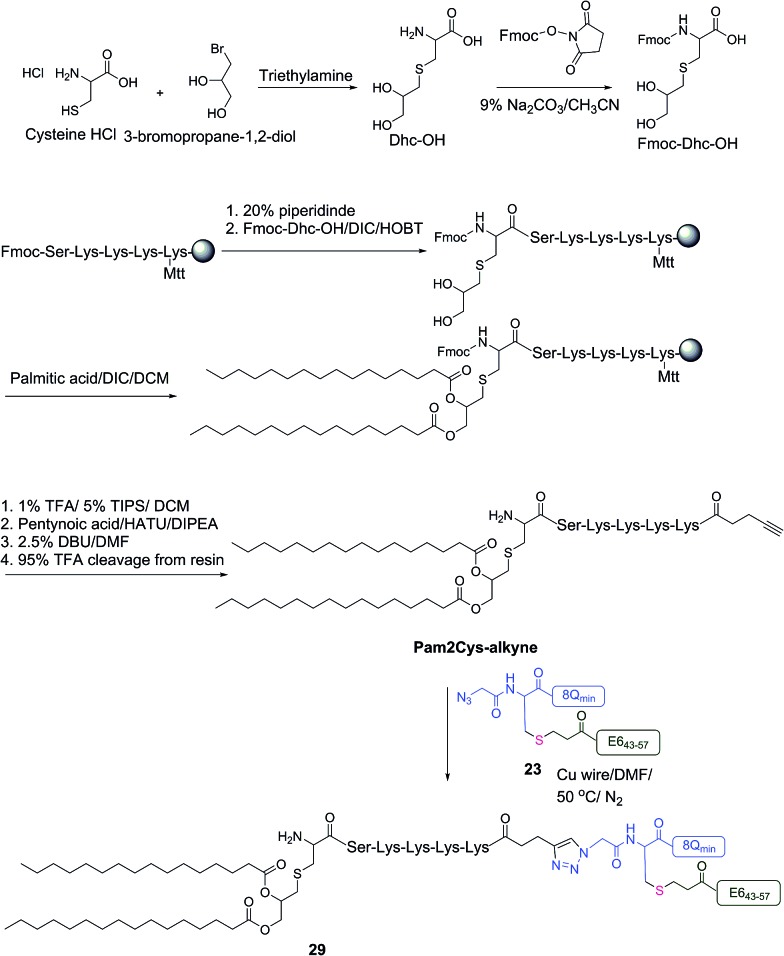
Synthesis of 8Q_min_/E6_43–57_-Pam2Cys (**29**).

**Fig. 5:**
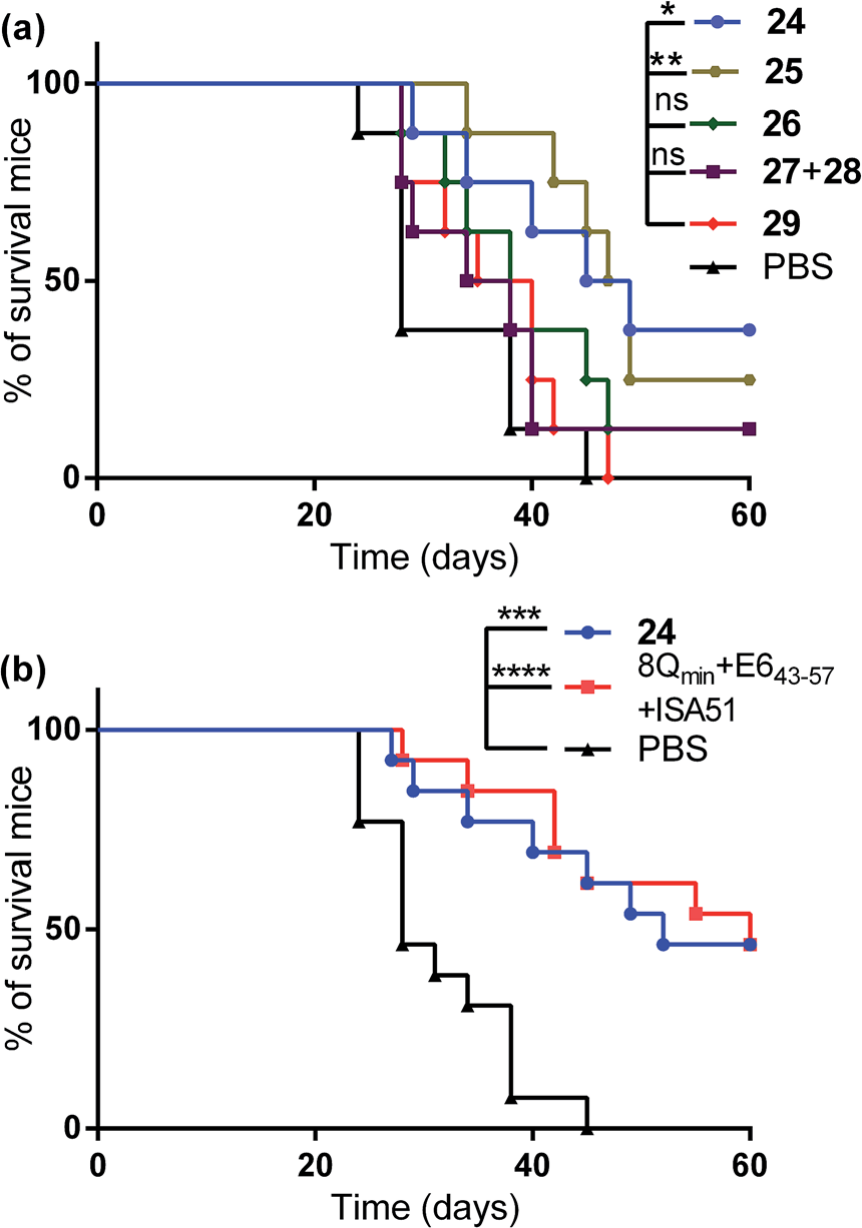
Tumor challenge experiments. C57BL/6 (8/group) were inoculated subcutaneously in the right flank with 1 × 10^5^ TC-1 cells per mouse (day 0) and immunised with: (a) lipopeptides **24–26**; a physical mixture of lipid **1** conjugated with 8Q_min_ (**27**) and lipid **1** conjugated with E6_43–57_ (**28**); 8Q_min_/E6_43–57_ epitopes conjugated with Pam2Cys (**29**) as a positive control; or PBS as a negative control on day 3 (8/group) or (b) lipopeptide **24**; a mixture of 8Q_min_–E6_43–57_ (1 : 1) + ISA51 as a positive control; or PBS as a negative control on day 3 (13/group). Survival rate was monitored over 60 days post implantation and plotted as a Kaplan–Meier survival curve. Mice were euthanised when tumor volume reached 1 cm^3^ or started bleeding. The survival rate of each group was compared to the negative control (PBS) and was analysed using the log-rank (Mantel–Cox) test (*p < 0.05; **p < 0.01; ***p < 0.001; ****p < 0.0001).

**Fig. 6:**
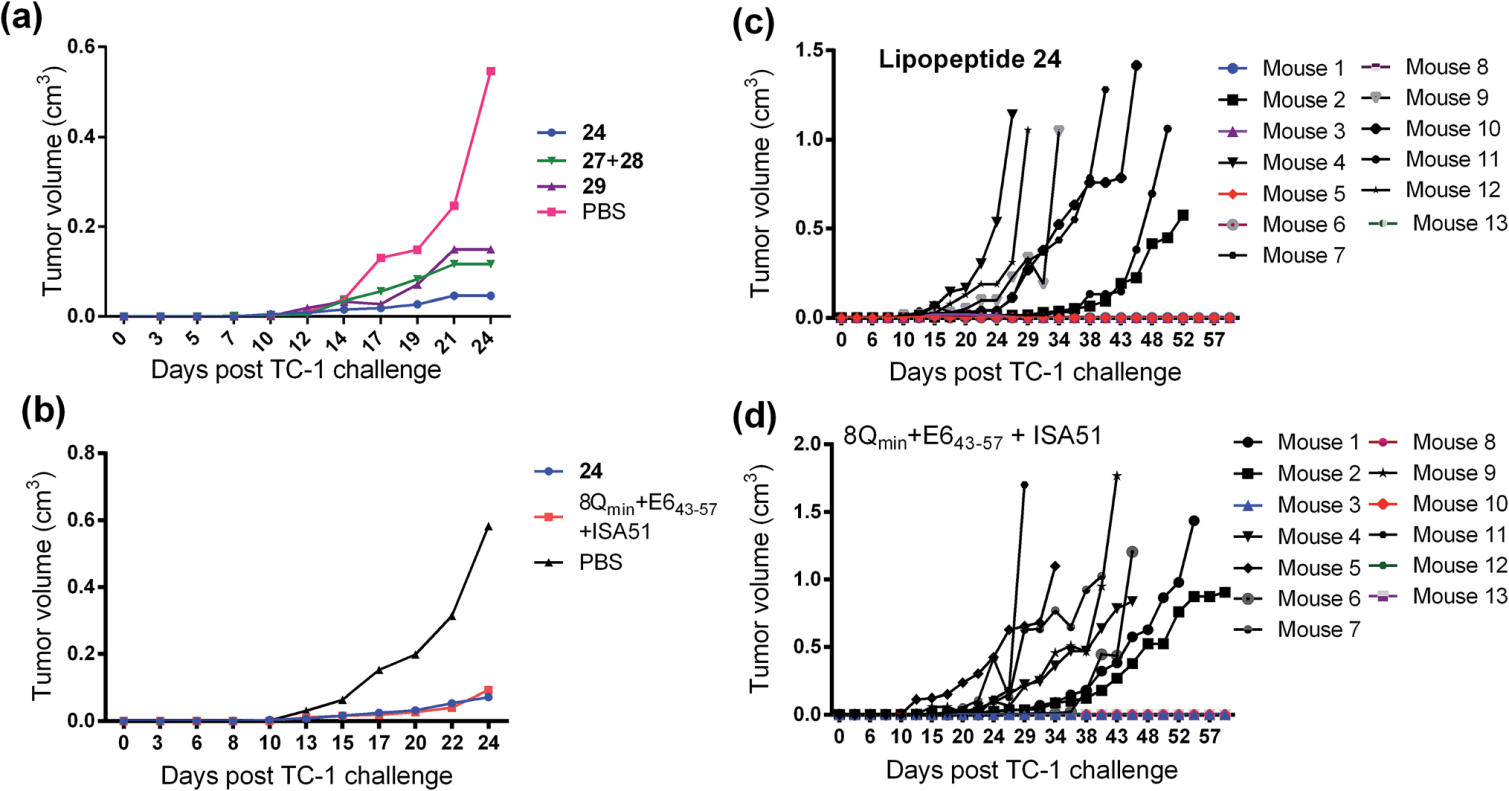
Tumor challenge experiments. (a) C57BL/6 (8 per group) were inoculated subcutaneously in the right flank with 1 × 10^5^ TC-1 tumour cells per mouse (day 0) and immunised with lipopeptide **24**, a mixture of lipid **1** conjugated with 8Q_min_ (**27**) and lipid **1** conjugated with E6_43–57_ (**28**) (1 : 1), 8Q_min_/E6_43–57_-Pam2Cys (**29**) as a positive control, or PBS as a negative control on day 3. (b) C57BL/6 mice (13 per group) were inoculated subcutaneously in the right flank with 1 × 10^5^ TC-1 tumour cells per mouse (day 0) and immunised with lipopeptide **24**, a mixture of 8Q_min_ + E6_43–57_ (1 : 1) + ISA51 as a positive control, or PBS as a negative control on day 3. Mean tumour volume (cm^3^) in different groups of mice shown up to day 24 after tumour implantation (when the first mouse was euthanised). (c) Tumour volume (cm^3^) in individual TC-1 tumour bearing mice (C57BL/6 mice, 13 per group) treated with lipopeptide **24** or (d) 8Q_min_ + E6_43–57_ + ISA51 shown over 60 days post implantation. Mean tumour volume (cm^3^) in different groups of mice (C57BL/6 mice, 13 per group) shown up to day 24 after tumour implantation (when the first mouse was euthanised).

The therapeutic effect of the multiantigenic conjugates on established HPV tumour was evaluated in a mouse model, 6–8 week old, female C57BL/6 mice. The design of compounds **1–3** was based on the structure of Pam2Cys, hence Pam2Cys conjugated to the 8Q_min_/E6_43–57_ epitopes was used as a control (**29**) (Scheme 6). The therapeutic importance of incorporating two epitopes in one molecular entity (**24–26**) was explored by synthesising compounds **27** and **28**, which were comprised of lipid **1** conjugated to 8Q_min_ or E6_43–57_, respectively. At day zero, mice (8/group) were implanted in the side flank with TC-1 tumour cells expressing the E6/E7 oncoproteins.^21^ On day 3 mice were immunised with either lipopeptides **24–26** (100 μg/100 μL sterile PBS), a physical mixture of lipid **1** conjugated with 8Q_min_ (**27**) and lipid **1** conjugated with E6_43–57_ (**28**) (100 μg/100 μL sterile PBS, 1 : 1) (Scheme 5), 8Q_min_/E6_43–57_ epitopes conjugated with Pam2Cys (**29**) (100 μg/100 μL sterile PBS) as a positive control, or PBS (100 μL) as a negative control. The Kaplan–Meier survival curve (Fig. 5a) showed that all of the mice treated with PBS were euthanised due to tumour burden by day 45. In contrast, mice treated with lipopeptide **24** and **25** demonstrated 38% (3 out of 8 mice) and 25% (2 out of 8 mice) survival rates, respectively, which was significantly better than that for mice treated with the positive control (8Q_min_/E6_43–57_-Pam2Cys (**29**), 0% survival, 0 out of 8 mice). Among tested groups, the slowest tumour growth was observed in mice immunised with vaccine candidate **24** (Fig. 6a). The physical mixture of **27** and **28** did not slow down tumour growth significantly and only one mouse treated with the mixture survived to the end of the experiment. We proposed that the physical mixture of **27** and **28** would allow each epitope to be taken up by different APCs, thus the immune stimulating effect of T-helper epitope present in 8Q_min_ may not enhance the immune response against the E6_43–57_ epitope. It was reported that the co-recognition of T-helper and CTL epitopes by the same APC was essential for the efficient stimulation of cellular immunity.^22^ Interestingly, the biological study showed that Pam2Cys analogue (**29**) and the most hydrophobic vaccine candidate **26** induced very weak antitumour responses. In tumour challenge, 0/5 and 1/5 mice survived on day 60 for **29** and **26**, respectively (Fig. 5a) despite the well-proven ability of Pam2Cys to induce cellular immune responses.^23^ This might be explained by the formation of large aggregates (>5 μm in diameter) by conjugates **26** and **29** while compounds **24** and **25** formed particles of submicron size (0.3–0.8 μm as measured by dynamic light scattering) under aqueous conditions. This observation is in the agreement with well-known phenomena that the immune responses are highly dependent on the vaccine particle size.^2^ This size difference may have arisen because the presence of oxygen atoms in the hydrocarbon chain in both lipids **1** and **2** increased the solubility of the latter compounds (**24** and **25**).

Compound **29** (which bore a Pam2Cys moiety) induced unexpectedly weak antitumour responses, therefore two additional independent experiments (with 5 + 8 mice per group) were performed to further investigate the antitumour potency of the lead vaccine candidate. Incomplete Freund's adjuvant (Montanide ISA51) was chosen as an adjuvant in an emulsion with 8Q_min_ and E6_43–57_ epitopes for formulation of the positive control.

**Fig. 7:**
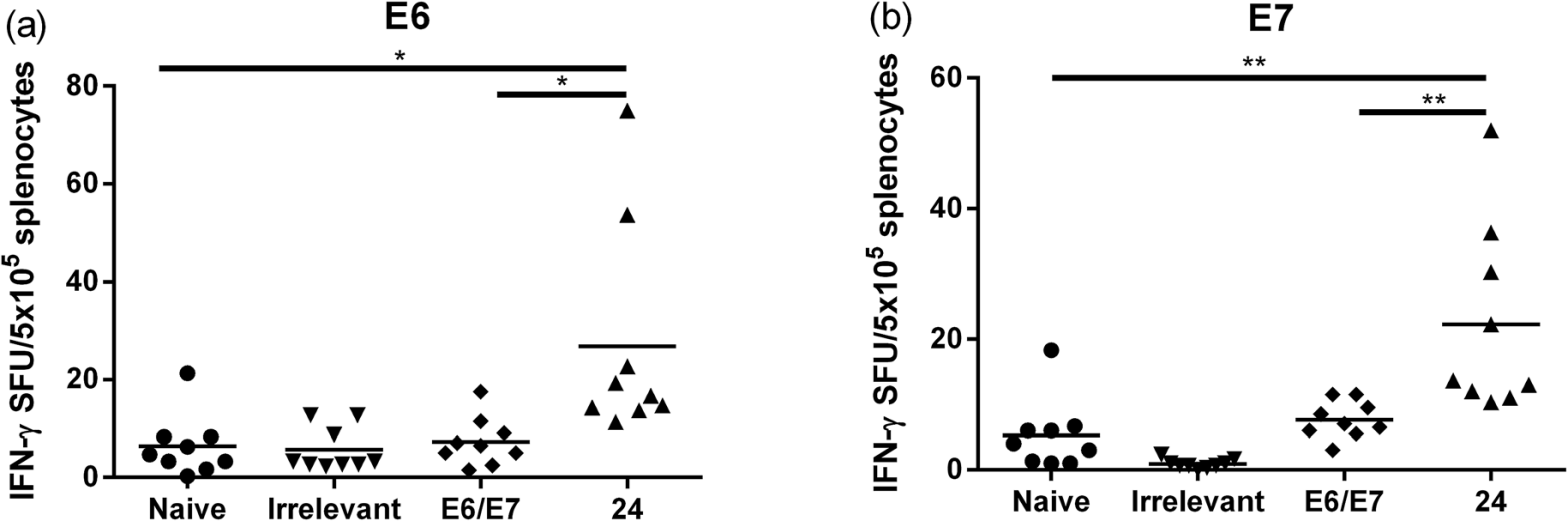
Assessment of CD8^+^ T-cell response to vaccination. Mice were immunised with lipopeptide **24**, a mixture of E6_43–57_ and 8Q_min_ (E6/E7), an “irrelevant” lipopeptide (KQAEDKVKASREAKKQVEKALEQLEDKVK – conjugated with lipid **1**), or PBS subcutaneously in both flanks. Ten days later, spleens were harvested and IFN-γ production in response to (a) short E6 (YDFAFRDL) or (b) short E7 (RAHYNIVTF) peptides was determined by ELISPOT (*n* = 9 mice/group). The data were pooled from two independent experiments and analysed using the unpaired *t* test (*p < 0.05; ***p < 0.001).

Female C57BL/6 (6–8 weeks old) mice (5 + 8/group) were immunized with either lipopeptide **24** (100 μg/100 μL sterile PBS), 30 μg of a mixture of 8Q_min_ and E6_43–57_ emulsified in a total volume of 100 μL of incomplete Freund's adjuvant (Montanide ISA51)/PBS (1 : 1, v/v) as a positive control. The Kaplan–Meier survival curve (Fig. 5b) showed that 46% (6 out of 13 mice) of mice treated with lipopeptide **24** survived over 60 days. Vaccination with lipopeptide **24** induced significantly better survival in tumour-bearing mice than treatment with the PBS as a negative control (*p* = 0.0002) (Fig. 5b). As shown in Fig. 6b, tumour-bearing mice treated with lipopeptide **24** showed slow tumour growth. It is particularly noteworthy that 46% of mice (6 out of 13 mice) treated with lipopeptide **24** were tumour free after 60 days (Fig. 6c). The therapeutic efficacy of **24** was similar to that of positive control (a mixture of 8Q_min_ + E6_43–57_ + ISA51) (Fig. 5b and 6b–d). We also demonstrated that both E7_44–57_ and E6_43–57_ peptides were active in lipopeptide **24** by assessing recall IFN-γ production by CD8 T-cells from immunised mice in response to MHC class I-restricted E7 (RAHYNIVTF) or E6 (YDFAFRDL) peptide restimulation by ELISPOT (Fig. 7).

## Conclusions

We established a synthetic double conjugation pathway to develop multiantigenic lipopeptide conjugates as self-adjuvanting therapeutic vaccine candidates to treat HPV-related cancers. The method involved a Michael addition mercapto-acryloyl reaction between two unprotected peptides followed by an azide-alkyne click reaction to give the final lipopeptide products. Three novel lipidic self-adjuvanting moieties were synthesised, conjugated with the multiantigenic branched peptide and were found to stimulate significantly better survival in an *in vivo* murine HPV model than mice treated with the Pam2Cys analogue **29**, without an external adjuvant and after only a single immunization.

Our double conjugation strategy provided an overall yield 50–80%; can be applied to a wide variety of synthetic applications; was simple to perform; and can be applied on unprotected peptides. This strategy could also be used to rapidly produce libraries of different vaccine constructs from a small selection of starting components (*e.g.* a variety of epitopes and adjuvants). It is anticipated that this technique will be widely used in the chemical synthesis of branched multiantigenic peptides and self-adjuvanting vaccines.

## Experimental section

### Materials

1,3-Di(hydroxymethyl)-5-(prop-2-ynyloxy)benzene was prepared as reported.^24^ Protected l-amino acids were purchased from Novabiochem (Läufelfingen, Switzerland) and Mimotopes (Melbourne, Australia). *p*MBHA resin was purchased from Peptides International (Kentucky, USA). Rink amide MBHA resin, *N*,*N*′-dimethylformamide (DMF), dichloromethane (DCM), methanol, *N*,*N*′-diisopropylethylamine (DIPEA), piperidine and trifluoroacetic acid (TFA) were obtained from Merck (Darmstadt, Germany). Copper wire was purchased from Aldrich (Castle Hill, Australia). (Dimethylamino)-*N*,*N*-dimethyl(3*H*-[1,2,3]triazolo[4,5-*b*]pyridin-3-yloxy)-methanim-inium hexafluorophosphate (HATU) was purchased from Mimotopes (Melbourne, Australia). HPLC grade acetonitrile was obtained from Labscan (Bangkok, Thailand). All other reagents were obtained at the highest available purity from Sigma-Aldrich (Castle Hill, NSW, Australia). Anhydrous hydrofluoric acid (HF) was supplied by BOC gases (Sydney, NSW, Australia). A Kel-F HF apparatus (Peptide Institute, Osaka, Japan) was used for HF cleavage. ESI-MS was performed using a Perkin-Elmer-Sciex API3000 instrument with Analyst 1.4 software (Applied Biosystems/MDS Sciex, Toronto, Canada). High-resolution electrospray ionization mass spectra measurements were obtained on a Bruker micrOTOF mass spectrometer by direct infusion in MeCN at 3 μL min^–1^ using sodium formate clusters as an internal calibrant. Nuclear magnetic resonance (NMR) spectra were recorded with a Bruker Avance 300, 500 or 600 MHz spectrometer (Bruker Biospin, Germany). Chemical shifts are reported in parts per million (ppm) on a *δ* scale, relative to the solvent peak (CDCl_3_*δ*_H_ 7.24, *δ*_C_ 77.0). Coupling constants (*J*) are reported in hertz (Hz) and peak multiplicities described as singlet (s), doublet (d), doublet of doublet (dd), triplet (t), quartet (q), multiplet (m), or broad (br). Analytical RP-HPLC was performed using Shimadzu (Kyoto, Japan) instrumentation (DGU-20A5, LC-20AB, SIL-20ACHT, SPD-M10AVP) with a 1 mL min^–1^ flow rate and detection at 214 nm and/or evaporative light scattering detector (ELSD). Separation was achieved using a 0–100% linear gradient of solvent B over 40 min with Method A (0.1% TFA/H_2_O as solvent A and 90% MeCN/0.1% TFA/H_2_O as solvent B) and/or Method B (0.1% TFA/H_2_O as solvent A and 90% MeOH/0.1% TFA/H_2_O as solvent B) on either a Vydac analytical C4 column (214TP54; 5 μm, 4.6 mm × 250 mm) or a Vydac analytical C18 column (218TP54; 5 μm, 4.6 mm × 250 mm). Preparative RP-HPLC was performed on Shimadzu (Kyoto, Japan) instrumentation (either LC-20AT, SIL-10A, CBM-20A, SPD-20AV, FRC-10A or LC-20AP × 2, CBM-20A, SPD-20A, FRC-10A) in linear gradient mode using a 5–20 mL min^–1^ flow rate, with detection at 230 nm. Separations were performed with solvent A and solvent B on a Vydac preparative C18 column (218TP1022; 10 μm, 22 mm × 250 mm), Vydac semi-preparative C18 column (218TP510; 5 μm, 10 mm × 250 mm) or Vydac semi-preparative C4 column (214TP510; 5 μm, 10 mm × 250 mm). Flash chromatography was performed on Merck Kieselgel 60 as described by Still.^25^ Particle size was measured by dynamic light scattering (DLS) using a Malvern Zetasizer Nano Series with DTS software. Sizes were analysed using a non-invasive backscatter system. Multiplicate measurements were performed at 25 °C with scattering angle of 173° using disposable cuvettes and the number-average hydrodynamic particle diameters are reported.

#### Synthesis of 10-butoxydecan-1-ol (**6**)

Alcohol **6** was synthesised following a reported procedure.^26^ Sodium hydride (60% dispersion in oil, 400 mg, 10.00 mmol, 2 equiv.) was added to a solution of 1,10-decanediol (**4**) (1.74 g, 10.00 mmol, 2 equiv.) in dry DMF (30 mL) and the mixture was stirred for 10 min under a nitrogen atmosphere. A mixture of tetrabutylammonium iodide (TBAI) (93 mg, 0.25 mmol, 0.05 equiv.) and butyl bromide (537 μL, 0.69 g, 5 mmol, 1 equiv.) was added and the mixture was sonicated for 3 h. The reaction mixture was evaporated *in vacuo* and the residue was taken up with a mixture of 5% HCl (100 mL) and ethyl acetate (EtOAc) (100 mL). The aqueous layer was further extracted with EtOAc (2 × 100 mL). The combined organic layers were washed with 0.2 M Na_2_S_2_O_3_ (1 × 50 mL), water (1 × 50 mL), dried over anhydrous MgSO_4_, filtered and evaporated *in vacuo* to afford the crude product as yellow oil. The crude product was purified by silica flash column chromatography (a gradient elution of 0–10% EtOAc in hexane) to afford the butoxydecanol (**6**) as a colourless oil (487 mg, 42%), *R*_f_: 0.20 (10% EtOAc in hexane, KMnO_4_ dip). ^1^H NMR (500 MHz, CDCl_3_) *δ* 3.59 (t, *J* 6.7 Hz, 2H), 3.37 (t, *J* 6.7 Hz, 2H), 3.35 (t, *J* 6.7 Hz, 2H), 1.58 (s, OH, 1H), 1.55–1.49 (m, 6H), 1.37–1.22 (m, 14H), 0.88 (t, *J* 7.4 Hz, 3H). ^13^C NMR (125 MHz, CDCl_3_) *δ* 70.9, 70.6, 63.0, 32.8, 31.8, 29.7, 29.49, 29.48, 29.43, 29.37, 26.1, 25.7, 19.3, 13.9; ESI-MS, *m*/*z*: 231 [M + H]^+^. HRMS calculated for C_14_H_30_NaO_2_^+^ 253.2138, found 253.2130.

#### Synthesis of 6-(octyloxy)hexan-1-ol (**7**)

Alcohol **7** was synthesised following a reported procedure.^26^ Sodium hydride (60% dispersion in oil, 400 mg, 10.0 mmol, 2 equiv.) was added to a solution of diol **5** (1.2 g, 10.00 mmol, 2 equiv.) in dry DMF (30 mL) and the mixture was stirred for 10 min under a nitrogen atmosphere. A mixture of TBAI (93 mg, 0.25 mmol, 0.05 equiv.) and *n*-octyl bromide (864 μL, 0.97 g, 5 mmol, 1 equiv.) was added and the mixture was sonicated for 2 h. The reaction mixture was evaporated *in vacuo* and the residue was taken up with a mixture of 5% HCl (100 mL) and ethyl acetate (EtOAc) (100 mL). The aqueous layer was further extracted with EtOAc (2 × 100 mL). The combined organic layers were washed with 0.2 M Na_2_S_2_O_3_ (1 × 50 mL), water (1 × 50 mL), dried over anhydrous MgSO_4_, filtered and evaporated *in vacuo* to afford the crude product. The crude product was purified by silica flash column chromatography (a gradient elution of 0–10% EtOAc in hexane) to afford the alcohol **7** as a colourless oil (504 mg, 44%), *R*_f_: 0.20 (10% EtOAc in hexane, KMnO_4_ dip). ^1^H NMR (500 MHz, CDCl_3_) *δ* 3.60 (t, *J* 6.6 Hz, 2H), 3.37 (t, *J* 6.6 Hz, 2H), 3.36 (t, *J* 6.8 Hz, 2H), 1.58–1.50 (m, 6H), 1.34 (quintet, *J* 3.7 Hz, 4H), 1.29–1.24 (m, 10H), 0.84 (t, *J* 7.0 Hz, 3H). ^13^C NMR (125 MHz, CDCl_3_) *δ* 71.0, 70.8, 62.8, 32.7, 31.8, 29.71, 29.68, 29.4, 29.2, 26.2, 26.0, 25.6, 22.6, 14.1; ESI-MS, *m*/*z*: 253 [M + Na]^+^. HRMS calculated for C_14_H_30_NaO_2_^+^ 253.2138, found 253.2138.

#### Synthesis of 5,16,20,32-tetraoxahexatriacontan-18-ol (**9**)

Alcohol **9** was synthesised following a reported procedure.^26^ A mix of butoxydecanol (**6**) (165 mg, 0.72 mmol, 2 equiv.), tetrabutylammonium bromide (TBABr) (12 mg, 0.036 mmol, 0.1 equiv.) and NaOH (32 mg, 0.79 mmol, 2.2 equiv.) was stirred for 10 min at room temperature. Epichlorohydrin (28 μL, 33 mg, 0.36 mmol, 1 equiv.) was added and the mixture was stirred at 30 °C for 14 h and then sonicated for 5 h. The reaction mixture was taken up with a mixture of 5% HCl (75 mL) and Et_2_O (50 mL). The aqueous layer was further extracted with Et_2_O (2 × 50 mL). The combined organic layers were washed with water (1 × 50 mL), dried over anhydrous MgSO_4_, filtered and evaporated *in vacuo* to afford the crude product of diglyceride alcohol **9** as a yellow oil. The crude product was taken on to the next reaction without further purification or characterization. *R*_f_: 0.77 (20% EtOAc in DCM, Ce(SO_4_)_2_ dip).

#### Synthesis of 9,16,20,27-tetraoxapentatriacontan-18-ol (**10**)

A mix of alcohol **7** (230 mg, 1.00 mmol, 3 equiv.), TBABr (11 mg, 0.03 mmol, 0.1 equiv.) and NaOH (44 mg, 1.10 mmol, 3.3 equiv.) was stirred for 10 min. Epichlorohydrin (26 μL, 31 mg, 0.33 mmol, 1 equiv.) was added and the mixture was stirred at 30 °C for 14 h and then sonicated for 5 h. The reaction mixture was taken up with a mixture of 5% HCl (75 mL) and Et_2_O (50 mL). The aqueous layer was further extracted with Et_2_O (2 × 50 mL). The combined organic layers were washed with water (1 × 50 mL), dried over anhydrous MgSO_4_, filtered and evaporated *in vacuo* to afford the crude product. The crude product was purified by silica flash column chromatography (a gradient elution of 0–20% EtOAc in DCM) to afford the branched alcohol **10** (74 mg, 43%), *R*_f_: 0.50 (15% EtOAc in DCM, Ce(SO_4_)_2_ dip). ^1^H NMR (400 MHz, CDCl_3_) *δ* 3.93–3.87 (m, 1H), 3.46–3.37 (m, 8H), 3.354 (t, *J* 6.6 Hz, 4H), 3.349 (t, *J* 6.8 Hz, 4H), 2.29 (br s, 1H), 1.57–1.49 (m, 12H), 1.32 (quintet, *J* 3.7 Hz, 8H), 1.28–1.24 (m, 20H), 0.84 (t, *J* 6.9 Hz, 6H). ^13^C NMR (100 MHz, CDCl_3_) *δ* 71.8, 71.5, 71.0, 70.8, 69.4, 31.8, 29.72, 29.68, 29.5, 29.4, 29.2, 26.2, 26.0, 25.9, 22.6, 14.0; ESI-MS, *m*/*z*: 539 [M + Na]^+^. HRMS calculated for C_31_H_64_NaO_5_^+^ 539.4646, found 539.4648.

#### Synthesis of 1,3-bis(hexadecyloxy)propan-2-ol (**11**)

A mix of hexadecane-1-ol (**8**, 485 mg, 2.00 mmol, 3 equiv.), TBABr (22 mg, 0.07 mmol, 0.1 equiv.) and NaOH (88 mg, 2.20 mmol, 3.3 equiv.) was stirred for 10 min. Epichlorohydrin (53 μL, 62 mg, 0.67 mmol, 1 equiv.) was added and the mixture was stirred at 60 °C for 14 h and then sonicated for 5 h. The reaction mixture was taken up with a mixture of 5% HCl (75 mL) and Et_2_O (50 mL). The aqueous layer was further extracted with Et_2_O (2 × 50 mL). The combined organic layers were washed with water (1 × 50 mL), dried over anhydrous MgSO_4_, filtered and evaporated *in vacuo* to afford the crude product. The crude product was purified by silica flash column chromatography (a gradient elution of 0–30% EtOAc in hexane) to afford the branched alcohol **11** (302 mg, 83%), *R*_f_: 0.34 (10% EtOAc in hexane, Ce(SO_4_)_2_ dip). ^1^H NMR (400 MHz, CDCl_3_) *δ* 3.95–3.89 (m, 1H), 3.62 (br s, 1H), 3.48–3.39 (m, 7H), 2.46 (d, *J* 4.2 Hz, 1H), 1.55 (quintet, *J* 7.0 Hz, 4H), 1.29–1.20 (m, 52H), 0.86 (t, *J* 6.9 Hz, 6H). ^13^C NMR (100 MHz, CDCl_3_) *δ* 71.8, 71.7, 69.5, 31.9, 29.7, 29.6, 29.5, 29.4, 26.1, 22.7, 14.1; ESI-MS, *m*/*z*: 564 [M + Na]^+^. HRMS calculated for C_35_H_72_NaO_3_^+^ 563.5374, found 563.5373.

#### Synthesis of 18-(prop-2-yn-1-yloxy)-5,16,20,31-tetraoxapentatria contane or lipoalkyne (**1**)

Sodium hydride (60% dispersion in oil, 16 mg, 0.39 mmol, 1.1 equiv.) was added to a solution of the crude diglyceride alcohol **9** (184 mg, 0.36 mmol, 1 equiv.) in dry DMF (5 mL) and the mixture was stirred for 10 min under a nitrogen atmosphere. Propargyl bromide (80% in toluene, 116 μL, 161 mg, 1.08 mmol, 3 equiv.) was added and the mixture was sonicated for 1 h and stirred overnight. Afterwards, the solvent was evaporated *in vacuo* and the residue was taken up with a mixture of water (50 mL) and EtOAc (50 mL). The organic layer was dried over anhydrous MgSO_4_, filtered and evaporated *in vacuo* to afford the crude product as yellow oil. The crude product was purified by silica flash column chromatography (a gradient elution of 0–10% EtOAc in DCM) to afford the lipoalkyne (**1**) as a yellow oil (57 mg, 29%) over two steps, *R*_f_: 0.29 (1% EtOAc in DCM, Ce(SO_4_)_2_ dip). ^1^H NMR (600 MHz, CDCl_3_) *δ* 4.32 (d, *J* 2.4 Hz, 2H), 3.83 (quintet, *J* 5.1 Hz, 1H), 3.51 (dd, *J* 10.4 Hz, *J* 17.0 Hz, 2H), 3.50 (dd, *J* 10.4 Hz, *J* 17.9 Hz, 2H), 3.411 (dd, *J* 6.6 Hz, *J* 11.9 Hz, 2H), 3.411 (dd, *J* 7.7 Hz, *J* 16.2 Hz, 2H), 3.37 (dd, *J* 6.7 Hz, *J* 13.3 Hz, 8H), 2.38 (t, *J* 2.4 Hz, 1H), 1.56–1.50 (m, 12H), 1.38–1.23 (m, 28H), 0.90 (t, *J* 7.5 Hz, 6H). ^13^C NMR (150 MHz, CDCl_3_) *δ* 80.3, 76.6, 74.0, 71.7, 71.0, 70.8, 70.6, 57.6, 31.9, 29.8, 29.68, 29.62, 29.54, 29.49, 29.45, 26.2, 26.1, 19.4, 13.9; ESI-MS, *m*/*z*: 577 [M + Na]^+^. HRMS calculated for C_34_H_66_NaO_5_^+^ 577.4802, found 577.4804.

#### Synthesis of 18-(prop-2-yn-1-yloxy)-9,16,20,27-tetraoxapentatria contane or lipoalkyne (**2**)

A solution of alcohol **10** (36 mg, 0.10 mmol, 1 equiv.) in dry THF (2 mL) was added dropwise to a suspension of NaH (60% dispersion in oil, 12 mg, 0.30 mmol, 3 equiv.) in dry THF (1 mL) over a period of 5 min at 0 °C under a nitrogen atmosphere. The reaction mixture was stirred for 15 min at 0 °C. A solution of propargyl bromide (80% in toluene, 33 μL, 45 mg, 0.30 mmol, 3 equiv.) in dry THF (1 mL) was added to the reaction mixture at 0 °C over 2 min. The mixture was stirred for 14 h. The solvent was evaporated *in vacuo* and the residue was taken up with a mixture of water (50 mL) and Et_2_O (100 mL). The aqueous layer was further extracted with Et_2_O (100 mL). The combined organic layers were dried over anhydrous MgSO_4_, filtered and evaporated *in vacuo* to afford the crude product. The crude product was purified by silica flash column chromatography (a gradient elution of 0–20% EtOAc in hexane) to afford the alkyne derivative **2** (18 mg, 47%), *R*_f_: 0.12 (5% EtOAc in hexane, Ce(SO_4_)_2_ dip). ^1^H NMR (400 MHz, CDCl_3_) *δ* 4.31 (d, *J* 2.4 Hz, 2H), 3.82 (quintet, *J* 5.2 Hz, 1H), 3.53–3.46 (m, 4H), 3.44–3.39 (m, 4H), 3.363 (t, *J* 6.7 Hz, 4H), 3.359 (t, *J* 6.7 Hz, 4H), 2.38 (t, *J* 2.4 Hz, 1H), 1.56–1.50 (m, 12H), 1.33 (quintet, *J* 3.7 Hz, 8H), 1.29–1.25 (m, 20H), 0.86 (t, *J* 6.9 Hz, 6H). ^13^C NMR (100 MHz, CDCl_3_) *δ* 80.3, 76.6, 74.0, 71.6, 71.0, 70.9, 70.8, 57.6, 31.8, 29.8, 29.7, 29.6, 29.5, 29.3, 26.2, 26.1, 26.0, 22.6, 14.1; ESI-MS, *m*/*z*: 577 [M + Na]^+^. HRMS calculated for C_34_H_66_NaO_5_^+^ 577.4802, found 577.4809.

#### Synthesis of 1-(3-(hexadecyloxy)-2-(prop-2-yn-1-yloxy)propoxy)hexadecane or lipoalkyne (**3**)

The branched alcohol **11** (51 mg, 0.10 mmol, 1 equiv.) in dry THF (2 mL) was added dropwise to a suspension of NaH (60% dispersion in oil, 12 mg, 0.30 mmol, 3 equiv.) in dry THF (1 mL) over a period of 5 min at 0 °C under a nitrogen atmosphere. The reaction mixture was stirred for 15 min at 0 °C. A solution of propargyl bromide (80% in toluene, 33 μL, 45 mg, 0.30 mmol, 3 equiv.) in dry THF (1 mL) was added to the reaction mixture at 0 °C over 2 min. The mixture was stirred for 14 h. The solvent was evaporated *in vacuo* and the residue was taken up with a mixture of water (50 mL) and Et_2_O (100 mL). The aqueous layer was further extracted with Et_2_O (100 mL). The combined organic layers were dried over anhydrous MgSO_4_, filtered and evaporated *in vacuo* to afford the crude product. The crude product was purified by silica flash column chromatography (a gradient elution of 0–10% EtOAc in hexane) to afford the alkyne derivative **3** (31 mg, 56%), *R*_f_: 0.36 (5% EtOAc in hexane, Ce(SO_4_)_2_ dip). ^1^H NMR (400 MHz, CDCl_3_) *δ* 4.31 (d, *J* 2.32 Hz, 2H), 3.83 (quintet, *J* 5.2 Hz, 1H), 3.54–3.47 (m, 4H), 3.45–3.39 (m, 4H), 2.37 (t, *J* 2.3 Hz, 1H), 1.54 (quintet, *J* 6.9 Hz, 4H), 1.25–1.20 (m, 52H), 0.86 (t, *J* 6.8 Hz, 6H). ^13^C NMR (100 MHz, CDCl_3_) *δ* 80.3, 76.6, 74.0, 71.7, 70.8, 57.6, 31.9, 29.69, 29.65, 29.6, 29.5, 29.4, 26.1, 22.7, 14.1; ESI-MS, *m*/*z*: 602 [M + Na]^+^. HRMS calculated for C_38_H_74_NaO_3_^+^ 601.5530, found 601.5535.

#### Synthesis of 8Q_min_ peptide

8Q_min_ epitope (QAEPDRAHYNIVTF; E7_44–57_) was synthesised by manual stepwise SPPS on *p*MBHA resin (substitution ratio: 0.45 mmol g^–1^, 0.2 mmol scale, 0.44 g) using HATU/DIPEA Boc-chemistry. Boc-amino acids were preactivated for 1 min prior to their addition to the resin. The activation of amino acids was achieved by dissolving Boc-amino acid (0.84 mmol, 4.2 equiv.), in 0.5 M HATU/DMF solution (1.6 mL, 0.8 mmol, 4.0 equiv.) followed by the addition of DIPEA (0.22 mL, 1.24 mmol, 6.2 equiv.). Coupling cycle consisted of a Boc deprotection step with neat TFA (2 × 1 min, at rt), a 1 min DMF flow-wash, followed by coupling with 4.2 equiv. of preactivated Boc-amino acids (2 × 1 h). For peptides containing His(DNP) residues, the DNP (2,4-dinitrophenyl) group was cleaved by treating the resin with 20% (v/v) β-mercaptoethanol and 10% (v/v) DIPEA in DMF for 2 × 1 h treatments prior to peptide cleavage. Upon completion of synthesis and removal of the dinitrophenyl (DNP) protecting group, the resin was washed with DMF, DCM, and MeOH, then dried (vacuum desiccator). The peptide was cleaved from the resin using HF, with *p*-cresol as a scavenger. The cleaved peptide was precipitated, filtered, and washed thoroughly with ice-cold Et_2_O and dissolved in 50% MeCN/0.1% TFA/H_2_O. After lyophilization, the crude peptide was obtained as an amorphous powder. The product was purified by a preparative RP-HPLC on C18 column with a 15–35% solvent B gradient over 20 min. HPLC analysis (C18 column, Method A): *t*_R_ = 16.7 min, purity > 95%. Yield: 27%. ESI-MS: *m*/*z* 1661.1 (calc 1660.8) [M + H]^+^; 830.8 (calc 830.9) [M + 2H]^2+^; MW 1659.8.

### General procedure of manual stepwise SPPS on rink amide MBHA resin – Fmoc-chemistry

Peptides were synthesised by manual stepwise SPPS on rink amide MBHA resin (substitution ratio: 0.60 mmol g^–1^, 0.2 mmol scale, 0.33 g) using HATU/DIPEA Fmoc-chemistry. Amino acid activation was achieved by dissolving Fmoc-amino acid (0.84 mmol, 4.2 equiv.), in 0.5 M HATU/DMF solution (1.6 mL, 0.8 mmol, 4.0 equiv.) followed by the addition of DIPEA (146 μL, 0.84 mmol, 4.2 equiv.). Coupling cycle consisted of Fmoc deprotection with 20% of piperidine in DMF (twice, 10 and 20 min), a 1 min DMF flow-wash, followed by coupling with 4.2 equiv. of preactivated Fmoc-amino acids (2 × 1 h). Upon completion of synthesis, the resin was washed with DMF, DCM, and MeOH, then dried (vacuum desiccator). The cleavage of model mercapto-azide was carried out by stirring the resin in the solution of TFA (99%)/triisopropylsilane/water (95 : 2.5 : 2.5) for 4 h. The cleaved peptide was precipitated, filtered, and washed with ice-cold Et_2_O. After lyophilization, the crude peptide was obtained as an amorphous powder.

#### Synthesis of E6_43–57_

E6_43–57_ epitope (QLLRREVYDFAFRDL; E6_43–57_) was synthesised following the general manual stepwise SPPS HATU/DIPEA Fmoc-chemistry procedure. The crude product was purified by a preparative RP-HPLC on C-18 column with 25–45% solvent B gradient over 20 min. HPLC analysis (C-18 column, Method A): *t*_R_ = 19.8 min, purity > 95%. Yield: 84%. ESI-MS: *m*/*z* 970.9 (calc 971.1) [M + 2H]^2+^; 647.8 (calc 647.7) [M + 3H]^3+^; MW 1940.2.

#### Synthesis of azidoacetic acid (N_3_CH_2_CO_2_H)

Azidoacetic acid was synthesised using a similar method to the published procedure.^27^ Sodium azide (6.0 g, 92.3 mmol, 3.0 equiv.) was dissolved in H_2_O (10 mL) and bromoacetic acid (4.3 g, 30.8 mmol, 1.0 equiv.) was added. The reaction mixture was covered and protected from light with aluminum foil. The reaction was stirred continuously in an ice bath for 24 h and subsequently acidified with 32% HCl (10 mL). The product was then extracted with Et_2_O (4 × 50 mL), dried over anhydrous MgSO_4_ and the solvent was evaporated under vacuum. The final product was obtained as a colorless oil (2.95 g, 95%) after prolonged evaporation under vacuum to remove organic solvent and the last traces of water. ^1^H NMR (300 MHz, CDCl_3_) *δ* 3.98 (s, 2H), 10.60 (br s, OH, 1H). ^13^C NMR (100 MHz, CDCl_3_) *δ* 173.7, 50.0.

#### Synthesis of N-terminus 8Q_min_ mercapto-azide (**21**)

N-terminus 8Q_min_ mercapto-azide peptide epitope (N_3_CH_2_CO-CQAEPDRAHYNIVTF) was synthesised following the general manual stepwise SPPS HATU/DIPEA Fmoc-chemistry procedure. Fmoc deprotection of Thr, Val, and Ile were performed with 2% of 1,8-diazabicycloundec-7-ene (DBU) in DMF (twice, 5 and 10 min) instead of 20% piperidine in DMF. The attachment of azidoacetic acid (4.2 equiv.) was achieved using HATU (3 equiv.)/DIPEA (4.2 equiv.) at room temperature (2 × 1 h) and the reaction mixture was covered and protected from light with aluminum foil. The crude product was purified by a preparative RP-HPLC on C-18 column with a 20–40% solvent B gradient over 20 min. HPLC analysis (C-18 column, Method A): *t*_R_ = 18.3 min, purity > 95%. Yield: 72%. ESI-MS: *m*/*z* 924.0 (calc 924.0) [M + 2H]^2+^; MW 1846.

#### Synthesis of N-terminal acryloyl E6_43–57_ (**22**)

N-terminal acryloyl E6_43–57_ peptide epitope (CH_2_=CHCO-QLLRREVYDFAFRDL) was synthesised following the general manual stepwise SPPS HATU/DIPEA Fmoc-chemistry procedure. The coupling of acrylic acid (4.2 equiv.) was achieved using HATU (4 equiv.)/DIPEA (4.2 equiv.) at room temperature (2 × 1 h). The crude product was purified by a preparative RP-HPLC on C-18 column with a 35–55% solvent B gradient over 20 min. HPLC analysis (C-18 column, Method A): *t*_R_ = 22.7 min, purity > 95%. Yield: 33%. ESI-MS: *m*/*z* 998.2 (calc 998.1) [M + 2H]^2+^; 665.8 (calc 665.8) [M + 3H]^3+^; MW 1994.3.

### Preparation of guanidine buffer

6 M guanidine, 50 mM sodium phosphate, 20% acetonitrile, 5 mM EDTA, ∼pH 7.3.

#### Synthesis of multiantigenic peptide azide (**23**) through mercapto-acryloyl conjugation

A mixture of the two peptide epitopes acryloyl E6_43–57_ (**22**) (7.2 mg, 3 μmol, 1.0 equiv.) and 8Q_min_ mercapto-azide (**21**) (13.4 mg, 6 μmol, 2 equiv.) was dissolved in a guanidine buffer at ∼pH 7.3. The reaction mixture was incubated at 37 °C for 48 h. The progress of the reaction was monitored by analytical HPLC until the acryloyl E6_43–57_ (**22**) was completely consumed. The reaction mixture was purified using a semi-preparative HPLC on a C-18 column (20–60% solvent B over 60 min). After lyophilization, the pure azide derivative **23** was obtained as an amorphous white powder. The product was detected using analytical HPLC analysis (C-4 column, Method A), *t*_R_ = 21.8 min, purity > 97% and (C18 column, Method A), *t*_R_ = 21.4 min, purity > 95%. Yield: (12.2 mg, 90%). ESI-MS: *m*/*z* 1921.5 (calc 1921.1) [M + 2H]^2+^; 1281.3 (calc 1281.1) [M + 3H]^3+^; 961.2 (calc 961.1) [M + 4H]^4+^; 768.9 (calc 769.1) [M + 5H]^5+^; MW 3840.3.

#### Synthesis of vaccine candidate lipopeptide **24**

A mixture of azide derivative **23** (3.3 mg, 7.5 × 10^–4^ mmol, 1 equiv.) and the lipoalkyne **1** (0.6 mg, 11.3 × 10^–4^ mmol, 1.5 equiv.) was dissolved in DMF (1 mL), and copper wire (80 mg) was added. The air in the reaction mixture was removed by nitrogen bubbling. The reaction mixture was covered and protected from light with aluminum foil and stirred at 50 °C under nitrogen. The progress of the reaction was monitored by analytical HPLC (C-4 column) and ESI-MS until the peptide **23** was completely consumed after 4 h. The reaction mixture was purified using a semi-preparative HPLC on a C-4 column (35–75% solvent B over 60 min). After lyophilization, the pure lipopeptide **24** was obtained as an amorphous white powder. Compound **24** was analysed by HPLC (C-4 column, Method A) *t*_R_ = 29.9 min, purity > 97% (detected by UV at 214 nm) and *t*_R_ = 30.0 min, purity > 96% (detected by evaporative light scattering detector). Yield: (3.2 mg, 87%).

ESI-MS: *m*/*z* 1466.2 (calc 1466.1) [M + 3H]^3+^; 1100.0 (calc 1099.8) [M + 4H]^4+^; 880.2 (calc 880.0) [M + 5H]^5+^; MW 4395.1.

#### Synthesis of vaccine candidate lipopeptide **25**

A mixture of azide derivative **23** (3.0 mg, 0.7 μmol, 1 equiv.) and the lipoalkyne **2** (3.0 mg, 1.8 μmol, 2.5 equiv.) was dissolved in DMF (1 mL), and copper wire (60 mg) was added. The air in the reaction mixture was removed by nitrogen bubbling. The reaction mixture was covered and protected from light with aluminum foil and stirred at 50 °C under nitrogen. The progress of the reaction was monitored by analytical HPLC (C-4 column) and ESI-MS until the peptide **23** was completely consumed after 3 h. The reaction mixture was purified using a semi-preparative HPLC on a C-4 column (40–80% solvent B over 60 min). After lyophilization, the pure lipopeptide **25** was obtained as an amorphous white powder. Compound **25** was analysed by HPLC (C-4 column, Method A) *t*_R_ = 29.9 min and *t*_R_ = 36.2 min (C-4 column, Method B), purity > 97% (detected by UV at 214 nm). Yield: (2.7 mg, 80%).

ESI-MS: *m*/*z* 1466.2 (calc 1466.1) [M + 3H]^3+^; 1100.0 (calc 1099.8) [M + 4H]^4+^; 880.0 (calc 880.0) [M + 5H]^5+^; MW 4395.1.

#### Synthesis of vaccine candidate lipopeptide **26**

A mixture of azide derivative **23** (3.8 mg, 0.9 μmol, 1 equiv.) and the lipoalkyne **3** (2.6 mg, 4.5 μmol, 5 equiv.) was dissolved in a mixture of DMF (0.8 mL) and DMSO (0.5 mL), and copper wire (80 mg) was added. The air in the reaction mixture was removed by nitrogen bubbling. The reaction mixture was covered and protected from light with aluminum foil and stirred at 50 °C under nitrogen. The progress of the reaction was monitored by analytical HPLC (C-4 column) and ESI-MS until the peptide **23** was completely consumed after 7 h. The reaction mixture was purified using a semi-preparative HPLC on a C-4 column (45–85% solvent B over 60 min). After lyophilization, the pure lipopeptide **26** was obtained as an amorphous white powder. Compound **26** was analysed by HPLC (C-4 column, Method A) *t*_R_ = 35.3 min, purity > 97% (detected by UV at 214 nm). Yield: (2.1 mg, 49%). ESI-MS: *m*/*z* 1474.2 (calc 1474.1) [M + 3H]^3+^; 1106.0 (calc 1105.8) [M + 4H]^4+^; 885.0 (calc 884.9) [M + 5H]^5+^; MW 4419.2.

#### Synthesis of N-terminus 8Q_min_-azide

N-terminus 8Q_min_-azide peptide epitope (N_3_CH_2_CO-QAEPDRAHYNIVTF) was synthesised following the general manual stepwise SPPS HATU/DIPEA Fmoc-chemistry procedure. Fmoc deprotection of Thr, Val, and Ile were performed with 2% of 1,8-diazabicycloundec-7-ene (DBU) in DMF (twice, 5 and 10 min) instead of 20% piperidine in DMF. The attachment of azidoacetic acid (4.2 equiv.) was achieved using HATU (3 equiv.)/DIPEA (4.2 equiv.) at room temperature (2 × 1 h) and the reaction mixture was covered and protected from light with aluminum foil. The crude product was purified by a preparative RP-HPLC on C-18 column with a 15–35% solvent B gradient over 20 min. HPLC analysis (C-18 column, Method A): *t*_R_ = 17.9 min, purity > 95%. Yield: 80%. ESI-MS: *m*/*z* 1744.4 (calc 1743.9) [M + H]^+^; 872.2 (calc 872.9) [M + 2H]^2+^; MW 1742.85.

#### Synthesis of N-terminus E6_43–57_-azide

N-terminus E6_43–57_-azide peptide epitope (N_3_CH_2_CO-QLLRREVYDFAFRDL) was synthesised following the general procedure by manual stepwise SPPS HATU/DIPEA Fmoc-chemistry. The attachment of azidoacetic acid (4.2 equiv.) was achieved using HATU (3 equiv.)/DIPEA (4.2 equiv.) at room temperature (2 × 1 h) and the reaction mixture was covered and protected from light with aluminum foil. The crude product was purified by a preparative RP-HPLC on C-18 column with a 35–55% solvent B gradient over 20 min. HPLC analysis (C-18 column, Method A): *t*_R_ = 23.3 min, purity > 95%. Yield: 50%. ESI-MS: *m*/*z* 1012.8 (calc 1012.6) [M + 2H]^2+^; 675.7 (calc 675.4) [M + 3H]^3+^; MW 2023.

#### Synthesis of lipid 1 conjugated with 8Q_min_ (**27**)

A mixture of 8Q_min_-azide (4.2 mg, 2 × 10^–3^ mmol, 1 equiv.) and the lipoalkyne **1** (1.7 mg, 3 × 10^–3^ mmol, 1.5 equiv.) was dissolved in DMF (1 mL), and copper wire (80 mg) was added. The air in the reaction mixture was removed by nitrogen bubbling. The reaction mixture was covered and protected from light with aluminum foil and stirred at 50 °C under nitrogen. The progress of the reaction was monitored by analytical HPLC (C-4 column) and ESI-MS until the 8Q_min_-azide was completely consumed after 3 h. The reaction mixture was purified using a semi-preparative HPLC on a C-4 column (35–75% solvent B over 60 min). After lyophilization, the pure product **27** was obtained as an amorphous white powder. Compound **27** was analysed by HPLC (C-4 column, Method A) *t*_R_ = 30.9 min, purity > 97% (detected by UV at 214 nm) and *t*_R_ = 30.4 min, purity > 96% (detected by evaporative light scattering detector). Yield: (1.7 mg, 32%). ESI-MS: *m*/*z* 1150.1 (calc 1149.9) [M + 2H]^2+^; 767.2 (calc 766.9) [M + 3H]^3+^; MW 2297.7.

#### Synthesis of lipid 1 conjugated with E6_43–57_ (**28**)

A mixture of E6_43–57_-azide (5.0 mg, 2 × 10^–3^ mmol, 1 equiv.) and the lipoalkyne **1** (1.7 mg, 3 × 10^–3^ mmol, 1.5 equiv.) was dissolved in DMF (1 mL), and copper wire (80 mg) was added. The air in the reaction mixture was removed by nitrogen bubbling. The reaction mixture was covered and protected from light with aluminum foil and stirred at 50 °C under nitrogen. The progress of the reaction was monitored by analytical HPLC (C-4 column) and ESI-MS until the E6_43–57_-azide was completely consumed after 6 h. The reaction mixture was purified using a semi-preparative HPLC on a C-4 column (35–75% solvent B over 60 min). After lyophilization, the pure product **28** was obtained as an amorphous white powder. Compound **28** was analysed by HPLC (C-4 column, Method A) *t*_R_ = 27.4 min, purity > 97% (detected by UV at 214 nm). Yield: (3.6 mg, 58%). ESI-MS: *m*/*z* 1289.8 (calc 1290.1) [M + 2H]^2+^; 860.1 (calc 860.4) [M + 3H]^3+^; MW 2578.1.

#### Synthesis of *S*-(2,3-dihydroxypropyl) cysteine (Dhc-OH)^28^

As shown in Scheme 6, a mixture of l-cysteine hydrochloride (1 g, 6 mmol), 3-bromo-propan-1,2-diol (1.4 g, 0.79 mL, 9 mmol) and triethylamine (2 g, 2.7 mL, 19 mmol) in water (5 mL) was covered and protected from light with aluminum foil and kept for 3 days. The reaction mixture was evaporated *in vacuo* and the residue was washed with acetone (3 × 15 mL) and dried to give Dhc-OH as a white solid (0.8 g, 3.5 mmol, 67%).

#### Synthesis of *N*-fluorenylmethoxycarbonyl-*S*-(2,3-dihydroxypropyl)cysteine (Fmoc-Dhc-OH)^28^

As shown in Scheme 6, a solution of fluorenylmethoxycarbonyl-*N*-hydroxysuccinimide (1.15 g, 3.5 mmol) in acetonitrile (10 mL) was added to a solution of Dhc-OH (0.8 g, 3.5 mmol) in 9% sodium carbonate (10 mL). The reaction mixture was stirred at room temperature. After 2 h, water (100 mL) was added and the solution was acidified to pH 2 with concentrated hydrochloric acid and then extracted with ethyl acetate (3 × 100 mL). The combined organic layers were washed with water (2 × 50 mL) and brine (2 × 50 mL), dried over anhydrous MgSO_4_ and evaporated *in vacuo* to give sticky colourless solid. The crude product was purified with a preparative RP-HPLC on C-18 column with a 25–45% solvent B gradient over 20 min. Yield: 23%. ESI-MS: *m*/*z* 418.5 (calc 418.5) [M + H]^+^; 835.6 (calc 836.0) [2M + H]^+^; 1252.8 (calc 1253.4) [3M + H]^+^; MW 417.

#### Synthesis of Pam2Cys-alkyne

Pam2Cys-alkyne was synthesised following the general manual stepwise SPPS HATU/DIPEA Fmoc-chemistry procedure (Scheme 6). The attachment of Fmoc-Dhc-OH was achieved by dissolving a mixture of Fmoc-Dhc-OH (3 equiv.), DIC (3 equiv.) and HOBt (3 equiv.) in DMF (2 mL) at 0 °C for 5 min. The activated species was then added to the resin and left to couple for 4 h, followed by a thorough wash with DMF and DCM. The *S*-glycerol-cysteine hydroxyl groups were then palmitoylated by addition of palmitic acid (20 eq.) activated with DIC (25 eq.) and 4-(dimethylamino)pyridine (DMAP; 2 eq.) in DCM. The reaction was left to complete overnight, and the resin subsequently washed with DCM. The ε-Mtt protecting group on the C-terminal lysine was then removed by using a mixture of 1% TFA and 5% TIPS in DCM (25 × 5 min) followed by washing with DCM and DMF. The Dhc-associated Fmoc group was then removed by using 2.5% (w/v) DBU in DMF (3 × 5 min), followed by washing with DMF, DCM, and drying under vacuum. The crude product was purified by a preparative RP-HPLC on C-4 column with a 40–80% solvent B gradient over 60 min. HPLC analysis (C-4 column, Method A): *t*_R_ = 33.5 min, purity > 95%. Yield: 20%. ESI-MS: *m*/*z* 1351.2 (calc 1351.9) [M + H]^+^; 676.4 (calc 676.5) [M + 2H]^2+^; MW 1350.9.

#### Synthesis of 8Q_min_/E6_43–57_-Pam2Cys (**29**)

A mixture of azide derivative (**23**) (3.9 mg, 8.8 × 10^–4^ mmol, 1 equiv.) and the Pam2Cys-alkyne (1.9 mg, 1.1 × 10^–3^ mmol, 1.2 equiv.) was dissolved in DMF (1 mL), and copper wire (80 mg) was added. The air in the reaction mixture was removed by nitrogen bubbling. The reaction mixture was covered and protected from light with aluminum foil and stirred at 50 °C under nitrogen. The progress of the reaction was monitored by analytical HPLC (C-4 column) and ESI-MS until the peptide **23** was completely consumed after 5 h. The reaction mixture was purified using a semi-preparative HPLC on a C-4 column (40–80% solvent B over 60 min). After lyophilization, the pure 8Q_min_/E6_43–57_-Pam2Cys (**29**) was obtained as an amorphous white powder. Compound **29** was analysed by HPLC (C-4 column, Method A) *t*_R_ = 30.5 min, purity > 97% (detected by UV at 214 nm). Yield: (3.3 mg, 72%). ESI-MS: *m*/*z* 1298.8 (calc 1298.8) [M + 4H]^4+^; 1039.3 (calc 1039.2) [M + 5H]^5+^; 866.5 (calc 866.2) [M + 6H]^6+^; MW 5191.2.

### Model compounds

#### Synthesis of N-terminal model mercapto-azide (**12**)

N-terminal model mercapto-azide (N_3_CH_2_CO-Cys-Gln-Ala-Glu-Pro-Asp-Phe-NH_2_) was synthesised following the general manual stepwise SPPS HATU/DIPEA Fmoc-chemistry procedure. The attachment of azidoacetic acid (4.2 equiv.) was achieved using HATU (3 equiv.)/DIPEA (4.2 equiv.) at room temperature (2 × 1 h) and the reaction mixture was covered and protected from light with aluminum foil. The crude product was purified by a preparative RP-HPLC on C18 column with a 20–40% solvent B gradient over 20 min. HPLC analysis (C18 column, Method A): *t*_R_ = 15.9 min, purity > 95%. Yield: 60%. ESI-MS: *m*/*z* 891.8 (calc 891.9) [M + H]^+^; MW 890.9.

#### Synthesis of N-terminal acryloyl model (**13**)

The N-terminal acryloyl model peptide (CH_2_=CHCO-Gln-Leu-Leu-Arg-Arg-Tyr-NH_2_) was synthesised following the general manual stepwise SPPS HATU/DIPEA Fmoc-chemistry procedure. The coupling of acrylic acid (4.2 equiv.) was achieved using HATU (4 equiv.)/DIPEA (4.2 equiv.) at room temperature (2 × 1 h). The crude product was purified by a preparative RP-HPLC on C18 column with a 20–40% solvent B gradient over 20 min. HPLC analysis (C18 column, Method A): *t*_R_ = 17.7 min, purity > 95%. Yield: 40%. ESI-MS: *m*/*z* 901.9 (calc 902.1) [M + H]^+^; 451.4 (calc 451.5) [M + 2H]^2+^; MW 901.1.

#### Synthesis of model mercapto-acryloyl conjugation product (**14**)

A mixture of N-terminal model mercapto-azide **12** (1.1 mg, 1.2 μmol, 2 equiv.) and N-terminal acryloyl model peptide **13** (0.7 mg, 0.6 μmol, 1.0 equiv.) was dissolved in a guanidine buffer at ∼pH 7.3. The reaction mixture was incubated at 37 °C for 14 h. The progress of the reaction was monitored by analytical HPLC until the N-terminus acryloyl model was completely consumed. The reaction mixture was purified using a semi-preparative HPLC on a C-18 column (10–50% solvent B over 60 min). After lyophilization, the pure azide product **14** was obtained as an amorphous white powder. Analytical HPLC analysis (C-18 column, Method A) *t*_R_ = 18.3 min, purity > 95%. Yield: 1.2 mg, 92%. ESI-MS: *m*/*z* 1793.3 (calc 1793.0) [M + H]^+^; 897.0 (calc 897.0) [M + 2H]^2+^; MW 1792.

#### Copper-catalysed alkyne-azide cycloaddition (CuAAC) reaction – model study for the synthesis of compound **16**

A mixture of model mercapto-acryloyl conjugation product **14** (1 equiv.) and 1,3-di(hydroxymethyl)-5-(prop-2-ynyloxy)benzene (**15**) (1.5 equiv.) was dissolved in DMF (1 mL), and copper wire was added. The air in the reaction mixture was removed by nitrogen bubbling. The reaction mixture was covered and protected from light with aluminum foil and stirred at 50 °C under nitrogen. The progress of the reaction was monitored by analytical HPLC (C-18 column) until the model mercapto-acryloyl conjugate **14** was completely consumed after 1 h. Analytical HPLC analysis (C-18 column, Method A) for the model CuAAC product **16***t*_R_ = 17.5 min. ESI-MS: *m*/*z* 1985.5 (calc 1985.2) [M + H]^+^; 993.0 (calc 993.1) [M + 2H]^2+^; MW 1984.2.

#### Synthesis of N-terminal model mercapto-azide (**17**)

N-terminal model mercapto-azide (N_3_CH_2_CO-Cys-Lys-Gln-Ala-Glu-Asp-Phe-NH_2_) was synthesised following the general manual stepwise SPPS HATU/DIPEA Fmoc-chemistry procedure. The attachment of azidoacetic acid (4.2 equiv.) was achieved using HATU (3 equiv.)/DIPEA (4.2 equiv.) at room temperature (2 × 1 h) and the reaction mixture was covered and protected from light with aluminum foil. The crude product was purified by a preparative RP-HPLC on C18 column with a 15–35% solvent B gradient over 20 min. HPLC analysis (C18 column, Method A): *t*_R_ = 17.6 min, purity > 95%. Yield: 69%. ESI-MS: *m*/*z* 922.6 (calc 922.4) [M + H]^+^; MW 922.0.

#### Synthesis of N-terminal acryloyl model (**18**)

N-terminal acryloyl model (CH_2_=CHCO-Phe-Ala-Ala-Lys-Lys-Cys(Acm)-NH_2_) was synthesised following the general manual stepwise SPPS HATU/DIPEA Fmoc-chemistry procedure. The coupling of acrylic acid (4.2 equiv.) was achieved using HATU (4 equiv.)/DIPEA (4.2 equiv.) at room temperature (2 × 1 h). The crude product was purified by a preparative RP-HPLC on C18 column with a 20–40% solvent B gradient over 20 min. HPLC analysis (C18 column, Method A): *t*_R_ = 14.1 min and (C4 column, Method A): *t*_R_ = 8.4 min, purity > 95%. Yield: 27%. ESI-MS: *m*/*z* 791.5 (calc 791.4) [M + H]^+^; MW 791.

#### One pot double conjugation reaction to synthesise **20**

A mixture of N-terminal model mercapto-azide **17** (2.0 mg, 2.0 μmol, 2 equiv.) and N-terminal acryloyl model **18** (1.0 mg, 1.0 μmol, 1.0 equiv.) was dissolved in a guanidine buffer (1 mL) at ∼pH 7.3. The reaction mixture was incubated at 37 °C for 72 h. The progress of the reaction was monitored by analytical HPLC until the N-terminus model mercapto azide **17** was completely consumed and the model conjugate azide (**19**) was formed. A mixture of 1,3-di(hydroxymethyl)-5-(prop-2-ynyloxy)benzene (**15**) (1.92 mg, 10 μmol, 10 equiv.) in DMF : DMSO (1 : 1) (0.5 mL) and copper wire (60 mg) was added to the reaction mixture. The air in the reaction mixture was removed by nitrogen bubbling. The reaction mixture was stirred at 50 °C under nitrogen. The progress of the reaction was monitored by analytical HPLC (C-18 column) until **19** was completely consumed after 1 h and gave the final product **20**.

ESI-MS of compound **19**: *m*/*z* 1713.2 (calc 1714.0) [M + H]^+^; 857.1 (calc 857.5) [M + 2H]^2+^; 571.7 (calc 572.0) [M + 3H]^3+^; MW 1713.

ESI-MS of compound **20**: *m*/*z* 953.7 (calc 953.6) [M + 2H]^2+^; 636.2 (calc 636.1) [M + 3H]^3+^; MW 1905.

### Particle size measurement

The final constructs of lipopeptides **24–26** and **29** were dissolved in sterile PBS using a vortex until a homogenous solution was obtained. The particle sizes were measured by dynamic light scattering. The measurements were repeated at least five times. All compounds formed particles with rather high polydispersity. Compound **24** and **25** formed submicron particles (**24**: 450–750 nm, PDI = 0.25–0.55 and **25**: 350–550 nm, PDI is 0.20–0.40). Compounds **26** and **29** formed large aggregates (visible to the naked eye) with sizes above the upper detection level of the instrument (>5 μm).

### Biological assay

#### Mice and cell lines

Female C57BL/6 (6–8 weeks old) mice were used in this study and purchased from Animal Resources Centre (Perth, Western Australia). TC-1 cells (murine C57BL/6 lung epithelial cells transformed with HPV-16 E6/E7 and ras oncogenes).^29^ TC-1 cells were cultured and maintained at 37 °C/5% CO_2_ in RPMI 1640 medium (Gibco) supplemented with 10% heat inactivated fetal bovine serum (Gibco) and 1% nonessential amino acid (Sigma-Aldrich). The animal experiments were approved by the University of Queensland Animal Ethics committee (DI/034/11/NHMRC) and (UQDI/327/13/NHMRC) in accordance with National Health and Medical research Council (NHMRC) of Australia guidelines.

#### Tumor challenge experiments

To test the efficacy of lipopeptide **24** conjugate as a therapeutic vaccine against established tumours, groups of C57BL/6 female mice (8/group) were first challenged subcutaneously in the right flank with 1 × 10^5^ per mouse of TC-1 tumour cells. On the third day after tumour challenge, the mice were injected subcutaneously at the tail base with: 100 μg of lipopeptide **24–26** conjugates in a total volume of 100 μL of sterile-filtered PBS; a mixture of lipid **1** conjugated with 8Q_min_ (**27**) and lipid **1** conjugated with E6_43–57_ (**28**) (100 μg/100 μL sterile PBS, 1 : 1); 8Q_min_/E6_43–57_-Pam2Cys (**29**) (100 μg/100 μL sterile PBS) as a positive control; or 100 μL PBS as a negative control. Each mouse received a single immunization only. The size of the tumour was measured by palpation and calipers every two days and reported as the average tumour size across the group of five mice or as tumour size in individual mice.^30^ Tumour volume was calculated using the formula *V* (cm^3^) = 3.14 × [largest diameter × (perpendicular diameter)^2^]/6.30*b* The mice were euthanised when tumour reached 1 cm^3^ or started bleeding to avoid unnecessary suffering.

The second tumor challenge study was executed with **24** and antigens administered with commercial adjuvant, in the similar manner as described above. Two independent experiments were performed with total 13 C57BL/6 mice per group (5 + 8 mice per group) and the list of the immunization compounds was: lipopeptide **24**; 30 μg of a mixture of 8Q_min_ and E6_43–57_ emulsified in a total volume of 100 μL of Montanide ISA51 (Seppic, France)/PBS (1 : 1, v/v) as a positive control; and PBS as a negative control.

### IFN-gamma ELISPOT assays

Splenocytes were harvested from the spleens of naïve and immunised mice and depleted of red-blood cells using Red Blood Cell Lysing Buffer (0.155 M ammonium chloride in 0.01 M Tris–HCl buffer, Sigma). Splenocytes were then resuspended in RPMI (Sigma) supplemented with 20% FCS (Sigma), 100 U mL^–1^ penicillin and 100 μg mL^–1^ streptomycin, and 50 μM β-mercaptoethanol. The cells were plated at 5 × 10^5^ cells per well in triplicate in ELISPOT plates (Millipore Biotec) which had been previously coated with 5 μg mL^–1^ IFN-γ capture mAb (clone 14-7313-85 eBioscience) in PBS at 4 °C overnight and then blocked with RPMI/20% FCS at room temperature for 3 hours. E7 (RAHYNIVTF) and E6 (YDFAFRDL) peptides (10 μg mL^–1^) were added alongside 10 ng mL^–1^ rhIL-2 (R&D systems) to a final volume of 200 μL per well. Plates were incubated for 18 hours at 37 °C, washed, and a biotinylated IFN-γ detection Ab (2 μg mL^–1^; clone R4-6A2; eBioscience) in 1% BSA added at room temperature for 3 hours. After washing, a streptavidin–HRP complex (DakoCytomation) in 1% BSA was added for 1 h at room temperature, plates were washed again, and bound cytokine was visualised with 3-amino-9-ethylcarbozole (Calbiochem). Spots were counted with an ELISPOT reader (Autoimmun Diagnostika). An “irrelevant” lipopeptide (KQAEDKVKASREAKKQVEKALEQLEDKVK – conjugated with lipid **1**) was used as a control in this experiment. Group A streptococcal B-cell epitope (J14) was selected as the irrelevant peptide.^31^

#### Statistical analysis

All data were analysed using GraphPad Prism 5 software. Kaplan–Meier survival curves for tumour treatment experiments were applied. Differences in survival treatments were determined using the log-rank (Mantel–Cox) test, with *p* < 0.05 considered statistically significant.

## Supplementary Material

Supplementary information
