# Supplementary material for: Double conjugation strategy to incorporate lipid adjuvants into multiantigenic vaccines
Source: Chem Sci. 2016 Jan 4;7(3):2308–21. doi: 10.1039/c5sc03859f (PMC5977935; doi:10.1039/c5sc03859f)
Supplement: Supplementary file 1 [file SC-007-C5SC03859F-s001.pdf]

Electronic Supplementary Material (ESI) for Chemical Science.
This journal is © The Royal Society of Chemistry 2016

Double Conjugation Strategy to Incorporate Lipid Adjuvants into Multiantigenic Vaccines

Waleed M. Hussein, Tzu-Yu Liu, Pirashanthini Maruthayanar, Saori Mukaida, Peter M. Moyle, James W Wells, Istvan Toth,* and Mariusz Skwarczynski*

Supporting Information

Structure, ^{1}H and C^{13} NMR for lipid derivatives

Structure of 18-(prop-2-yn-1-yloxy)-5,16,20,31-tetraoxapentatriacontane or lipoalkyne (1)

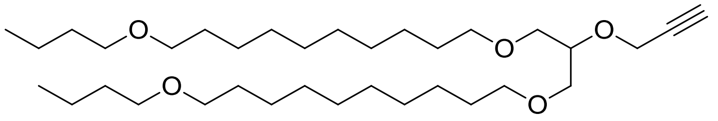

Structure of 18-(prop-2-yn-1-yloxy)-9,16,20,27-tetraoxapentatriacontane or lipoalkyne (2)

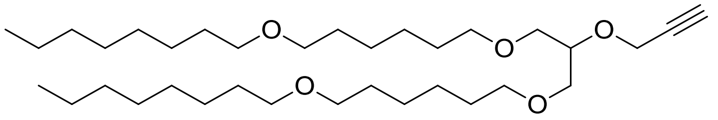

Structure of 1-(3-(hexadecyloxy)-2-(prop-2-yn-1-yloxy)propoxy)hexadecane or lipoalkyne (3)

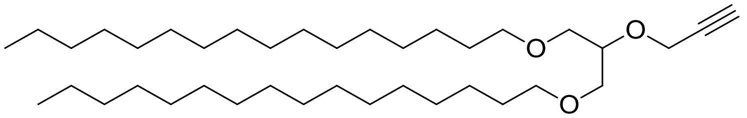

Structure of 10-Butoxydecan-1-ol (6)

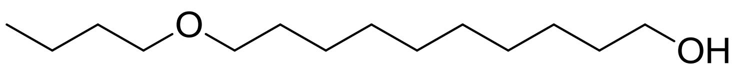

Structure of 6-(octyloxy)hexan-1-ol (7)

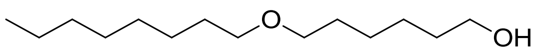

Structure of 5,16,20,32-tetraoxahexatriacontan-18-ol (9)

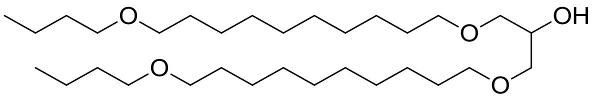

Structure of 9,16,20,27-tetraoxapentatriacontan-18-ol (10)

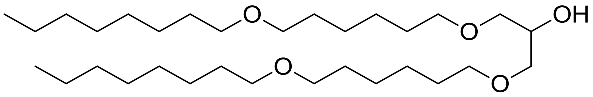

Structure of 1,3-bis(hexadecyloxy)propan-2-ol (11)

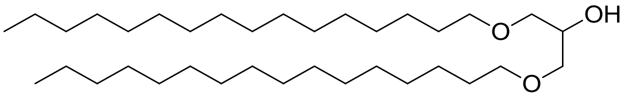

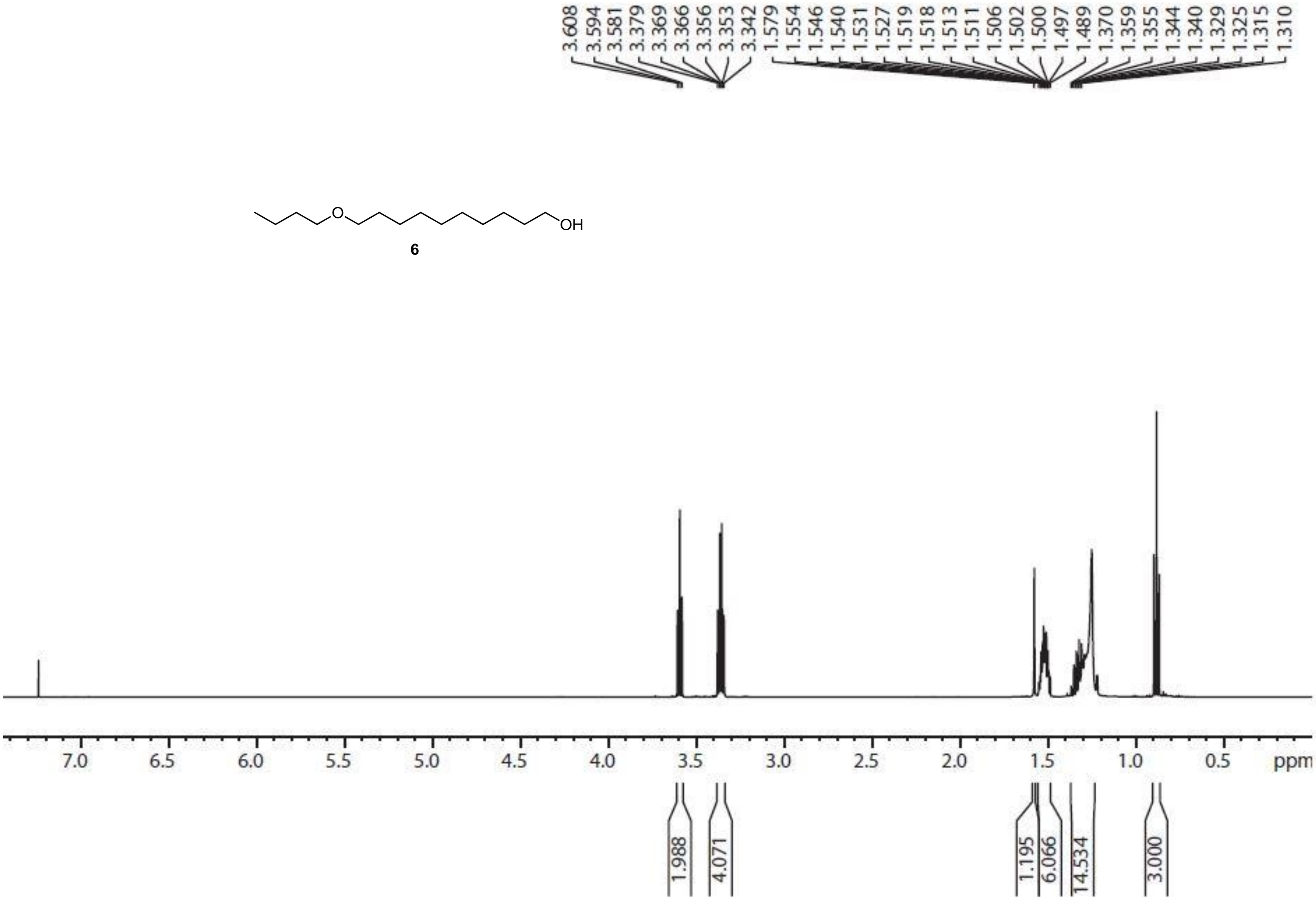
O
OH
6
3.608
3.594
3.581
3.379
3.369
3.366
3.356
3.353
3.342
1.579
1.554
1.546
1.540
1.531
1.527
1.519
1.518
1.513
1.511
1.506
1.502
1.500
1.497
1.489
1.370
1.359
1.355
1.344
1.340
1.329
1.325
1.315
1.310
7.0
6.5
6.0
5.5
5.0
4.5
4.0
3.5
3.0
2.5
2.0
1.5
1.0
0.5
ppm
1.988
4.071
1.195
6.066
14.534
3.000

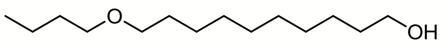

6

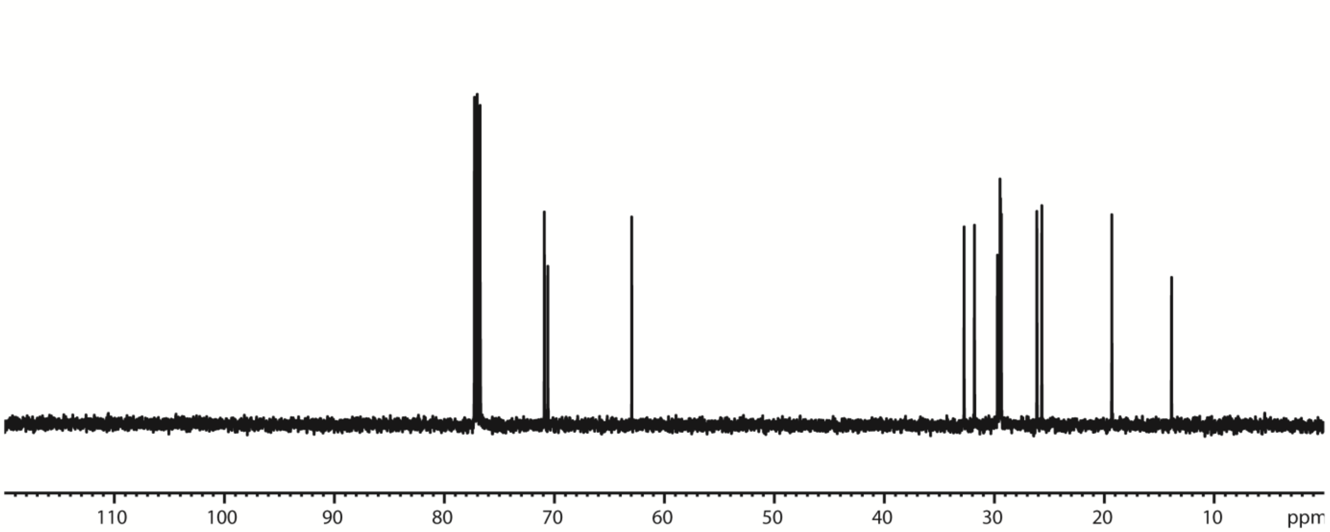
70.922
70.610
62.974
32.750
31.814
29.722
29.493
29.476
29.430
29.367
26.137
25.695
19.329
13.896
110
100
90
80
70
60
50
40
30
20
10
ppm

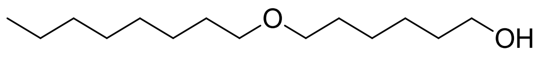

7

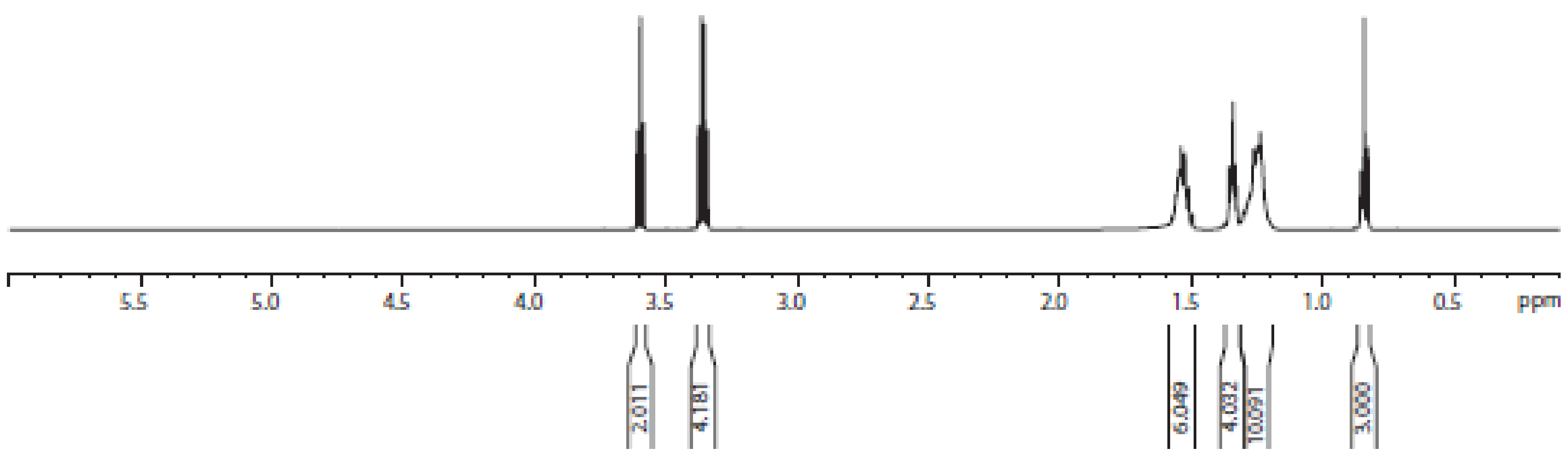

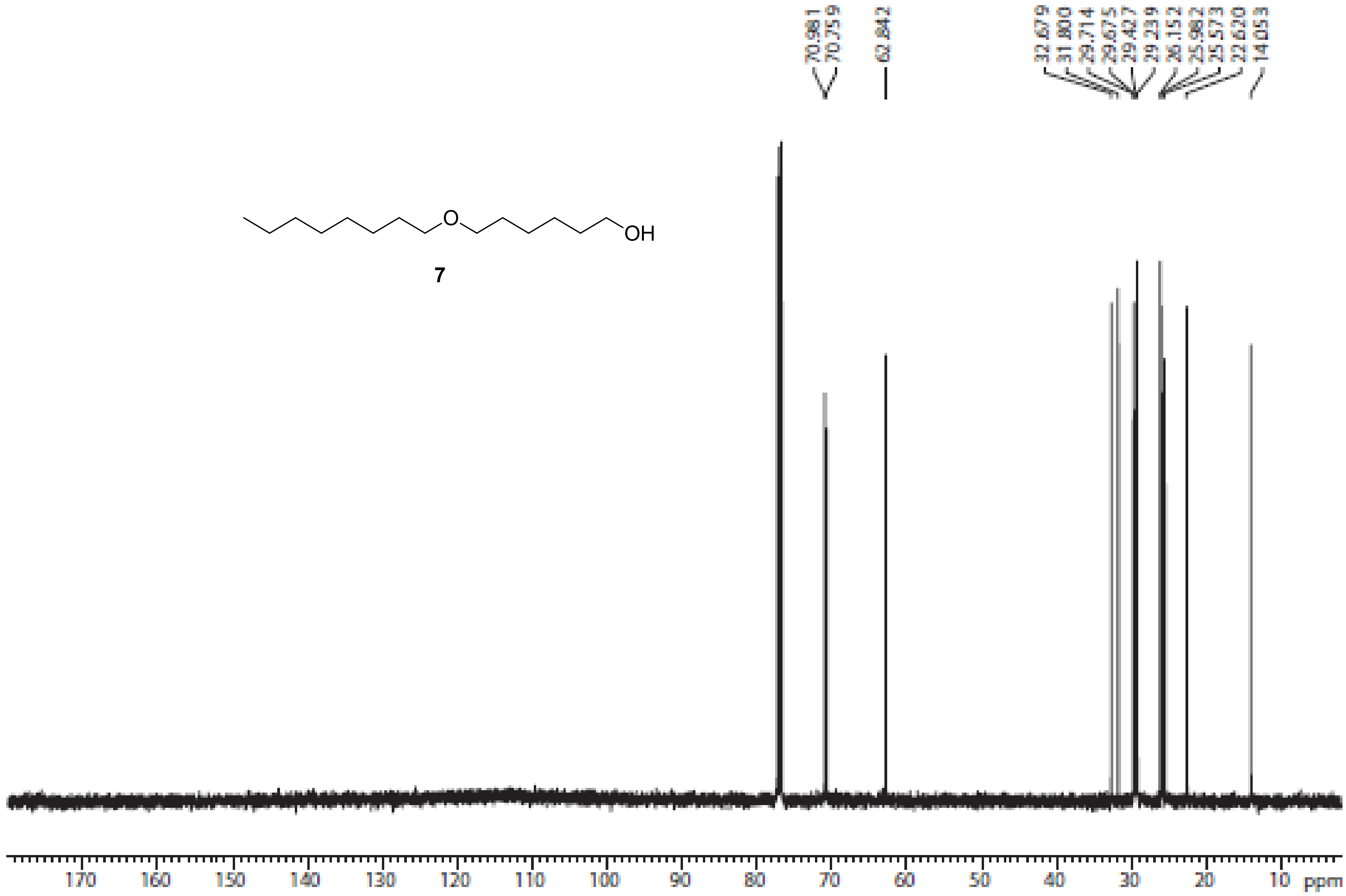
70.981
70.759
62.842
32.679
31.800
29.714
29.675
29.427
29.239
26.152
25.982
25.573
22.620
14.053
O
OH
7
170 160 150 140 130 120 110 100 90 80 70 60 50 40 30 20 10 ppm

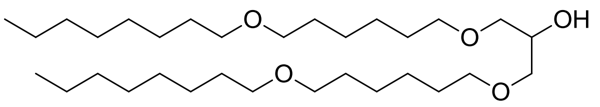

10

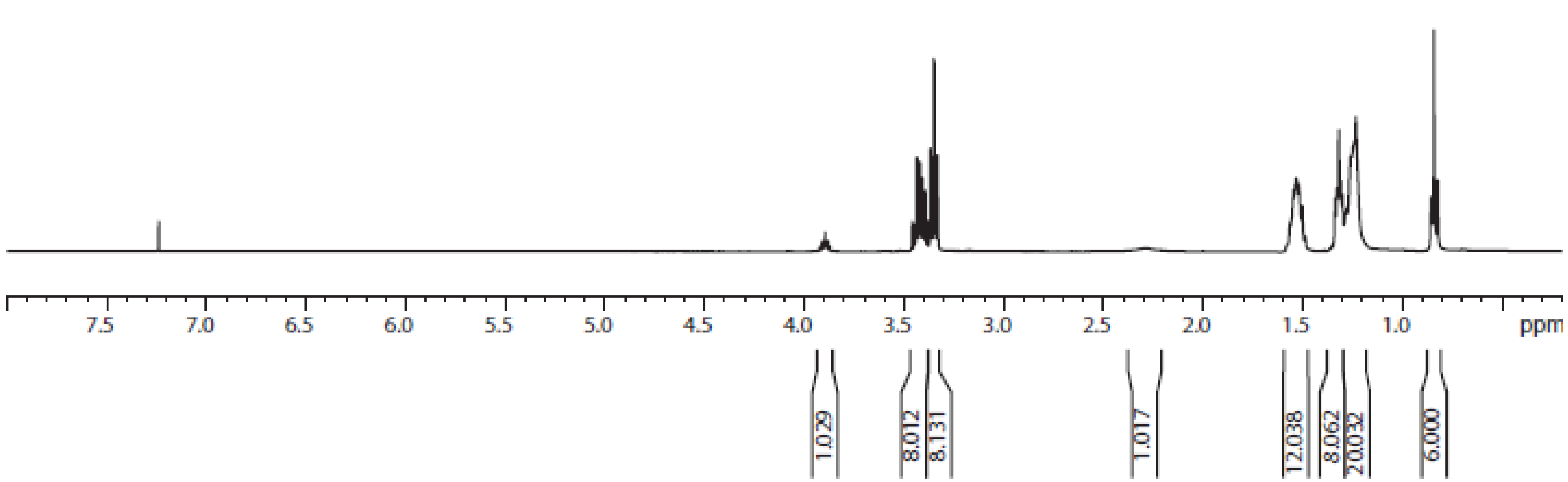

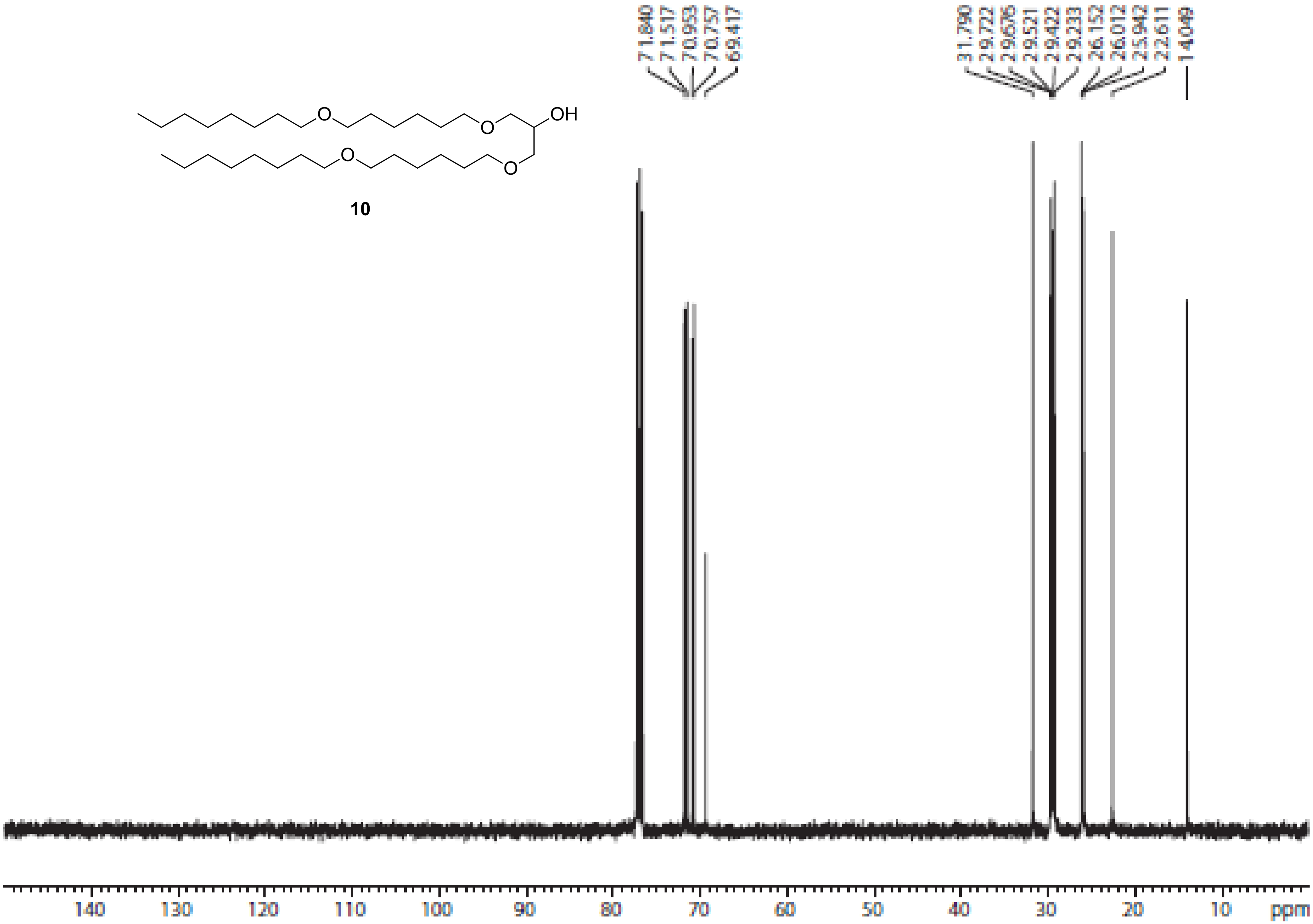
OH
O
O
O
O
71.840
71.517
70.953
70.757
69.417
31.790
29.722
29.676
29.521
29.422
29.233
26.152
26.012
25.942
22.611
14.049
140
130
120
110
100
90
80
70
60
50
40
30
20
10
ppm

10

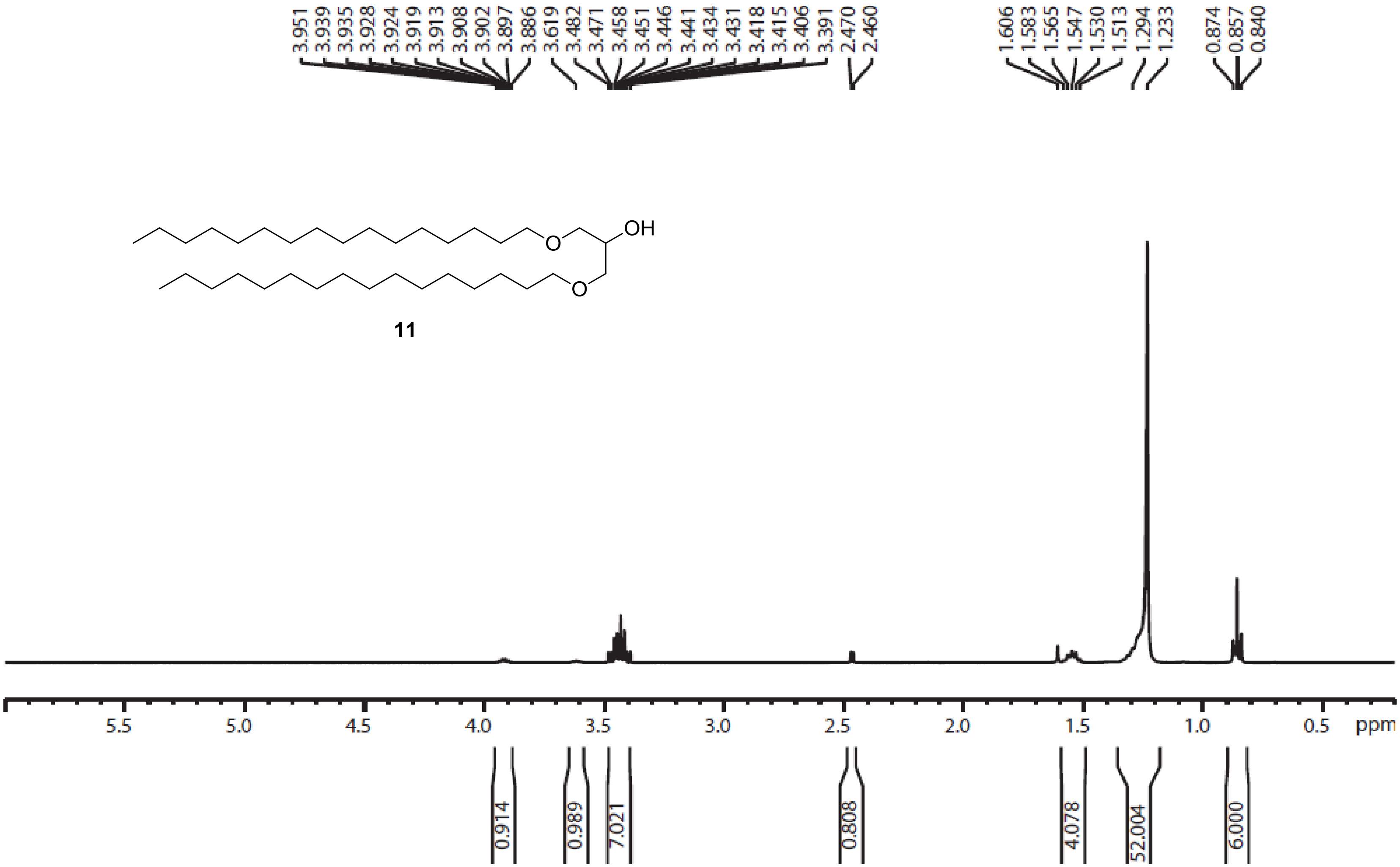
3.951
3.939
3.935
3.928
3.924
3.919
3.913
3.908
3.902
3.897
3.886
3.619
3.482
3.471
3.458
3.451
3.446
3.441
3.434
3.431
3.418
3.415
3.406
3.391
2.470
2.460
1.606
1.583
1.565
1.547
1.530
1.513
1.294
1.233
0.874
0.857
0.840
OH
O
O
5.5
5.0
4.5
4.0
3.5
3.0
2.5
2.0
1.5
1.0
0.5
ppm
0.914
0.989
7.021
0.808
4.078
52.004
6.000

11

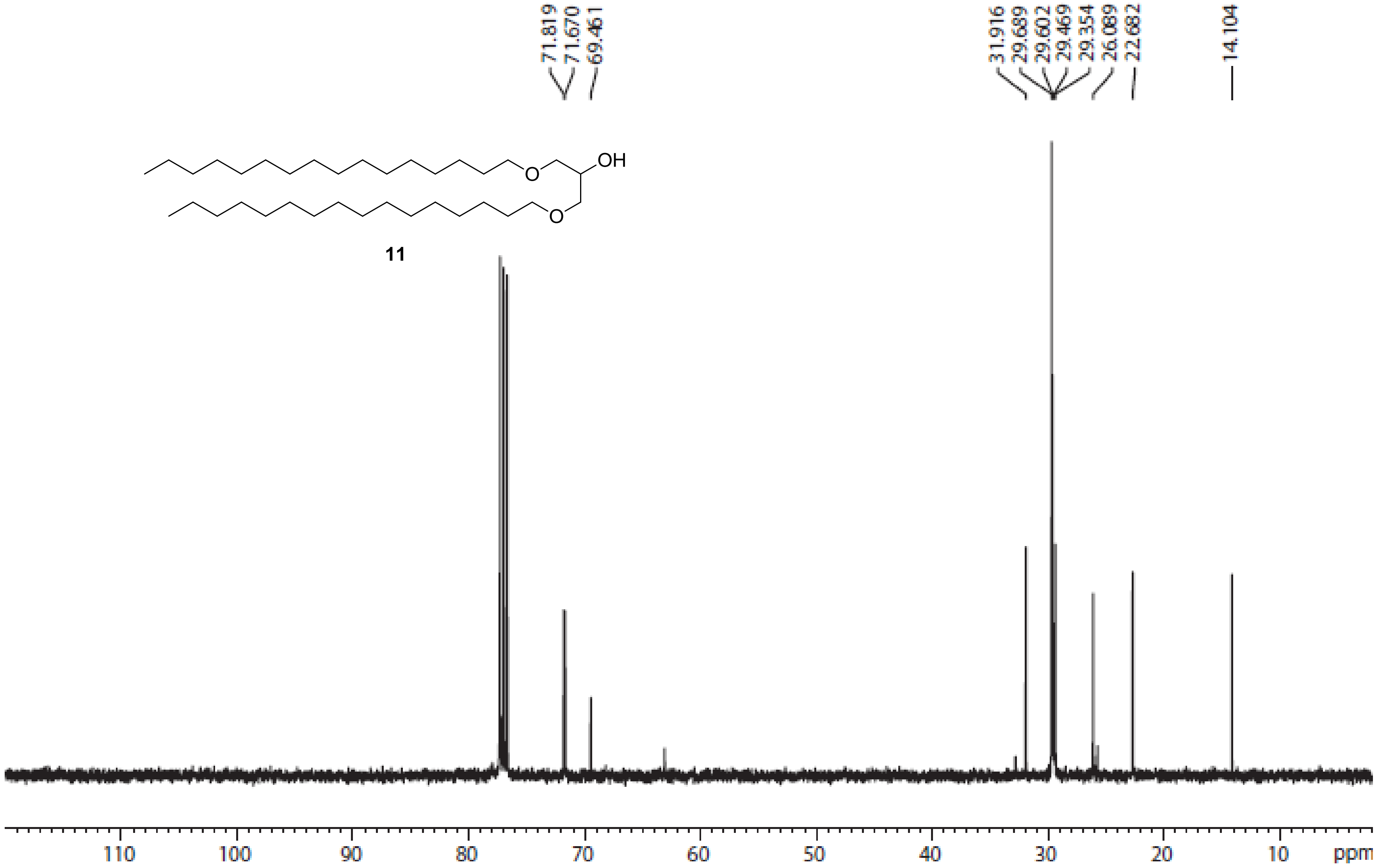
71.819
71.670
69.461
31.916
29.689
29.602
29.469
29.354
26.089
22.682
14.104
OH
O
O
11
110
100
90
80
70
60
50
40
30
20
10
ppm

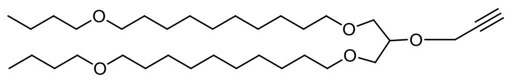

1

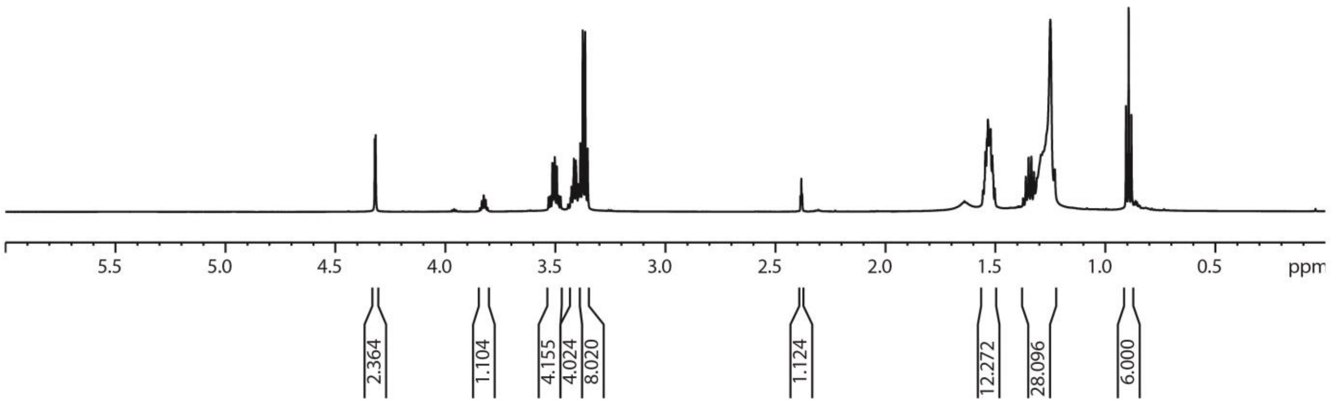
4.321
4.317
3.842
3.833
3.825
3.816
3.808
3.531
3.523
3.514
3.506
3.502
3.493
3.485
3.476
3.442
3.431
3.426
3.415
3.406
3.395
3.391
3.386
3.375
3.364
3.353
2.387
2.383
2.379
1.557
1.546
1.539
1.535
1.528
1.522
1.514
1.503
1.375
1.362
1.350
1.337
1.325
1.251
1.230
0.907
0.895
0.882
5.5
5.0
4.5
4.0
3.5
3.0
2.5
2.0
1.5
1.0
0.5
ppm
2.364
1.104
4.155
4.024
8.020
1.124
12.272
28.096
6.000

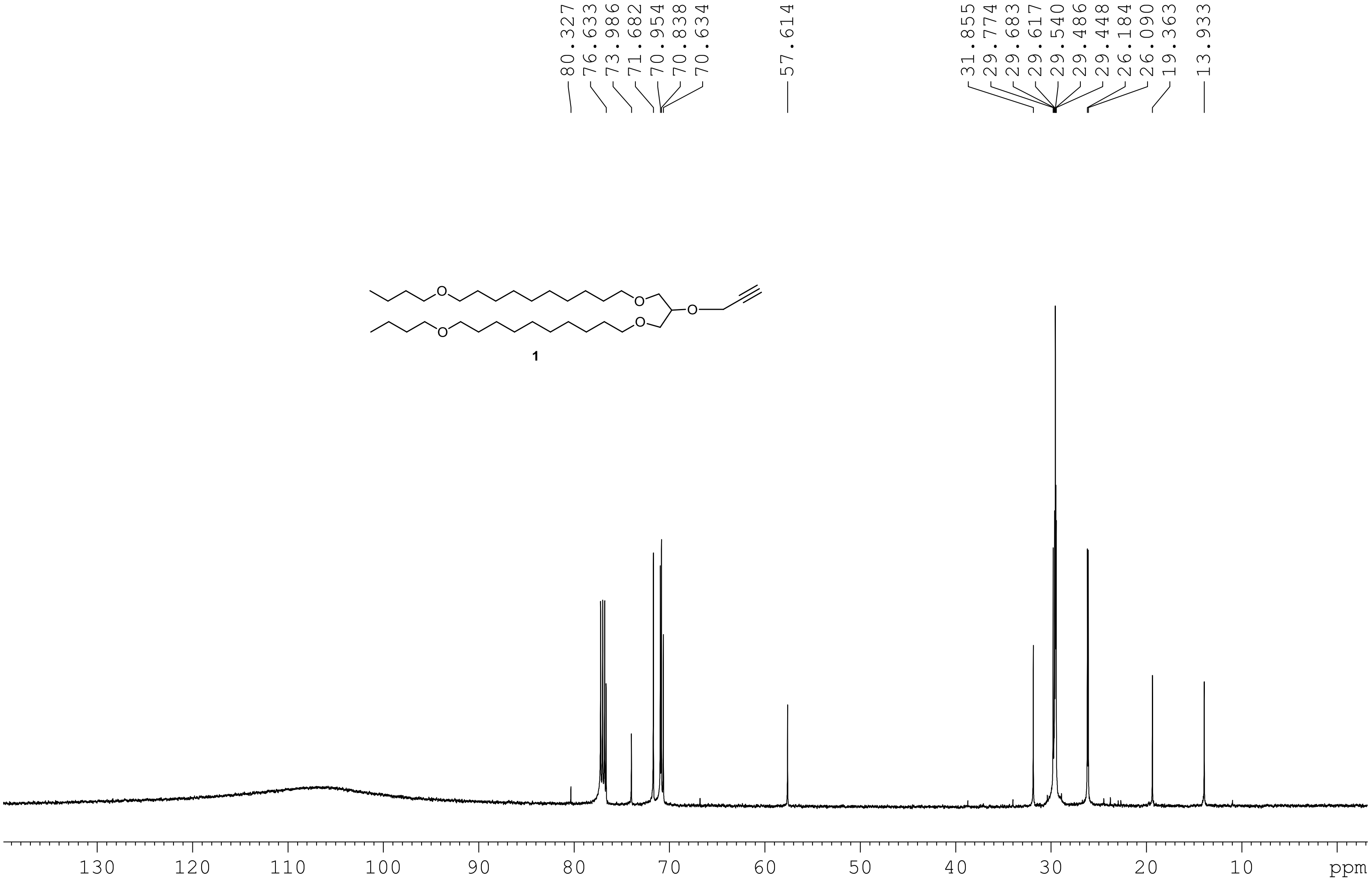
80.327
76.633
73.986
71.682
70.954
70.838
70.634
57.614
31.855
29.774
29.683
29.617
29.540
29.486
29.448
26.184
26.090
19.363
13.933
1
130
120
110
100
90
80
70
60
50
40
30
20
10
ppm

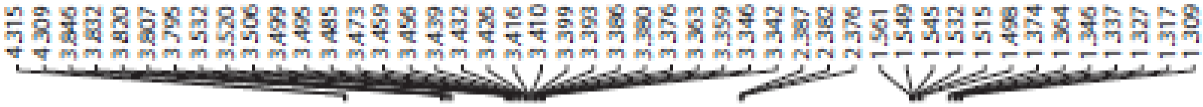

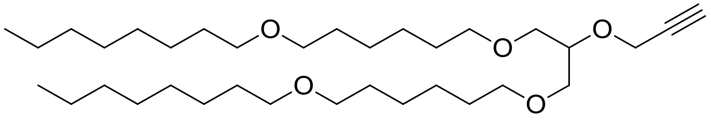

2

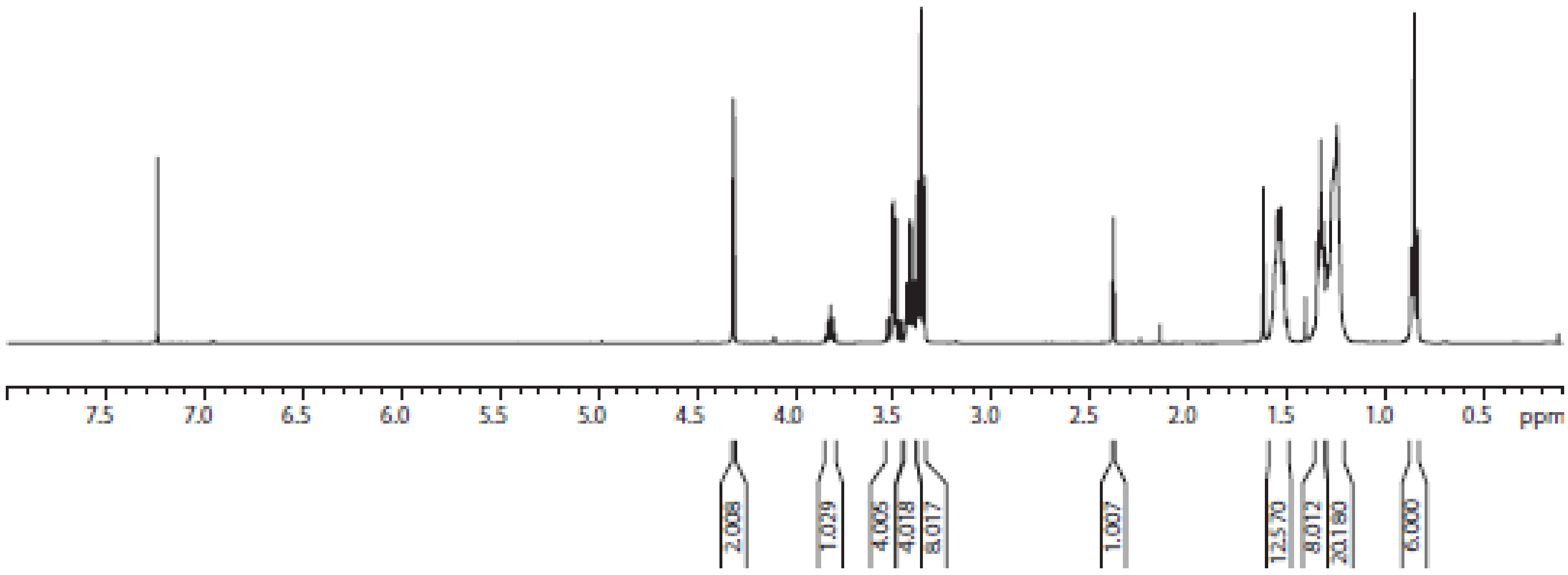

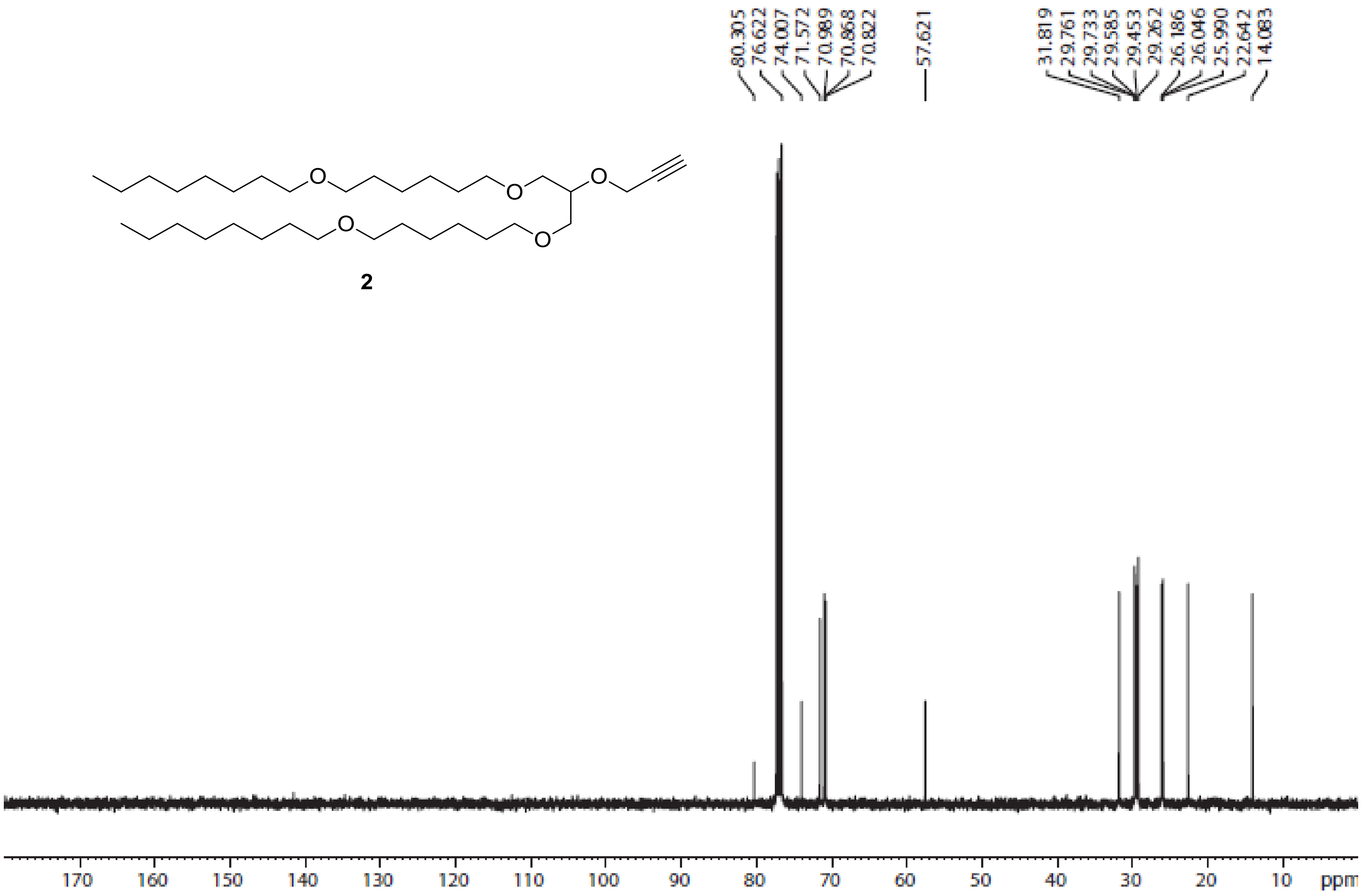

80.305
76.622
74.007
71.572
70.989
70.868
70.822
57.621
31.819
29.761
29.733
29.585
29.453
29.262
26.186
26.046
25.990
22.642
14.083
170
160
150
140
130
120
110
100
90
80
70
60
50
40
30
20
10
ppm

2

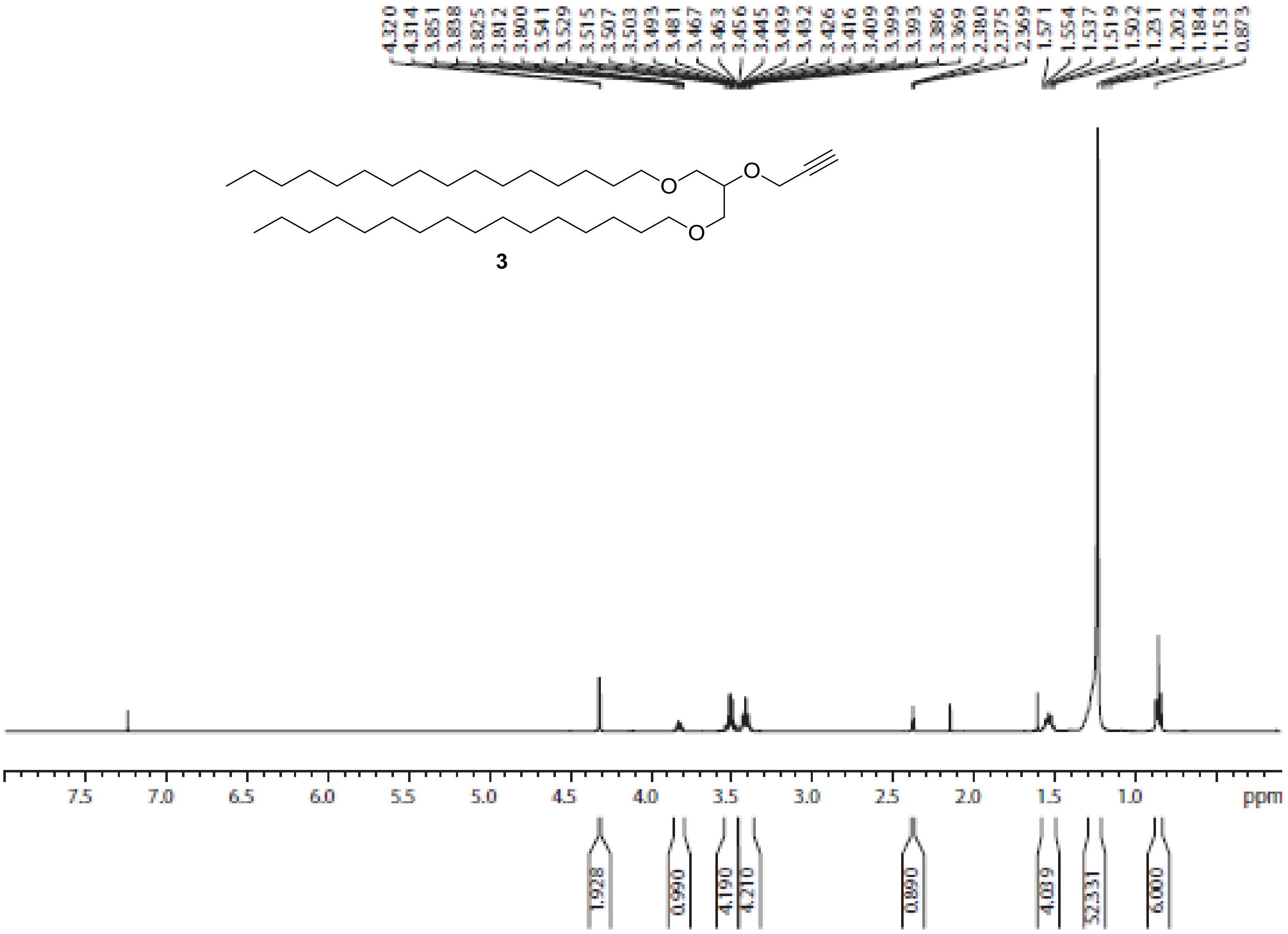

4.320
4.314
3.851
3.838
3.825
3.812
3.800
3.541
3.529
3.515
3.507
3.503
3.493
3.481
3.467
3.463
3.456
3.445
3.439
3.432
3.426
3.416
3.409
3.399
3.393
3.386
3.369
2.380
2.375
2.369
1.571
1.554
1.537
1.519
1.502
1.231
1.202
1.184
1.153
0.873
7.5
7.0
6.5
6.0
5.5
5.0
4.5
4.0
3.5
3.0
2.5
2.0
1.5
1.0
ppm
1.928
0.990
4.190
4.210
0.890
4.039
52.531
6.000

3

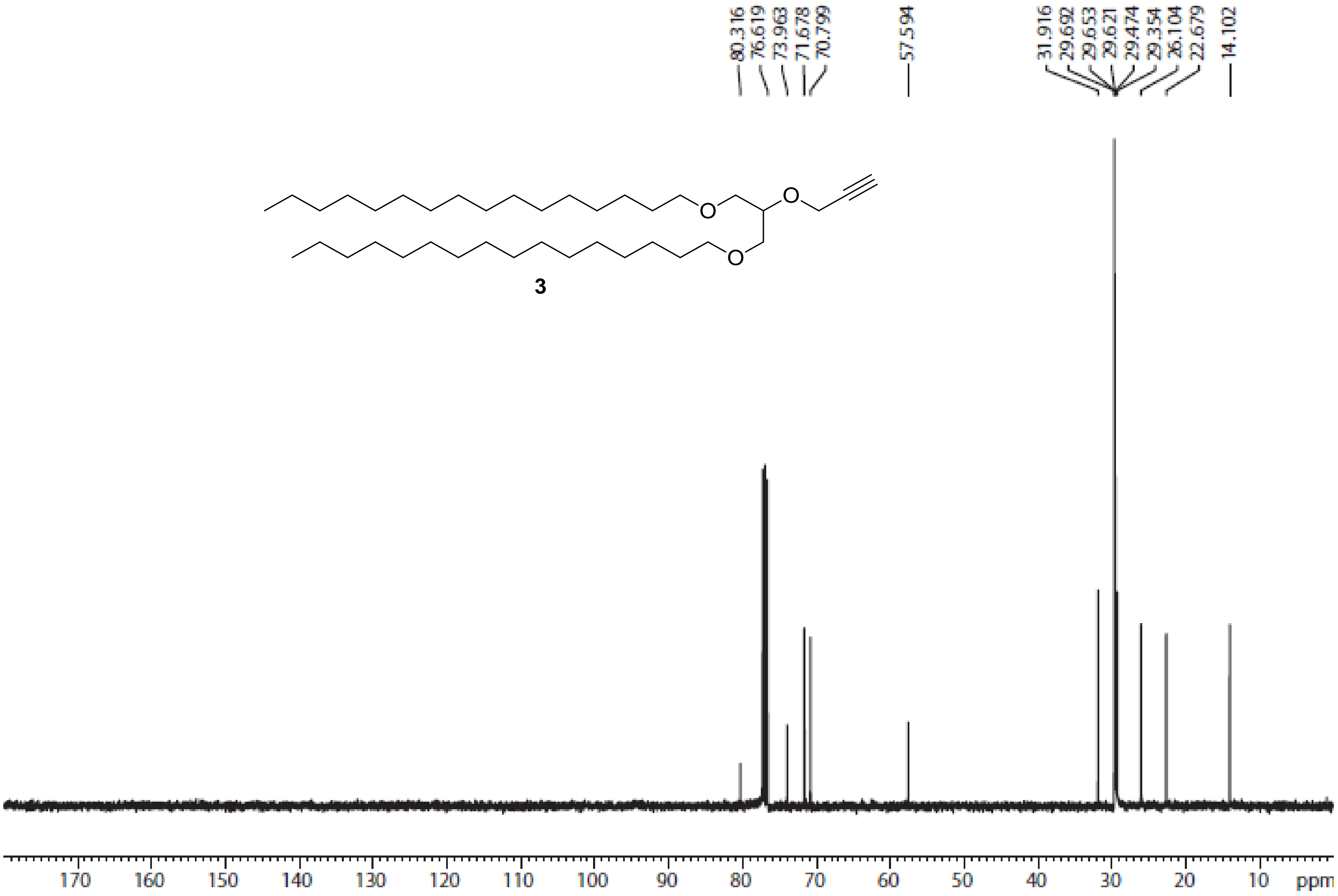
80.316
76.619
73.963
71.678
70.799
57.594
31.916
29.692
29.653
29.621
29.474
29.354
26.104
22.679
14.102
O
O
O
3
170 160 150 140 130 120 110 100 90 80 70 60 50 40 30 20 10 ppm

Structure and HPLC for Known compounds and structure, HPLC and mass spec for novel compounds

Structure of $8Q_{min}$ Peptide.

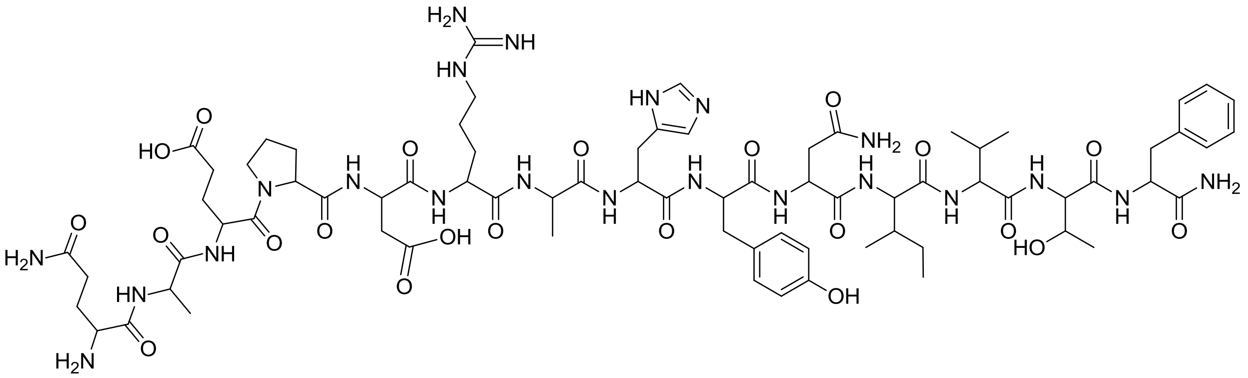

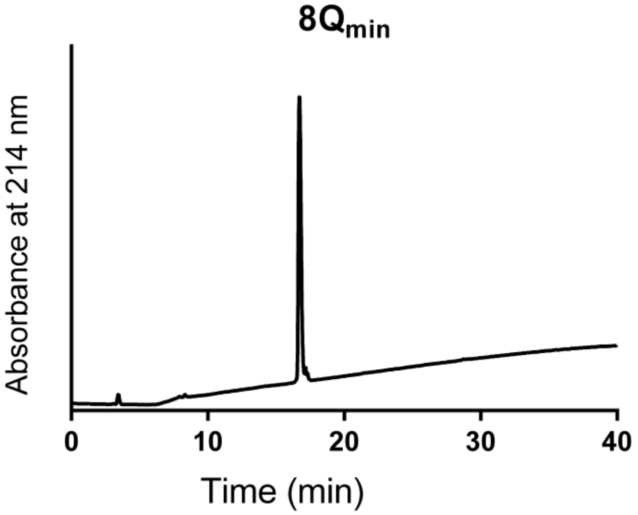

Structure of $E6_{43\text{-}57}$.

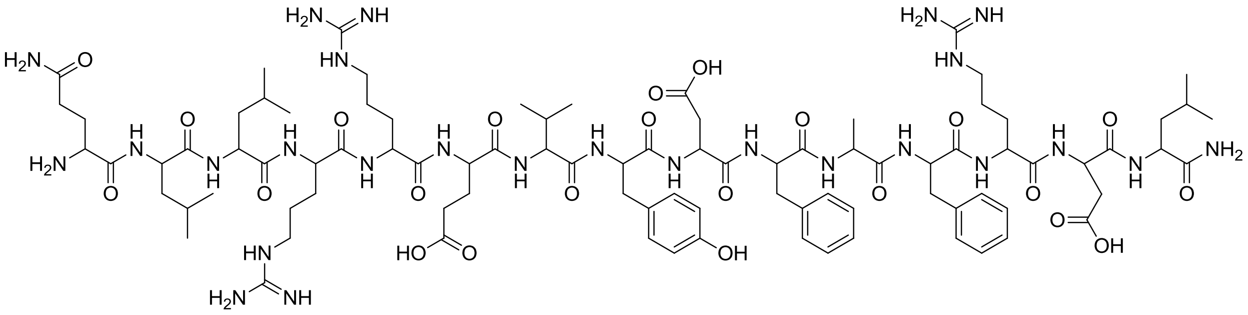

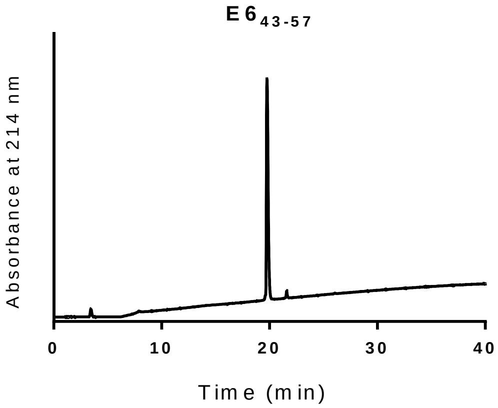

Structure of N-terminus 8Q$_{min}$ mercapto-azide (21).

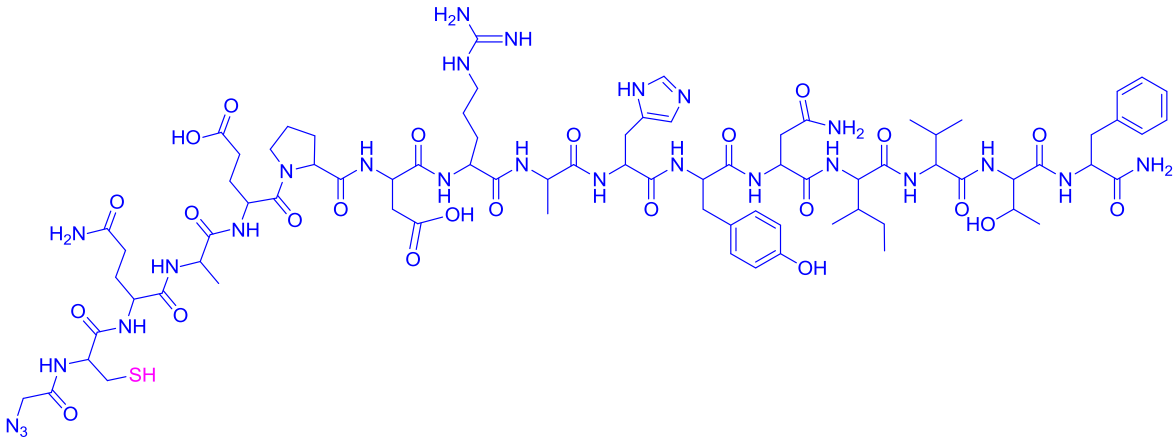

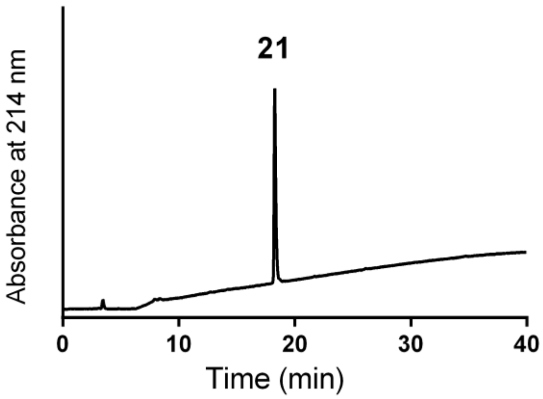

Structure of N-terminal acryloyl E6$_{43\text{-}57}$ (22).

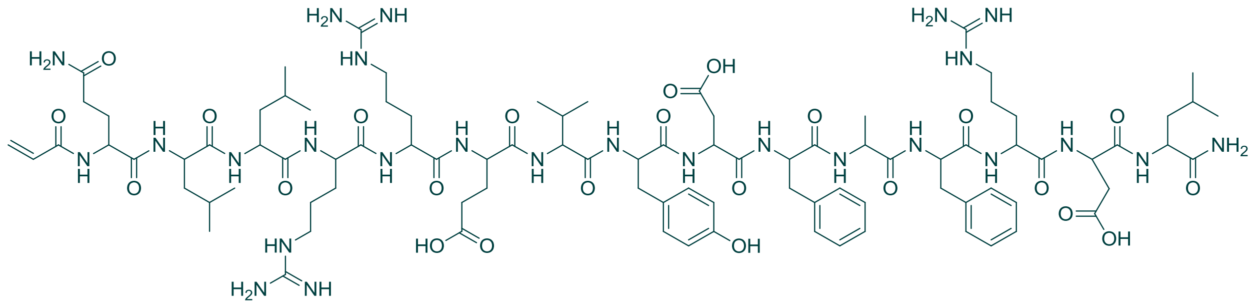

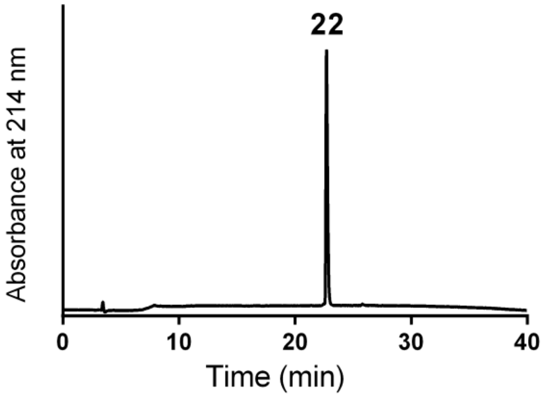

Structure of multiantigenic peptide azide (23)

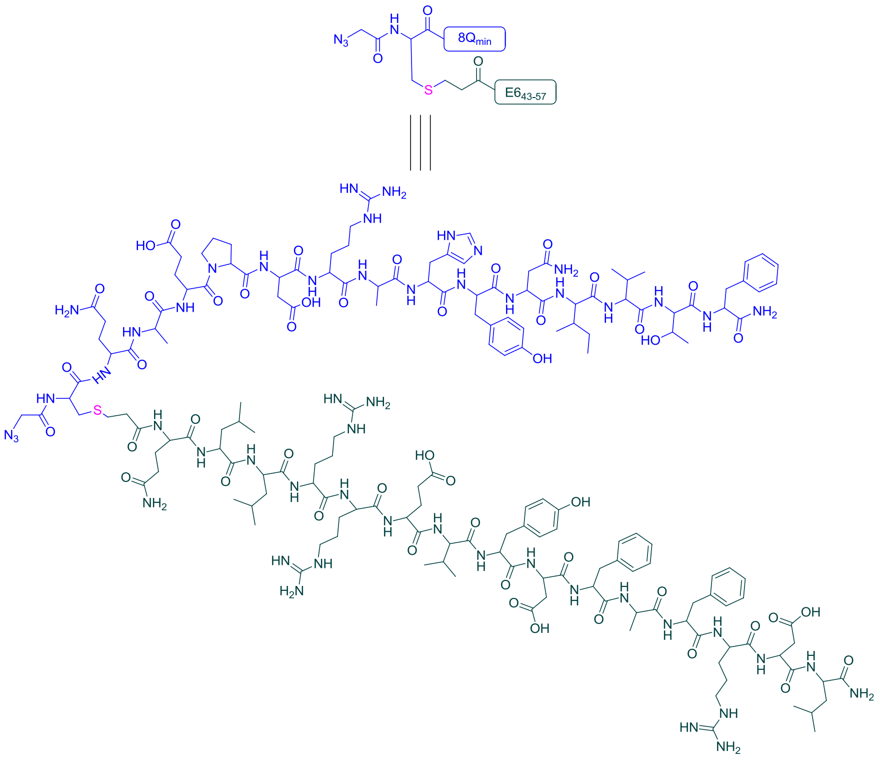

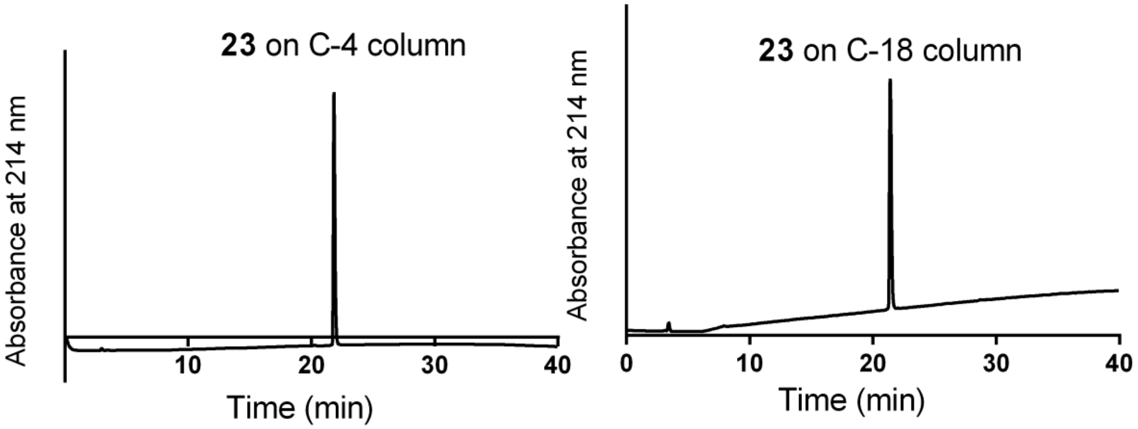

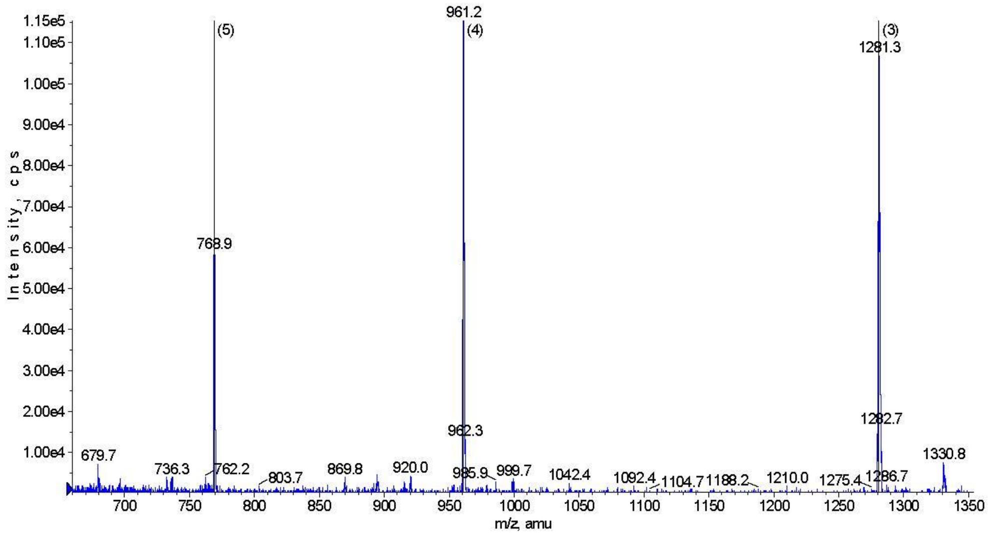

Mass spectrum of azide derivative (**23**)

Structure of vaccine candidate lipopeptide 24

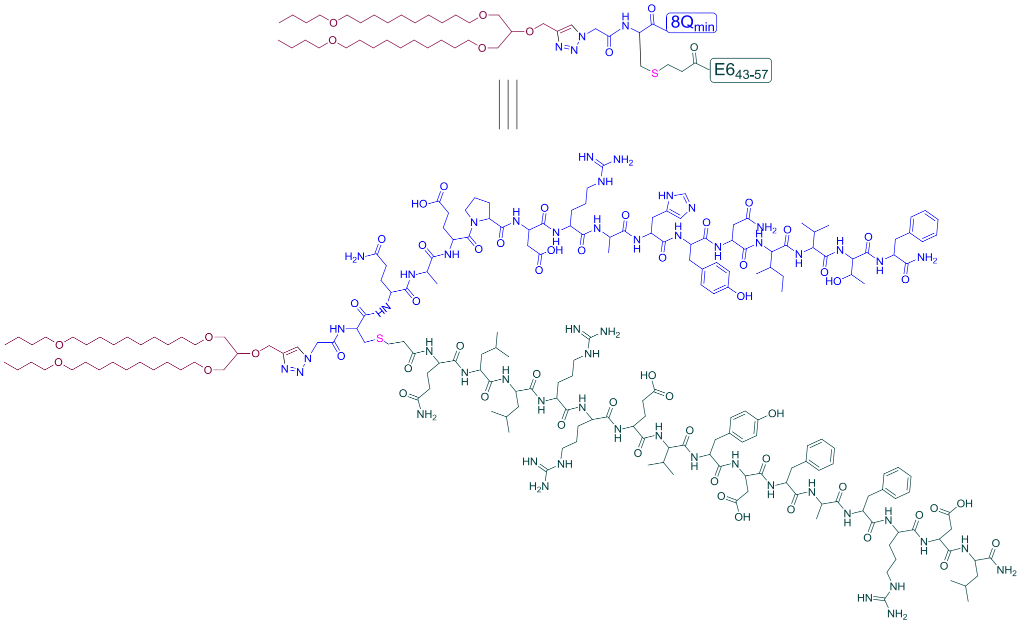

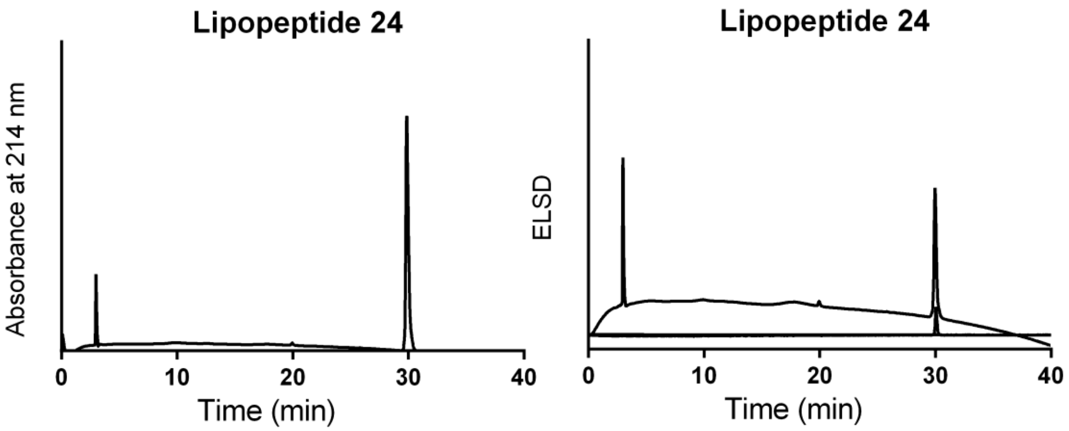

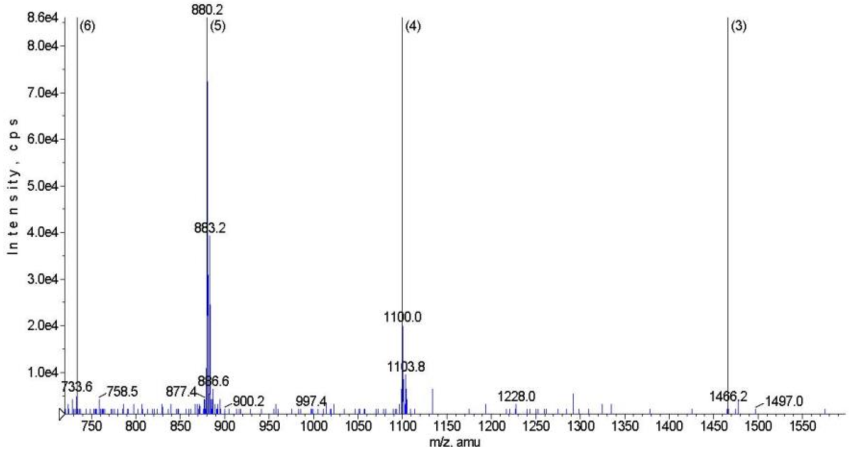

Mass spectrum of lipopeptide **24**

Structure of vaccine candidate lipopeptide 25

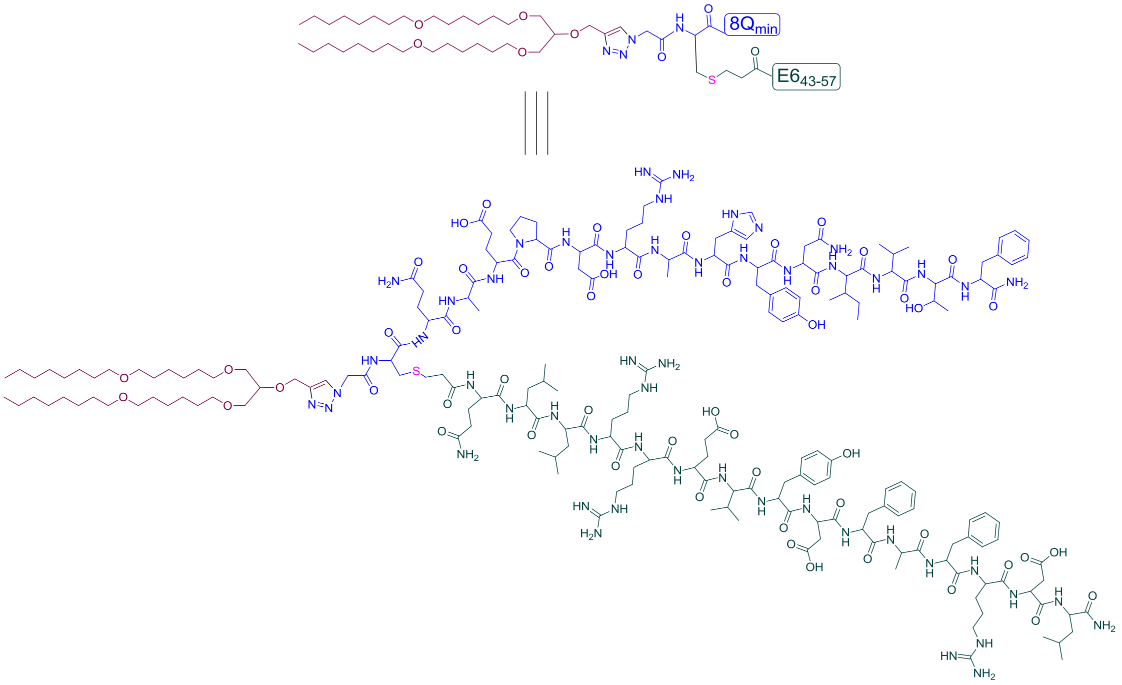

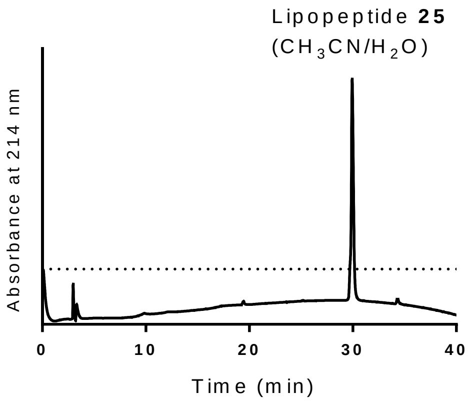

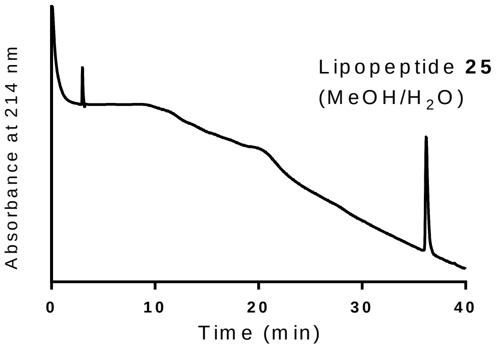

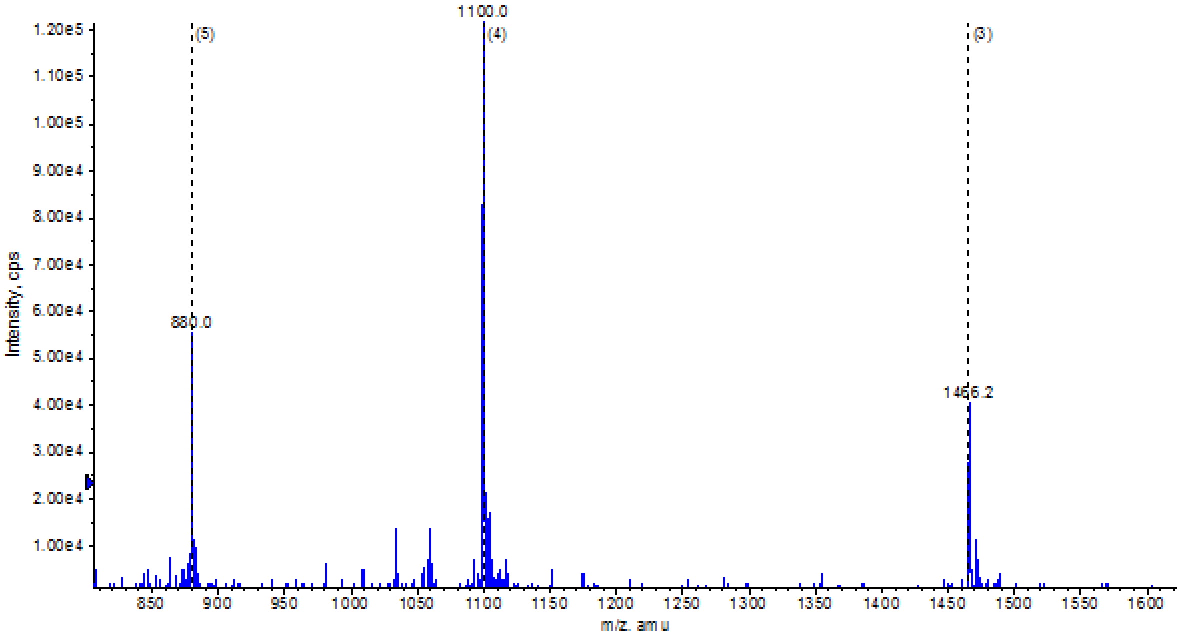

Mass spectrum of lipopeptide **25**

Structure of vaccine candidate lipopeptide 26

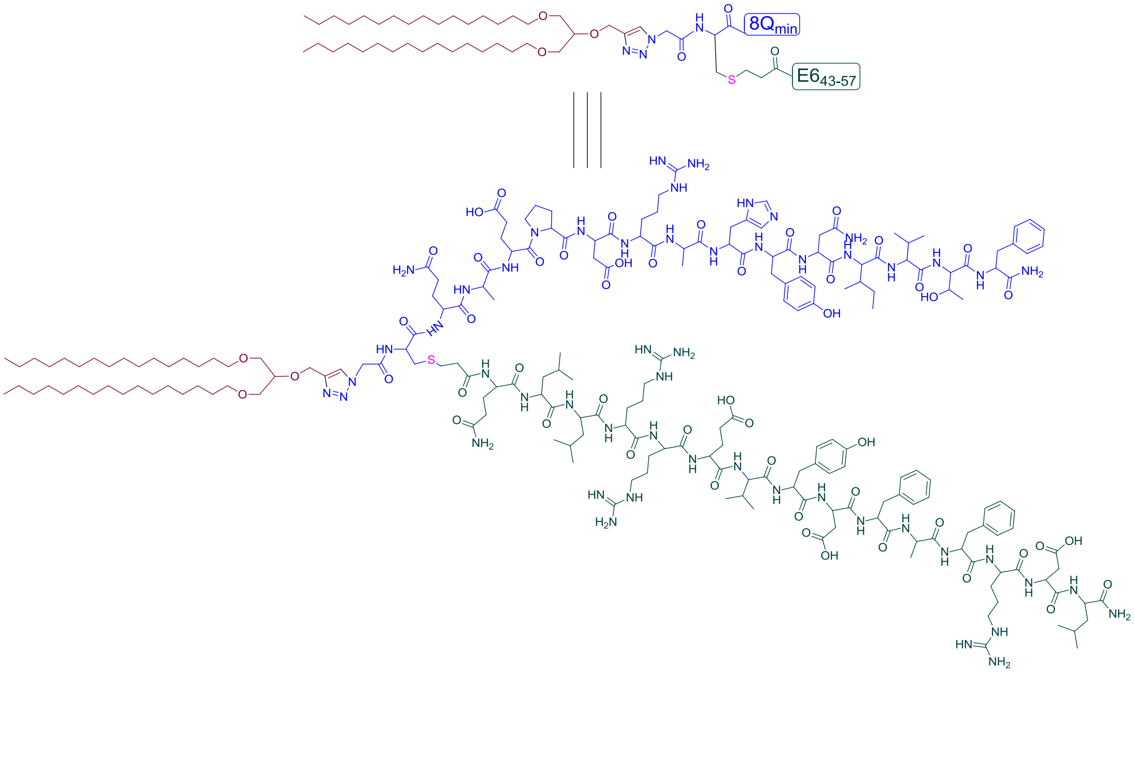

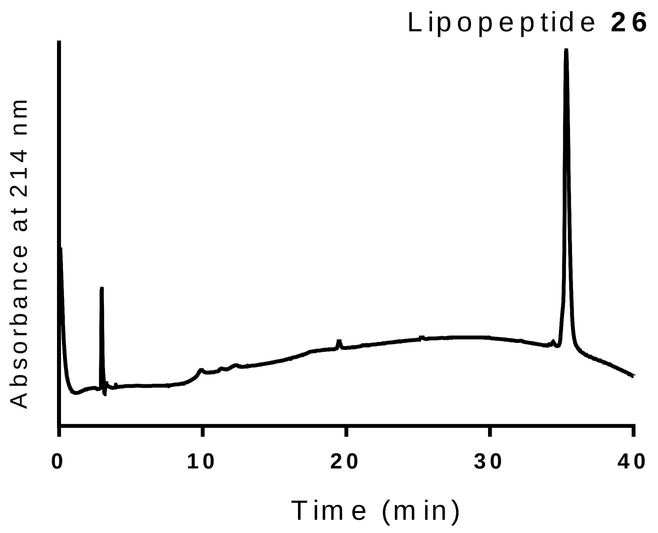

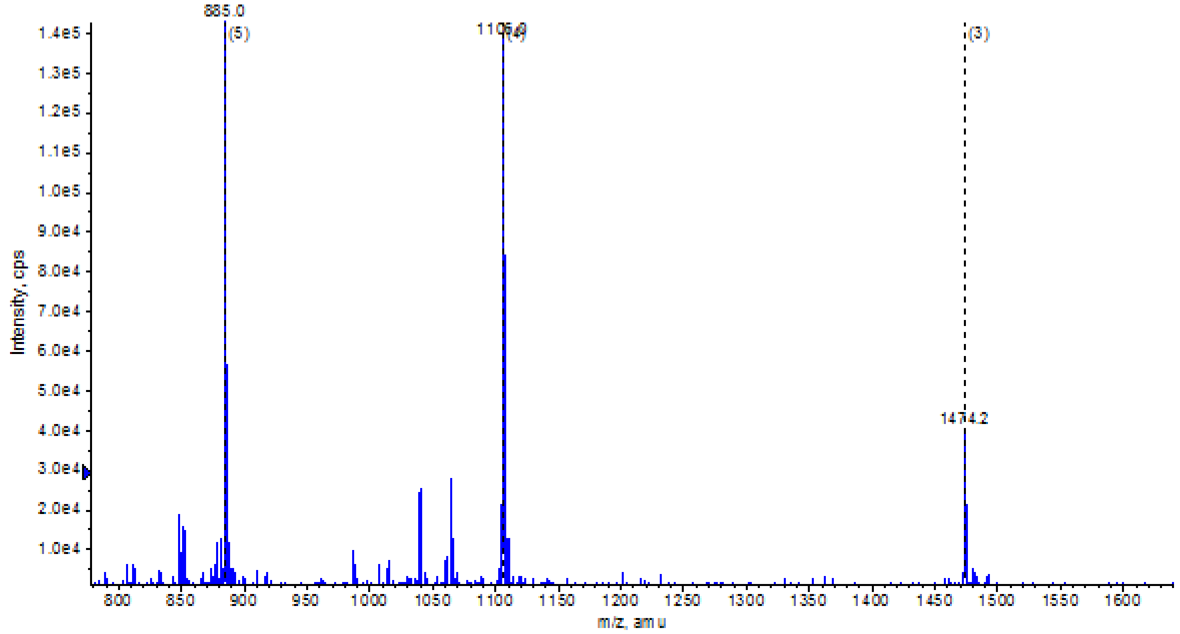

Mass spectrum of lipopeptide **26**

Structure of N-terminus $8Q_{min}$-azide.

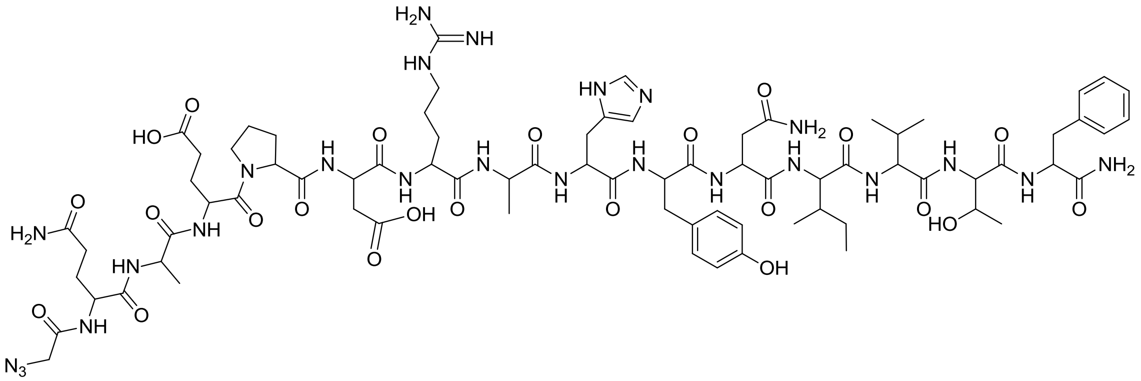

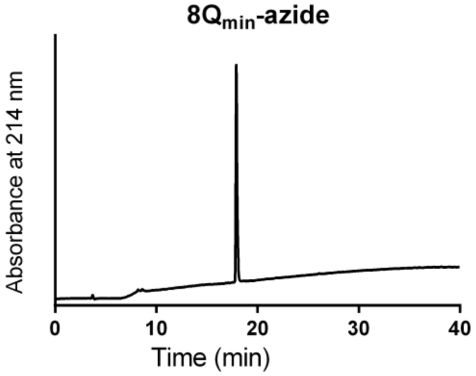

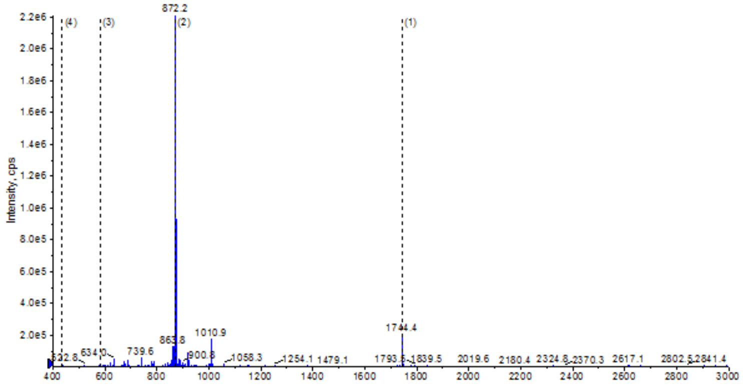

Mass spectrum of $8Q_{min}$-azide

Structure of N-terminus $E6_{43\text{-}57}$-azide.

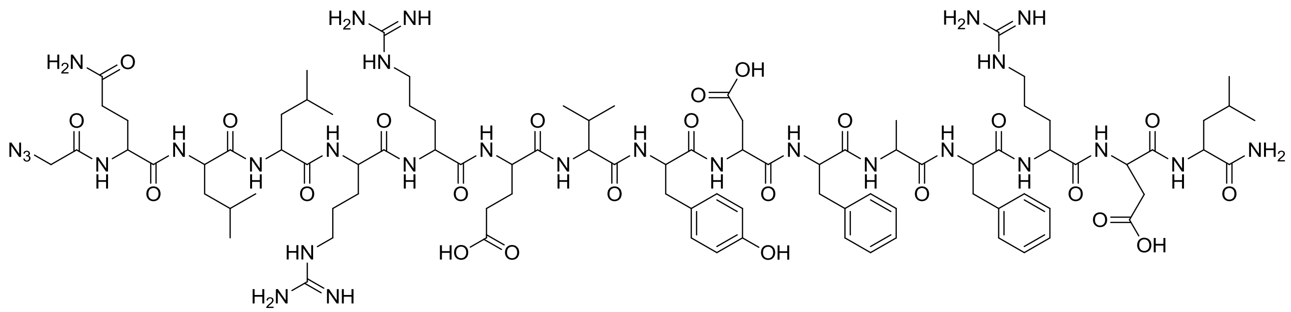

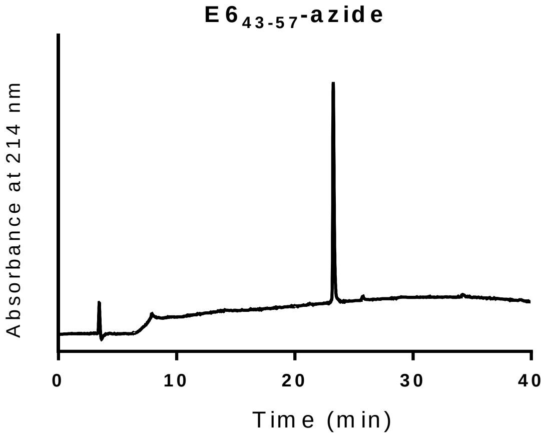

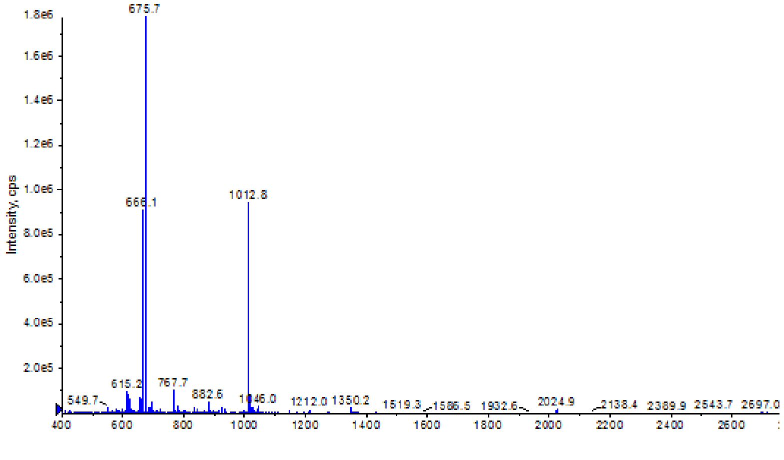

Mass spectrum of E6$_{43\text{-}57}$-azide

Structure of lipid 1 conjugated with $8Q_{min}$ 27

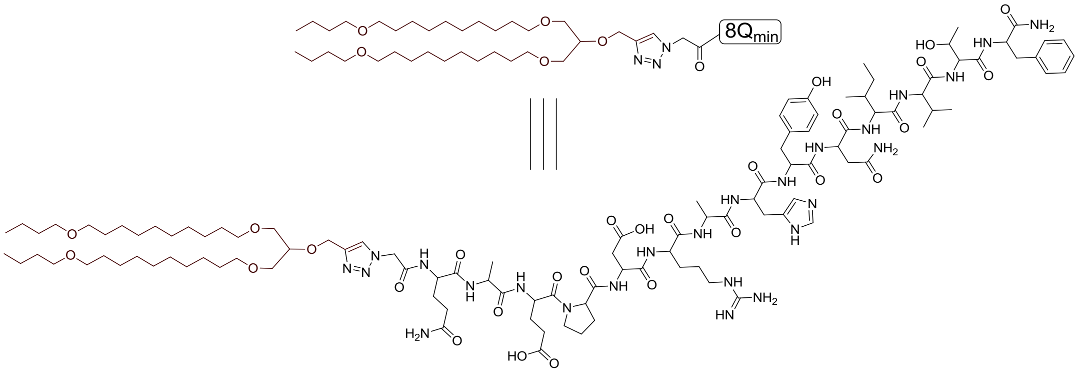

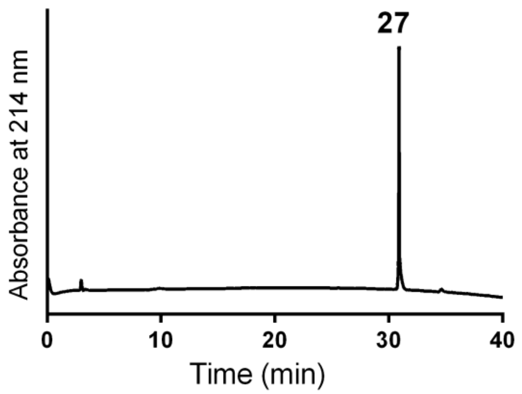

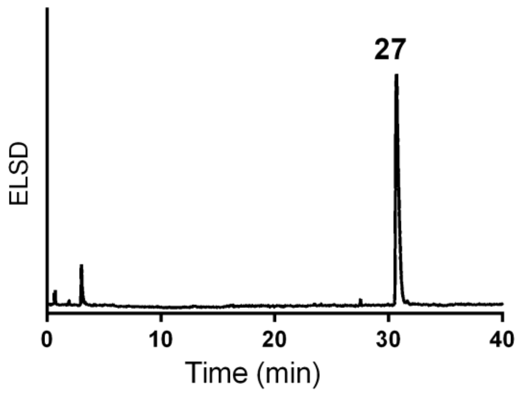

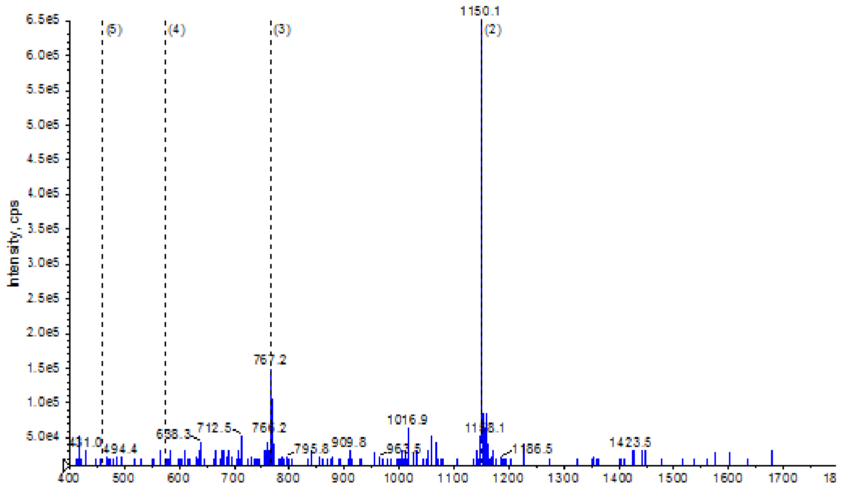

Mass spectrum of lipid **1** conjugated with $8Q_{min}$ (**27**)

Structure of lipid 1 conjugated with E6$_{43-57}$ (28)

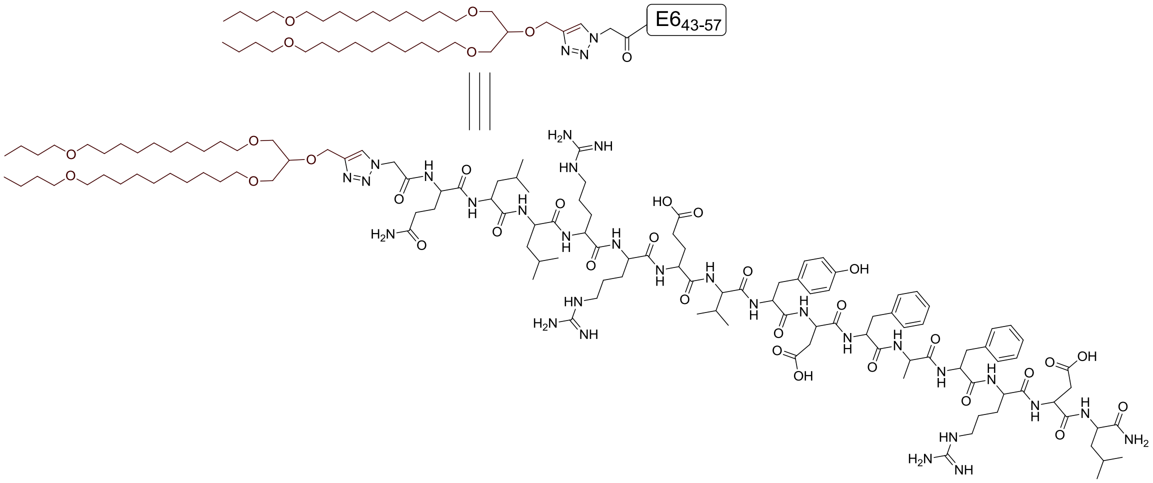

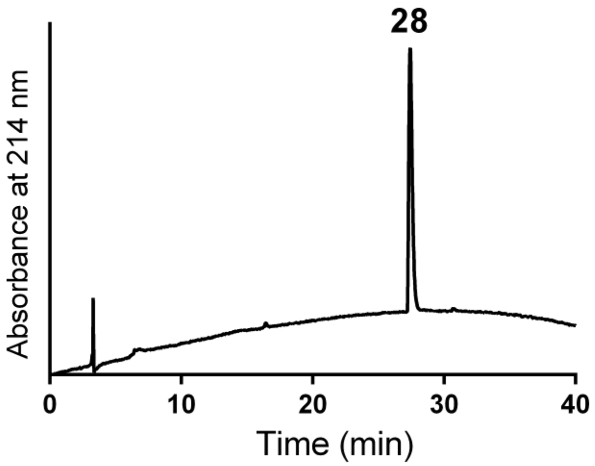

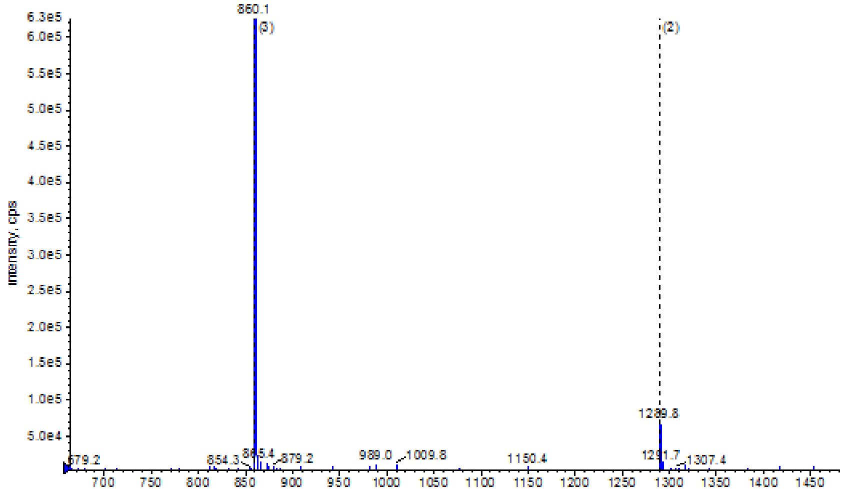

Mass spectrum of lipid **1** conjugated with E6$_{43-57}$ (**28**)

Structure of S-(2,3-dihydroxypropyl) Cysteine (Dhc-OH)

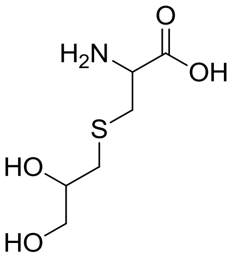

Structure of N-fluorenylmethoxycarbonyl-S-(2,3-dihydroxypropyl) cysteine (Fmoc-Dhc-OH)

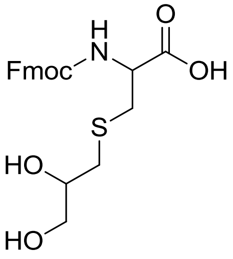

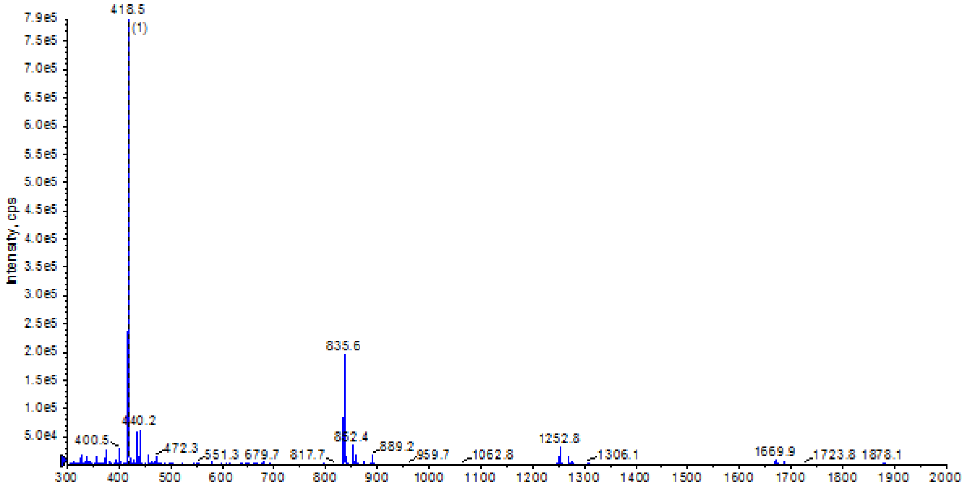

Mass spectrum of Fmoc-Dhc-OH

Structure of Pam2Cys-alkyne

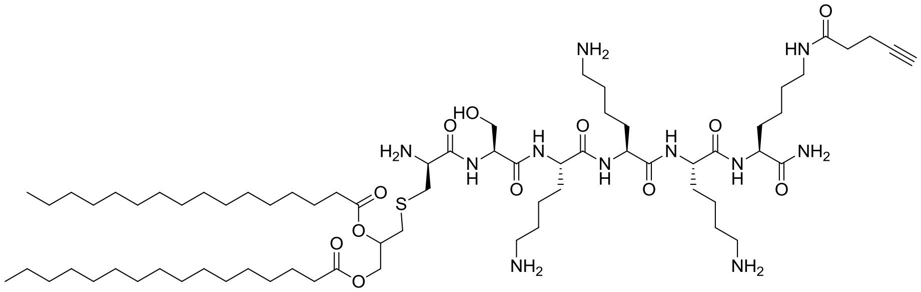

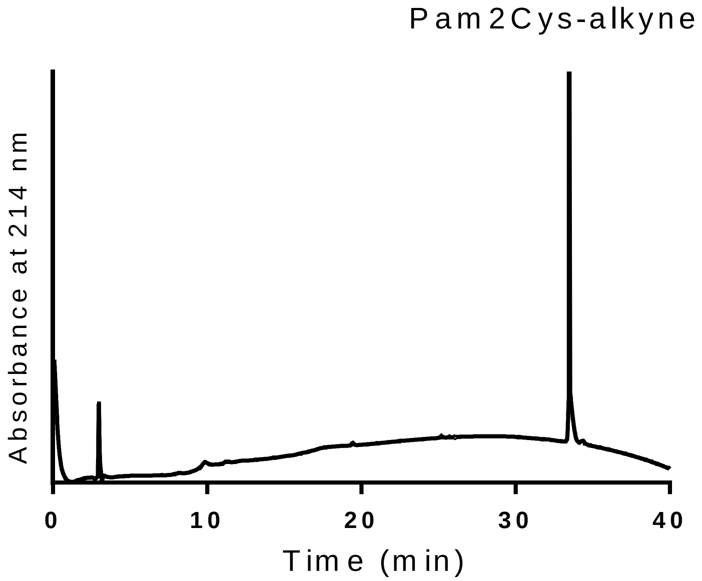

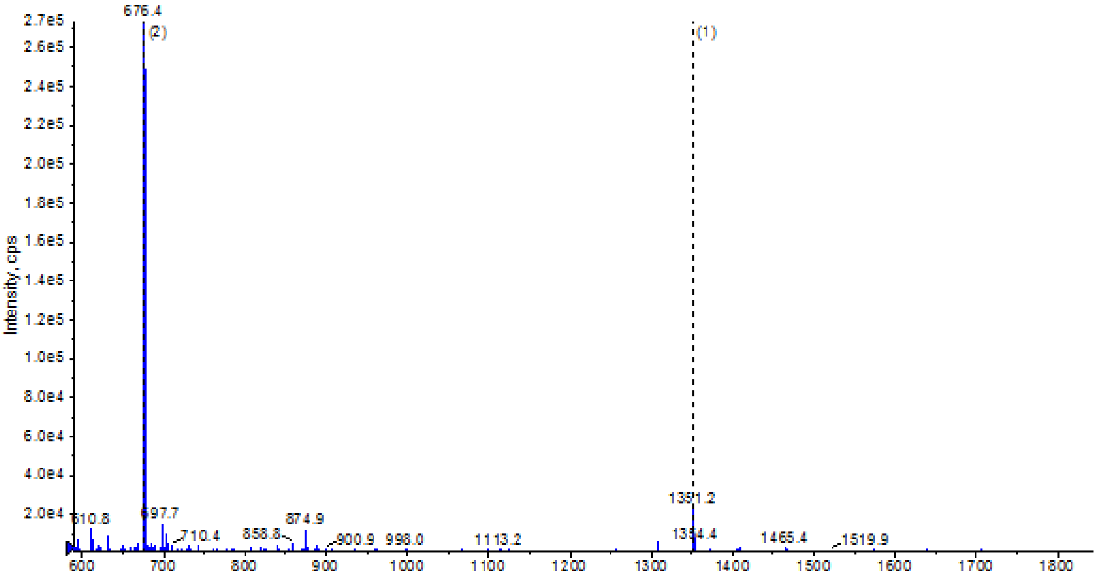

Mass spectrum of Pam2Cys-alkyne

Structure of 8Q$_{min}$/E6$_{43-57}$-Pam2Cys (29)

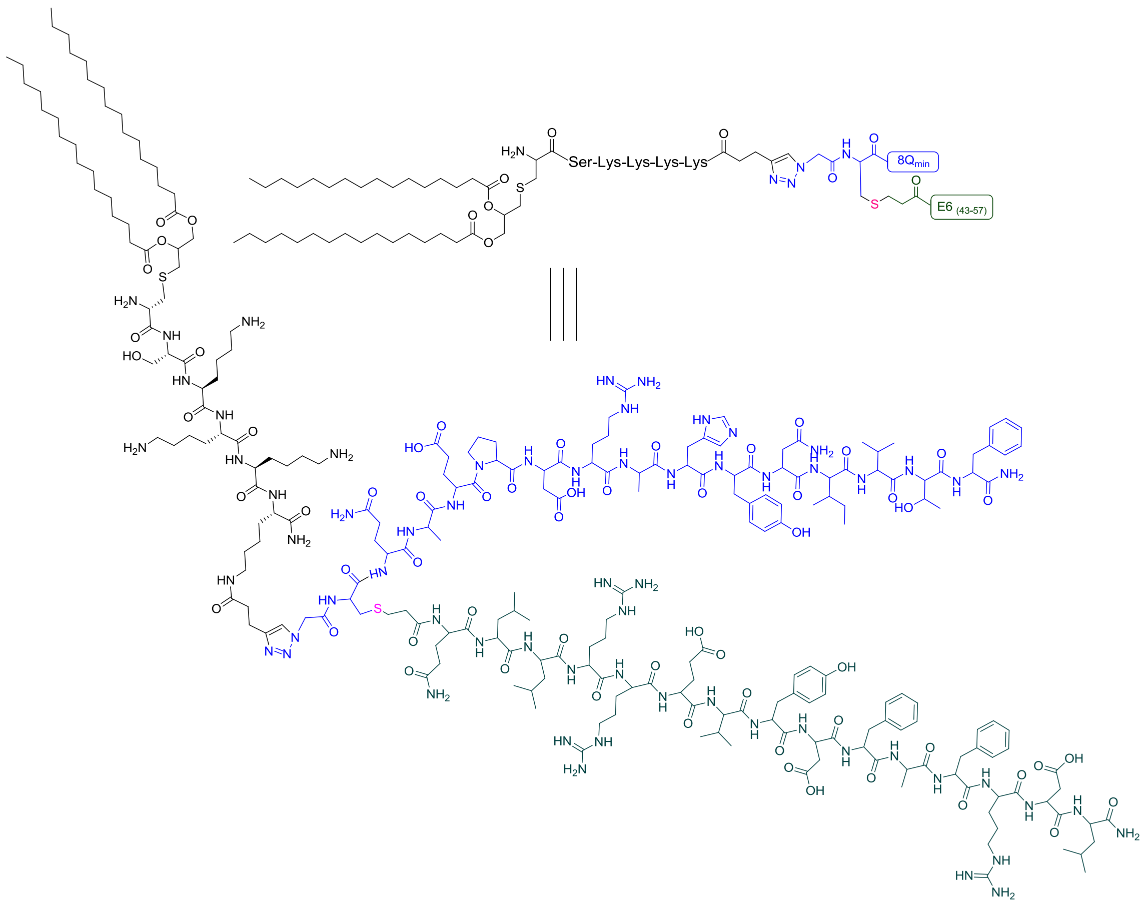

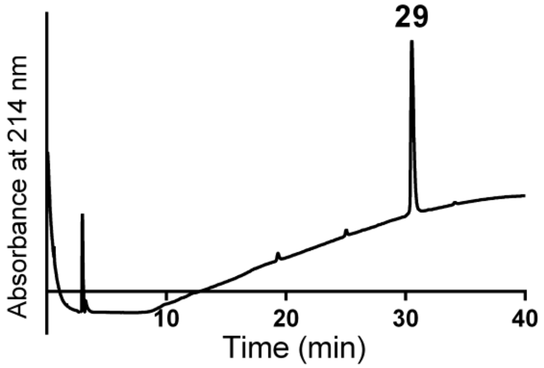

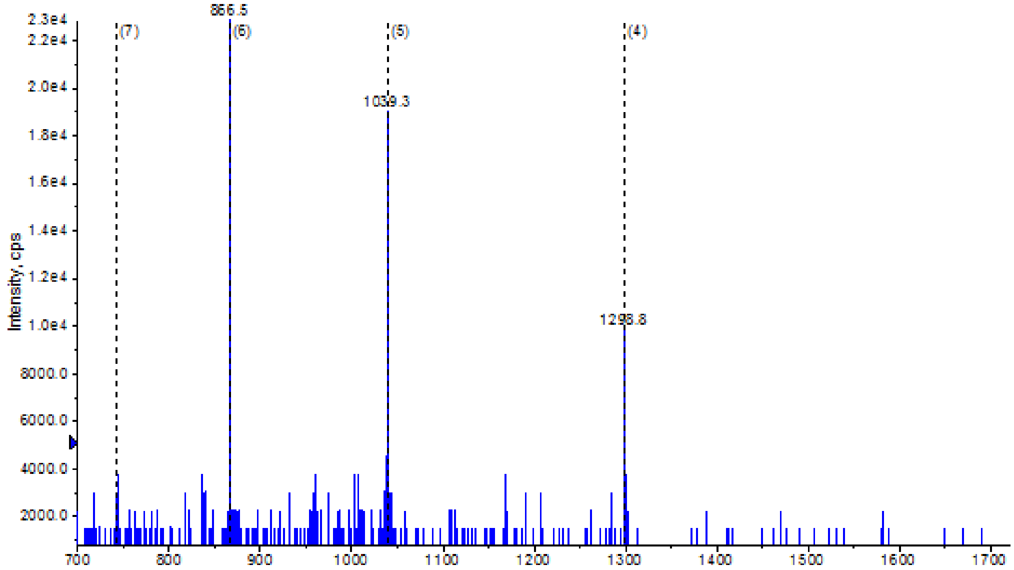

Mass spectrum of $8Q_{min}/E6_{43\text{-}57}$-Pam2Cys (**29**)

Model Compounds

Structure of N-terminal Model mercapto-azide (12).

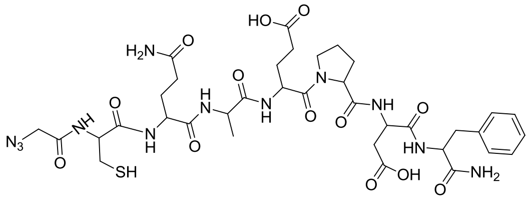

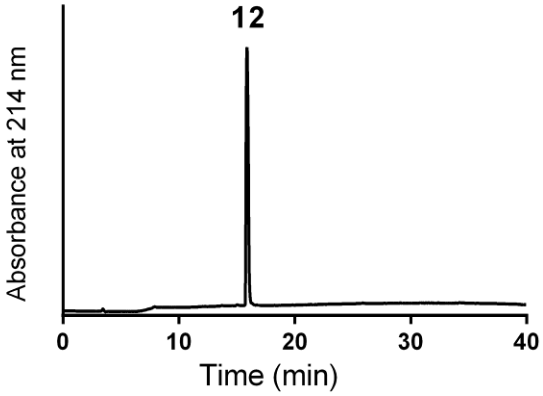

Structure of N-terminal acryloyl Model (13).

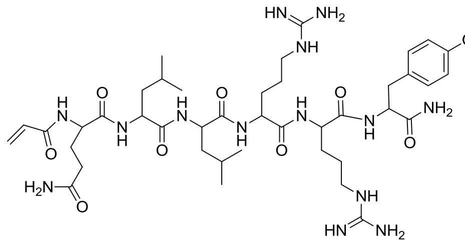

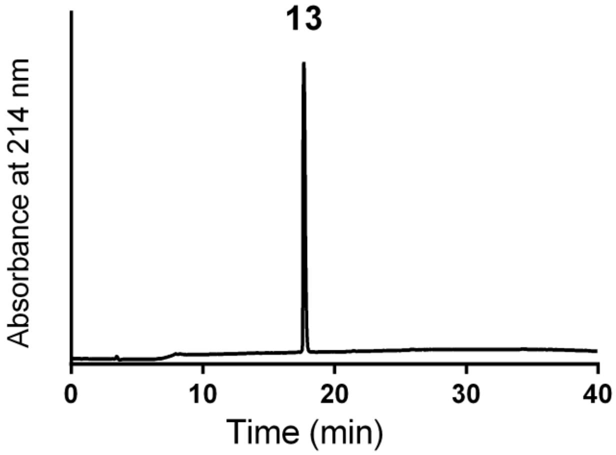

Structure of Model mercapto-acryloyl conjugation product (14)

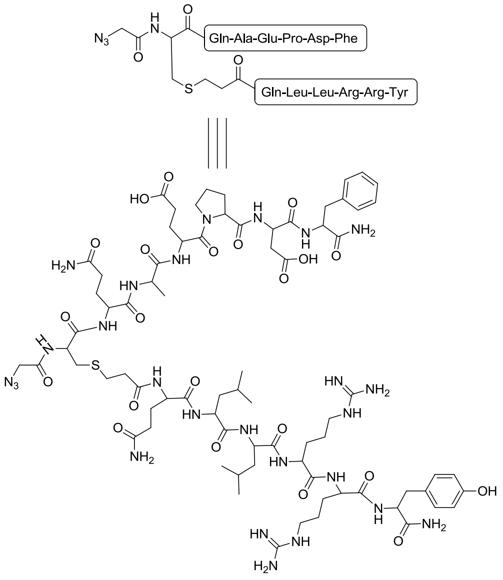

14

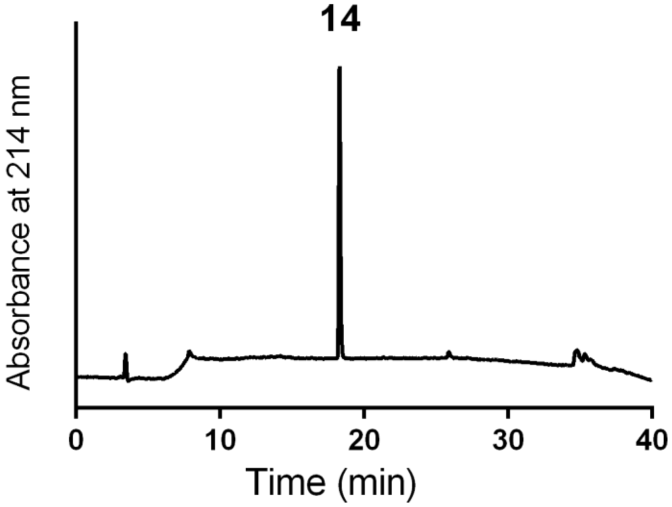

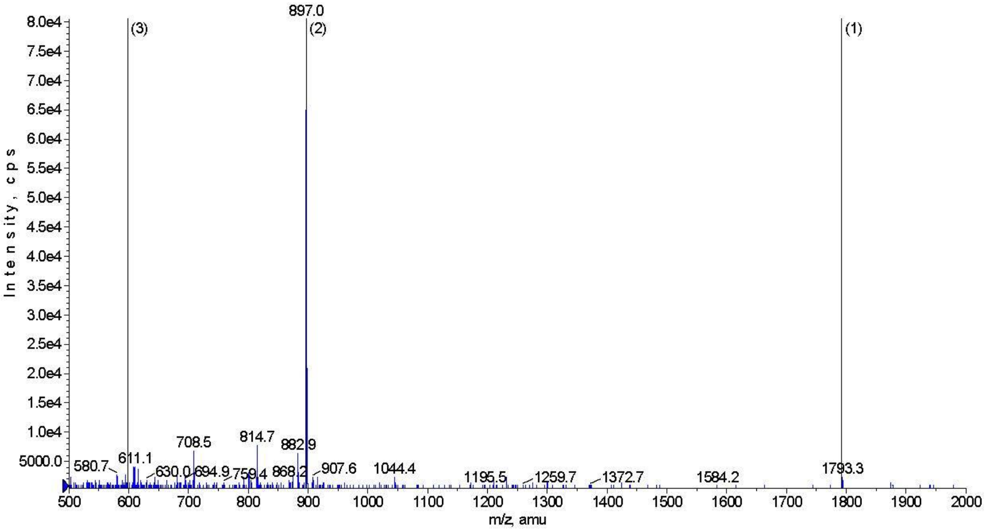

Mass spectrum of Model mercapto-acryloyl conjugation product (**14**)

Structure of compound 16.

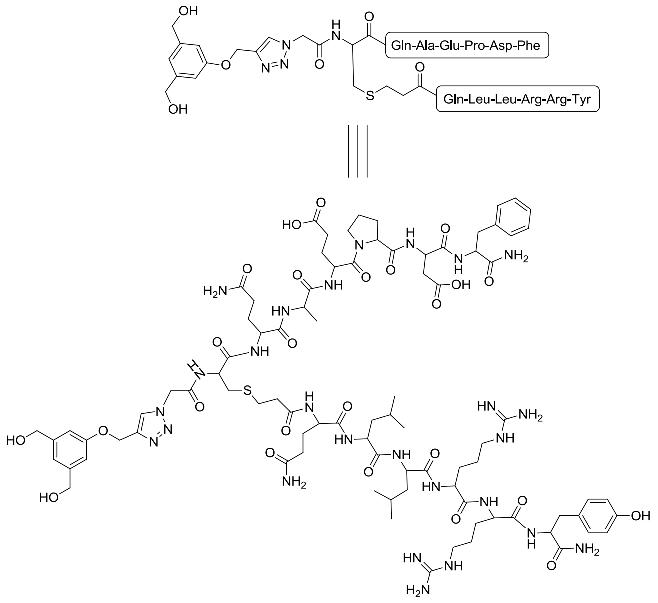

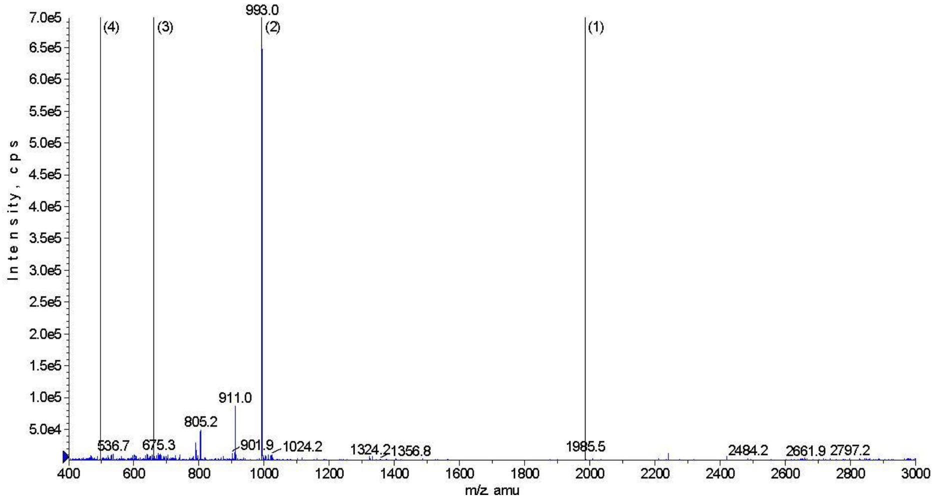

Mass spectrum of Model CuAAC product (**16**)

Structure of N-terminal Model mercapto-azide (17).

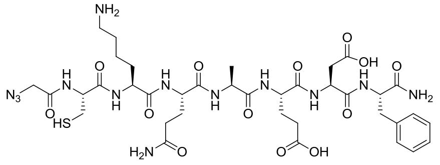

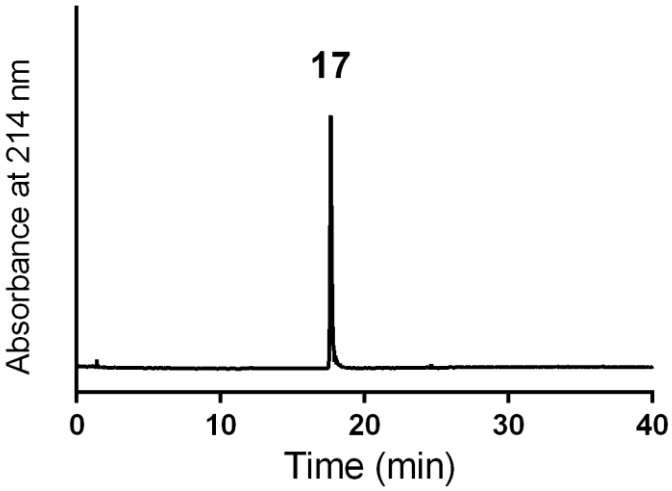

Structure of N-terminal acryloyl Model (18).

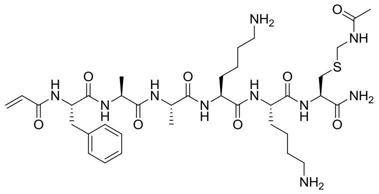

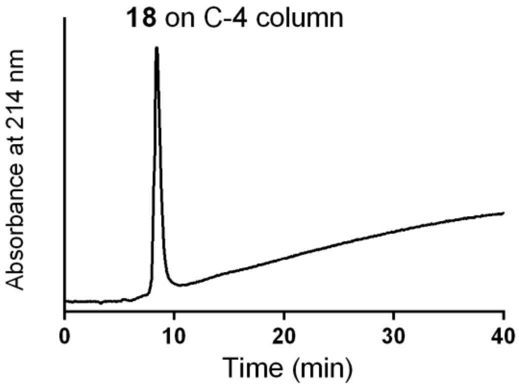

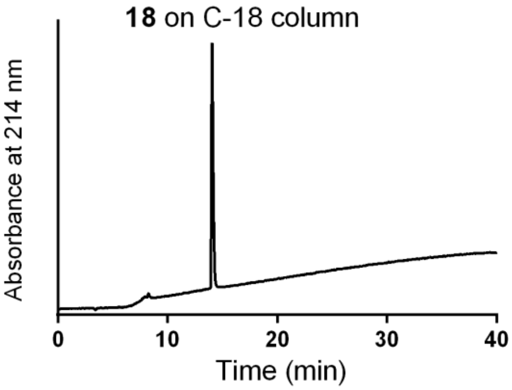

Structure of 20

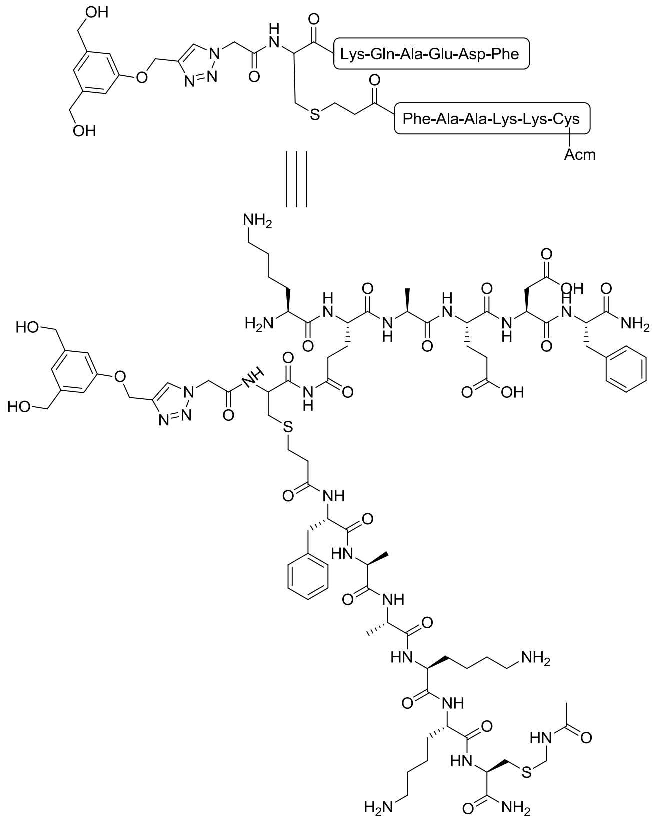

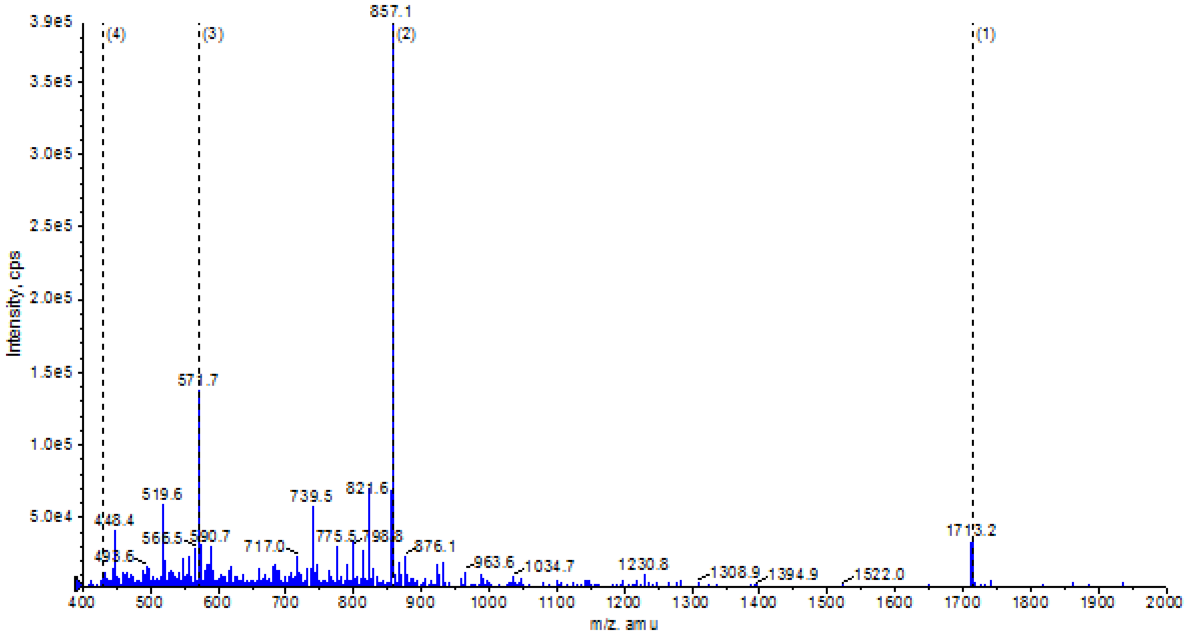

Mass spectrum of **19**

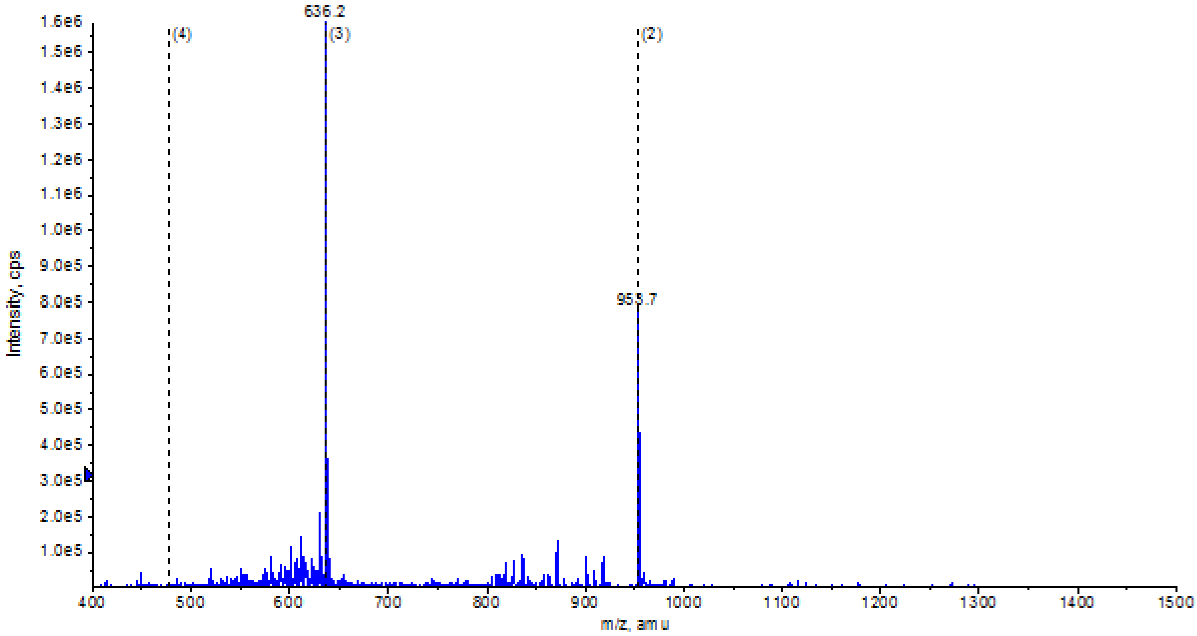

Mass spectrum of **20**
